# Systematic review of risk prediction models for catheter-associated urinary tract infection

**DOI:** 10.3389/fpubh.2026.1884134

**Published:** 2026-09-30

**Authors:** Zhang Mengqi, Jiang Yiting, Wu Xiaoying

**Affiliations:** The Affiliated Yongchuan Hospital of Chongqing Medical University, Chongqing, China

**Keywords:** catheter-associated urinary tract infection, hospital-acquired infection, prognostic model, risk prediction model, systematic review

## Abstract

**Objective:**

To systematically review and critically appraise published risk prediction models for catheter-associated urinary tract infection (CAUTI) in adult inpatients.

**Methods:**

Eight electronic databases were searched from inception to June 30, 2026. Two reviewers independently screened records and extracted data using the CHARMS checklist; risk of bias and applicability were assessed with PROBAST.

**Results:**

Thirteen reports describing 18 prediction models from 12 independent cohorts were included. All were retrospective, with sample sizes ranging from 140 to 122,386 and CAUTI incidence from 1.3 to 24.7%. Logistic regression predominated (11 of 13 reports). Derivation AUC values ranged from 0.611 to 0.993; nine exceeded 0.800. The most frequently identified predictors were age, catheter dwell time, diabetes mellitus, level of consciousness, and serum albumin. All 13 reports were rated high overall risk of bias by PROBAST, driven principally by temporal leakage — the incorporation of time-varying predictors into models intended for use at catheter insertion — insufficient events-per-variable ratios, data-driven variable selection, and near-universal absence of external validation. Only two reports conducted external validation (AUC = 0.758 and 0.970). Calibration was systematically underreported.

**Conclusion:**

Derivation AUC values are prone to optimism owing to pervasive methodological deficiencies, and current evidence is insufficient to support clinical implementation of any existing model. Future research should prioritize multicenter external validation over novel model development, with prospective designs, *a priori* predictor specification, adequate sample sizing, and full TRIPOD+AI-compliant reporting.

## 1 Introduction

Catheter-associated urinary tract infection (CAUTI) — defined as a urinary tract infection occurring in a patient with an indwelling catheter or within 48 h of its removal — accounts for approximately 70–80% of all hospital-acquired urinary tract infections and represents one of the most common healthcare-associated infections worldwide (1). Beyond prolonging hospitalization and increasing costs, CAUTI can progress to life-threatening complications such as sepsis (2). Prevention currently relies predominantly on standardized catheter management practices, including adherence to appropriate catheterization indications, minimizing catheter dwell time, and maintaining strict aseptic technique (3). However, considerable heterogeneity in patient baseline characteristics, underlying conditions, and treatment regimens results in substantial variation in individual CAUTI risk, even under comparable catheterization conditions. This variability means that uniform preventive measures are insufficient, and individual risk stratification is necessary (4). Early identification of high-risk patients therefore underpins effective prevention, and clinical prediction models offer a practical means of achieving this.

In recent years, advances in statistical modelling and machine learning have led to a proliferation of CAUTI risk prediction models (5, 6). However, considerable heterogeneity exists across these models in study design, predictor selection, modelling approach, and validation strategy, and their overall methodological quality and clinical applicability remain to be systematically evaluated. The CHecklist for critical Appraisal and data extraction for systematic Reviews of prediction Modelling Studies (CHARMS) and the Prediction Model Risk Of Bias ASsessment Tool (PROBAST) are established methodological frameworks for systematic reviews of prediction model studies, providing structured guidance for data extraction and risk of bias assessment, respectively (7, 8). To date, however, no systematic review of CAUTI risk prediction models has employed both frameworks in combination. We therefore conducted a systematic review of CAUTI risk prediction models in adult inpatients, using CHARMS for data extraction and PROBAST for methodological appraisal, to synthesize their modelling characteristics, predictor profiles, methodological quality, and predictive performance, and to inform priorities for future validation and clinical implementation.

## 2 Materials and methods

### 2.1 Problem formulation

This systematic review was conducted in accordance with the TRIPOD-SRMA reporting guideline (9) and was not prospectively registered; reasons are discussed in the Limitations section. The research question was framed using the PICOTS framework:

P (Population): Adult inpatients (aged ≥18 years) with an indwelling urinary catheter.

I (Index model): Development, validation, or updating of CAUTI risk prediction models.

C (Comparator): None.

O (Outcome): Occurrence of catheter-associated urinary tract infection (CAUTI).

T (Timing): During catheterization or within 48 h of its removal.

S (Setting): Acute care hospital settings, including intensive care units (ICUs), general wards, and surgical wards.

### 2.2 Inclusion and exclusion criteria

Inclusion criteria: (1) Studies enrolling hospitalized adult patients aged ≥18 years; (2) studies reporting the development, validation, or updating of a CAUTI risk prediction model; (3) cohort, case–control, or cross-sectional study designs.

Exclusion criteria: (1) Studies identifying risk factors for CAUTI without constructing a formal multivariable prediction model; (2) Studies published in languages other than Chinese or English; (3) Reports derived from the same cohort using identical modelling approaches. *Where multiple reports from the same cohort employed distinct modelling approaches, all were retained as separate model reports but counted as a single cohort in population-level analyses*; (4) Conference abstracts, studies for which full text was unavailable, or studies with insufficient data for extraction.

### 2.3 Literature search strategy

Eight electronic databases were searched from inception to June 30, 2026: PubMed, Web of Science, Embase, the Cochrane Library, CNKI, Wanfang Data, VIP, and CBM/SinoMed. Searches combined controlled vocabulary (subject headings) with free-text terms. Disease concepts included catheter-associated urinary tract infection and related terms; model concepts included prediction model, risk score, nomogram, and related terms, searched in both English and Chinese as appropriate. Complete reproducible search strategies for all eight databases, including platform, search date, full search strings, and records retrieved, are provided in Supplementary material S2. A total of 2,759 records were retrieved (PubMed = 195; Embase = 168; Web of Science = 1,219; Cochrane Library = 288; CNKI = 95; Wanfang Data = 444; VIP = 92; CBM/SinoMed = 258).

### 2.4 Literature screening and data extraction

Two reviewers (MZ and YT) independently screened all records at the title-and-abstract stage and at the full-text stage; disagreements were resolved through discussion or by consulting a third investigator (XY). Data extraction was performed independently by the same two reviewers using a structured form prespecified in accordance with the CHecklist for critical Appraisal and data extraction for systematic Reviews of prediction Modelling Studies (CHARMS) (8). Extracted items included first author, publication year, country, study population, study design, data source, total sample size, number of outcome events, cohort partitioning (development, internal validation, and external validation), modelling approach, missing data handling, variable selection method, number and identity of final model predictors, model performance metrics (including AUC with 95% confidence intervals where reported, calibration statistics, and Brier score), validation strategy, and model presentation format. All numerical values were independently verified against the source publications; discrepancies were resolved by returning to the original document or consulting a third investigator. Unreported values are indicated by “/” in the tables. To detect overlapping populations, all reports were cross-checked by institution, study period, sample size, and event count; where overlap was suspected, full publications were compared to determine cohort independence.

### 2.5 Risk of bias and applicability assessment

Risk of bias and applicability were independently assessed by two reviewers using the Prediction Model Risk of Bias ASsessment Tool (PROBAST) (7) across four domains: Participants (D1), Predictors (D2), Outcome (D3), and Analysis (D4). Assessment was conducted at the report level; where a report described multiple prediction models, a single domain-level judgment was assigned to the report as a whole, and this judgment consequently encompasses all models described within that report. Disagreements between reviewers were resolved through discussion or by consulting a third investigator.

### 2.6 Statistical analysis

Given the substantial heterogeneity across included reports, only descriptive analysis was performed. This heterogeneity was evident across five domains: (1) Population: study populations ranged from general inpatients to specialized subgroups, with CAUTI incidence ranging from 1.3 to 24.7%. (2) Modelling approach: logistic regression, decision trees, and machine learning algorithms were all represented, with final models incorporating 4–14 predictors. (3) Outcome definition: diagnostic criteria included CDC/NHSN criteria, Chinese national standards, and unspecified definitions, with varying ascertainment windows. (4) Validation strategy: approaches ranged from bootstrap resampling and split-sample validation to no validation. (5) Performance reporting: metrics were neither uniform nor consistently reported across reports.

Given this multidimensional heterogeneity, quantitative pooling of model performance metrics — such as a summary AUC — was not considered methodologically appropriate, and no meta-analysis was performed.

Sample size adequacy was assessed using method-appropriate criteria. For reports using standard logistic regression, the events-per-variable (EPV) criterion was applied, calculated as the number of outcome events in the development dataset divided by the number of candidate predictor parameters considered during model development — not the number retained in the final model, as the latter underestimates overfitting risk. EPV thresholds of 10 and 20 were used as reference benchmarks: EPV ≥ 10 represents the commonly cited minimum, and EPV ≥ 20 represents the more contemporary recommendation for stable logistic regression model development (10). Where authors explicitly listed variables entered into univariable or multivariable analysis, this count was used as the candidate variable denominator; where only final model predictors were reported, the candidate count was inferred from the variable selection description. For the decision tree report (11), EPV thresholds are not applicable to tree-based methods; adequacy was instead assessed by the absolute event count (*n* = 21), tree depth, and terminal node stability. For the machine learning report (5), EPV is similarly inappropriate for ensemble and deep learning methods; adequacy was assessed qualitatively from the large sample size (122,386 episodes), built-in regularization, and availability of an independent external validation dataset.

## 3 Results

### 3.1 Literature screening process and results

Thirteen reports met eligibility criteria, yielding 18 prediction models from 12 independent cohorts. One overlapping pair was identified (LIU Yuting 2023 and LI Yuping 2023, both from the same 537-patient NICU cohort); both are retained as distinct model reports given their different modelling approaches but counted once in population-level analyses. The study selection process is shown in Figure 1.

**Figure 1:**
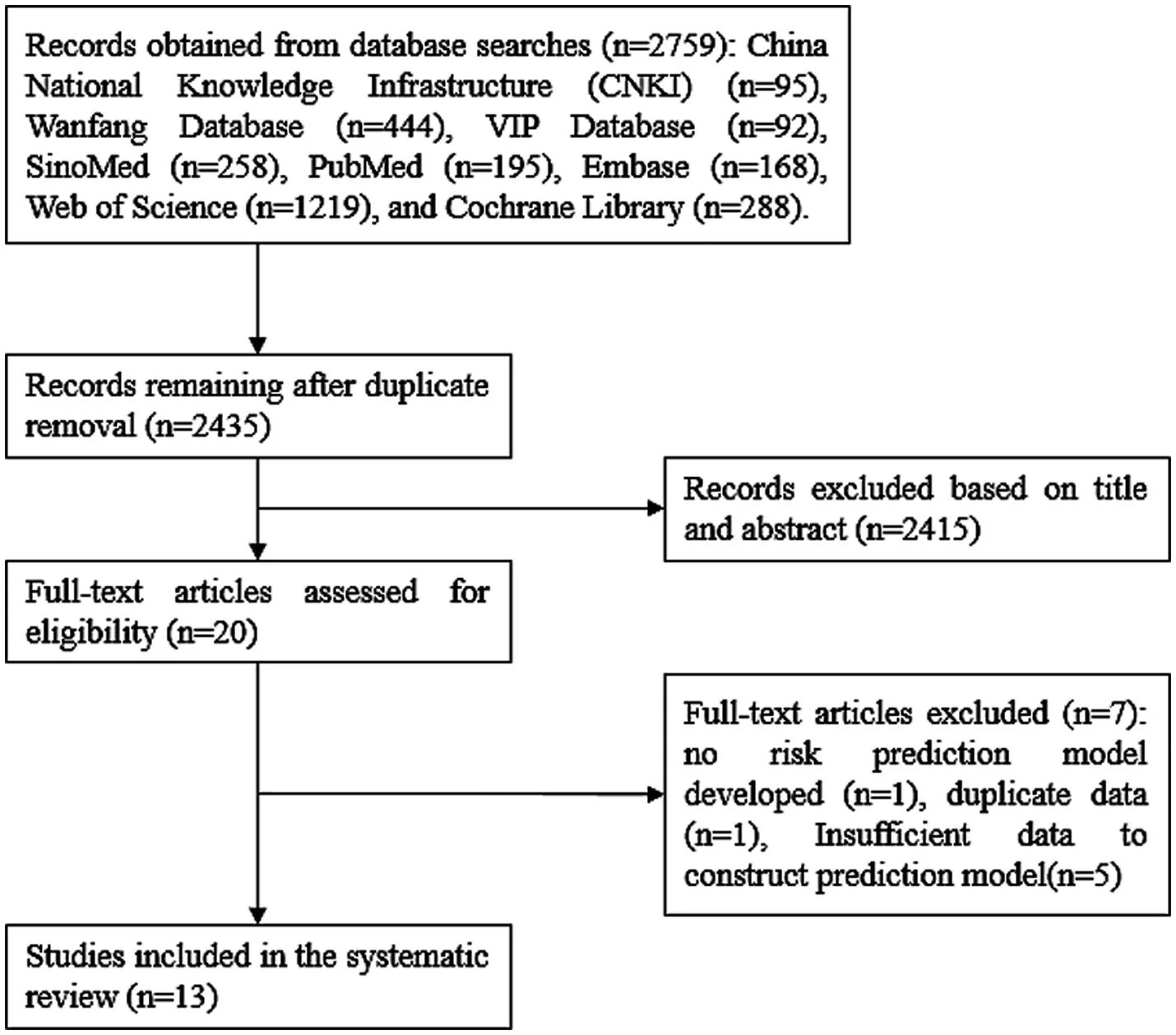
PRISMA flow diagram of study selection. A total of 2,759 records were identified through searches of eight electronic databases (CNKI, *n* = 95; Wanfang, *n* = 444; VIP, *n* = 92; SinoMed, *n* = 258; PubMed, *n* = 195; Embase, *n* = 168; Web of Science, *n* = 1,219; Cochrane Library, *n* = 288). Following removal of 324 duplicates, 2,435 records underwent title and abstract screening, of which 2,415 were excluded. The remaining 20 records were retrieved for full-text review; 7 were subsequently excluded for the following reasons: No prediction model developed or validated (*n* = 1), duplicate cohort (*n* = 1), and insufficient data for extraction (*n* = 5); the full list of excluded articles with individual reasons is provided in Supplementary material S3. Ultimately, 13 reports met all eligibility criteria and were included in the systematic review.

### 3.2 Basic characteristics of included reports

General characteristics of the 13 included reports are summarized in Table 1.

**Table 1:** Characteristics of included reports and their source cohorts.

Included report	Country	Study design	Study population	Data source	Model type
Liu Huiying 2025 (16)	China	Retrospective cohort study	Patients with stroke undergoing indwelling catheterization	Zhongshan Hospital of Traditional Chinese Medicine Affiliated to Guangzhou University of Chinese Medicine	Development and validation
Li Mengchao 2025 (20)	China	Retrospective cohort study	Postoperative patients with spinal cord injury undergoing indwelling catheterization	Department of Spinal Surgery, Affiliated Hospital of Jiujiang University	Development and validation
Sufriyana 2025 (5)	Taiwan	Retrospective cohort study	Patients undergoing indwelling catheterization	Clinical Research Database of Taipei Medical University	Development and validation
Zhu Rui 2024 (19)	China	Retrospective cohort study	Older critically ill patients undergoing indwelling catheterization	Jiangsu Provincial People’s Hospital	Development and validation
Sun Qing 2024 (17)	China	Retrospective case–control study	Hospitalized patients undergoing indwelling catheterization	A tertiary Class A hospital in Shandong Province	Development and validation
Wang Fang 2023 (13)	China	Retrospective cohort study	Postoperative patients undergoing radical resection of cervical cancer	The First Affiliated Hospital of Army Medical University of China	Development and validation
Liu Yuting 2023* (11)	China	Retrospective cohort study	Patients undergoing indwelling catheterization in the Neurological Intensive Care Unit (NICU)	Subei People’s Hospital, Clinical Medical College of Yangzhou University	Development only
Li Yuping 2023* (6)	China	Retrospective cohort study	Patients undergoing indwelling catheterization in the NICU	Subei People’s Hospital, Clinical Medical College of Yangzhou University	Development and validation
Xia Liyao 2022 (14)	China	Retrospective cohort study	Postoperative patients with gynecological malignant tumors undergoing indwelling catheterization	Cancer Hospital Affiliated to University of Chinese Academy of Sciences	Development and validation
Lü Yangyang 2022 (21)	China	Retrospective case–control study	Patients undergoing indwelling catheterization in the emergency department	The Second Affiliated Hospital of Wenzhou Medical University	Development only
Liao Liping 2022 (18)	China	Retrospective case–control study	Patients with craniocerebral injury undergoing indwelling catheterization	Chongqing Emergency Medical Center	Development and validation
Li Zhe 2021 (15)	China	Retrospective case–control study	Neurosurgical patients undergoing indwelling catheterization	A tertiary Class A hospital in Shanxi Province	Development only
Eun Young Hur 2019 (12)	South Korea	Retrospective case–control study	Patients undergoing indwelling catheterization	Electronic health records from a teaching hospital in Seoul, South Korea	Development and validation

^*^: These two reports were derived from the same study cohort (537 patients in the Neurological Intensive Care Unit) at Subei People’s Hospital, Yangzhou University. Both are retained as separate model reports owing to their distinct modeling approaches, but are counted as a single cohort in all population-level analyses. Systematic cross-checking of all 13 included reports identified no additional overlapping cohorts.

### 3.3 Development of prediction models

The 13 included reports described a total of 18 CAUTI risk prediction models. Sample sizes ranged from 140 to 122,386 participants, and the number of outcome events ranged from 21 to 17,506. Logistic regression was the predominant modelling approach, used in 11 reports (6, 12–21); one report applied five machine learning algorithms (5), and another employed two decision tree algorithms (11). Two models retained continuous predictors in their original form (16, 21); the remaining 16 dichotomized or categorized continuous variables. Regarding missing data: two reports excluded participants with missing values (6, 17), one excluded predictors with missing-not-at-random patterns (5), and the remaining ten did not describe their approach. No report used imputation. With respect to variable selection, nine reports used univariable or data-driven pre-screening as the primary method of predictor selection (6, 12, 14–16, 18–21); one report applied conditional logistic regression with a pragmatic two-threshold screening approach (17); one report adopted LASSO regression (13); one report employed structural causal modelling with Benjamini-Hochberg multiple testing correction (5); and one report used decision tree-based variable selection (11). Final models incorporated between 4 and 14 predictors; the most frequently included were age, catheter dwell time, diabetes mellitus, impaired consciousness, and hypoalbuminemia. The intended prediction time point varied across reports. Ten described models for use at or near catheter insertion or hospital admission (6, 12–16, 18–21); one used a case–control design with no explicit prediction time point (17); one employed a daily repeated-measures framework (5); and one described an admission-time decision tree that nonetheless incorporated catheter dwell time — a time-varying variable — as a splitting criterion, creating an internal inconsistency (11). These differences in prediction time point are clinically important and preclude direct comparison of model performance across reports. Development characteristics of all included models are summarized in Table 2.

**Table 2:** Modeling and development characteristics of included CAUTI risk prediction models.

Included report	Model development method	Total sample size (D/I/E, cases)	Number of outcome events (D/I/E, cases)	Variable handling method	Missing data		Variable selection method	Intended prediction time point	Predictors	
					Number of missing cases	Handling method			Number in final model	Content of predictors
Liu Huiying 2025 (16)	LR	699 (489/210/−)	84 (57/27/−)	X	/	/	①	At hospital admission; stroke inpatients with indwelling catheters	5	Age, NIHSS score, duration of indwelling catheterization, admission to intensive care unit (ICU), combined use of antibiotics
LI Mengchao 2025 (20)	LR	140 (140/−/−)	32 (32/−/−)	Y	/	/	①	At catheter insertion or admission; post-operative spinal cord injury	5	Stress hyperglycemia, duration of indwelling catheterization, neurogenic bladder, ASIA score, serum albumin level
Sufriyana 2025 (5)	RR, DT, RF, XGBoost, DI-VNN	122,386(81,010/20,253/21,123)	17,506(10,676/2,756/4,074)	Y	/	Direct exclusion	④	Daily throughout catheterisation episode	14	Age, length of ICU stay, length of hospital stay, gender, diabetes mellitus, immobility, hypertension, diabetic nephropathy, HIV infection, malignant tumor, urinary system disorders, >2 urinary tract infection (UTI) diagnoses in the past 6 months or >3 in the past year, neurological disorders, indication for catheterization
Zhu Rui 2024 (19)	LR	8,905 (8,905/−/−)	114 (114/−/−)	Y	/	/	①	At ICU admission or catheter insertion; older adults critically ill	5	Age, consciousness status, renal dysfunction, duration of indwelling catheterization, number of catheterizations
Sun Qing 2024 (17)	LR	1,042 (1,042/−/−)	521 (521/−/−)	Y	/	Direct exclusion	③	At catheter insertion or admission	4	Duration of catheterization, length of ICU stay, neurological diseases, number of antibiotic types
Wang Fang 2023 (13)	LR	380 (380/−/−)	88 (88/−/−)	Y	/	/	⑤	At surgery or catheter insertion; cervical cancer	5	Age, history of preoperative chemotherapy, history of urinary tract infection, re-catheterization, duration of indwelling catheterization
Liu Yuting 2023 (11)	CHAID, CRT	537 (537/−/−)	21 (21/−/−)	Y	/	/	②	At NICU admission	9	Age, GCS score, seizure on admission, admission in summer, mechanical ventilation use, number of antibiotic types, serum albumin, female gender, duration of catheterization
Li Yuping 2023 (6)	LR	537 (389/148/−)	21 (17/4/−)	Y	47	Direct exclusion	①	At NICU admission; 3 of 4 predictors assessable at admission	4	Age, length of stay in neurological intensive care unit (NICU), seizure on admission, serum albumin level
Xia Liyao 2022 (14)	LR	800 (600/200/−)	163 (124/39/−)	Y	/	/	①	At postoperative catheter insertion; gynaecologic malignancy	4	Age, intraoperative urinary system injury, postoperative bladder irrigation, duration of indwelling catheterization
Lü Yangyang 2022 (21)	LR	1,780 (1,780/−/−)	142 (142/−/−)	X	/	/	①	At catheter insertion; emergency department	5	Age, body mass index (BMI), duration of indwelling catheterization, glycosylated hemoglobin (HbA1c), serum albumin
Liao Liping 2022 (18)	LR	815 (570/245/−)	121 (80/41/−)	Y	/	/	①	At catheter insertion or ICU/neurosurgery admission; TBI	5	Age, GCS score, duration of indwelling catheterization, length of ICU stay, stress hyperglycemia
Li Zhe 2021 (15)	LR	300 (300/−/−)	74 (74/−/−)	Y	/	/	①	At catheter insertion; neurosurgery ward	8	Female gender, age, diabetes mellitus, emergency admission, prophylactic antibiotic use, consciousness disturbance, admission to ICU, D-dimer
Eun Young Hur 2019 (12)	LR	4,060 (2,150/645/1,265)*	816 (430/133/253)	Y	/	/	①	During hospitalization; daily updated risk scores	8	Length of hospital stay, admission to ICU, physical activity dependence, highest neutrophil count, lowest serum sodium level, lowest serum albumin level, highest blood urea nitrogen level, duration of indwelling catheterization

D, Development cohort; I, Internal validation cohort; E, External validation cohort; X, Retained as continuous variables; Y, Converted to categorical variables; ① Univariable or data-driven pre-screening followed by multivariable analysis (including backward stepwise selection); ② Decision tree-based variable selection; ③ Conditional logistic regression with pragmatic two-threshold screening; ④ Structural causal modelling with Benjamini-Hochberg multiple testing correction; ⑤ LASSO regression analysis; LR, Logistic regression; RF, Random forest; RR, Ridge regression; DT, Decision tree; CHAID, Chi-squared automatic interaction detector; CRT, Classification and regression tree; XGBoost, Extreme gradient boosting; DI-VNN, Deep integrated variational neural network; /, Value not reported in the original publication. All numerical values in this table were independently verified by two reviewers against the original source documents. ^*^: Eun Young Hur 2019 conducted two successive temporal external validations: algorithm validation cohort (*n* = 1,265, represented above) and a separate system validation cohort (*n* = 1,000, not included in the total N above). Total across all datasets: 5,060.

### 3.4 Performance and validation of prediction models

Three reports described model development only (11, 15, 21), while the remaining ten reports also performed internal validation (5, 6, 12–14, 16–20); of these, only two reports conducted external validation (5, 12). Twelve reports assessed discrimination using the area under the receiver operating characteristic curve (AUC) (5, 6, 11, 13–21). In derivation cohorts, AUC values ranged from 0.611 to 0.993; nine reports described models with derivation AUC values exceeding 0.800 (6, 11, 13, 15, 16, 18–21). Ten reports conducted internal validation; however, only four reported AUC values for the validation cohort: 0.758 (16), 0.790 (14), 0.888 (6), and 0.930 (18), respectively. The validation-set AUC of 0.888 reported by LI Yuping 2023 (6) was based on only 4 CAUTI events, rendering this estimate unreliable. The remaining six reports conducted internal validation without reporting discrimination statistics. Only two reports conducted external validation: Hur Eun Young 2019 used temporal validation within a single institution (AUC = 0.970) (12), and Sufriyana 2025 used geographic validation in an independent hospital, where the best-performing model achieved an AUC of 0.758 (5). These figures address different dimensions of generalizability and cannot be directly compared. External validation evidence therefore remains very limited. With respect to calibration, four reports provided no calibration assessment of any kind (12, 14, 15, 21). Among the remaining nine reports, four reported calibration curves only (6, 13, 16, 17); three reported both calibration curves and the Hosmer-Lemeshow (H-L) test (18–20); one reported the Brier score only (11); and one reported both calibration curves and Brier scores (5). All reported H-L test *p*-values exceeded 0.05. No report provided a complete calibration assessment encompassing calibration slope, intercept, and Brier score simultaneously. With respect to presentation format, nomograms were used in eight reports (5, 6, 13, 16–20), regression equations in two (15, 21), and risk scoring systems in three (11, 12, 14). Performance and validation characteristics of all included models are summarized in Table 3.

**Table 3:** Predictive performance and validation of included CAUTI risk prediction models.

Included report	Discrimination			Calibration	Model validation method	Model presentation
	AUC (95%CI) – Development	AUC (95%CI) – Internal validation	AUC (95%CI) –External Validation			
Liu Huiying 2025 (16)	0.857 (0.801, 0.914)	0.758 (0.661, 0.855)	/	Calibration curve	Bootstrap internal validation; Random split internal validation	Nomogram
Li Mengchao 2025 (20)	0.993 (0.984, 1.000)	/	/	Calibration curve; H-L test (*p* = 0.845 > 0.05)	Bootstrap internal validation	Nomogram
Sufriyana 2025 (5)	RR: 0.752 (0.750, 0.754) DT: 0.788 (0.786, 0.789) RF: 0.788 (0.786, 0.790) XGBoost: 0.787 (0.785, 0.789) DI-VNN: 0.611 (0.608, 0.613)	/	RF: 0.758	Calibration curve; Brier score: 0.453	Random split internal validation; Spatial external validation	Nomogram
Zhu Rui 2024 (19)	0.802 (0.796, 0.809)	/	/	Calibration curve; H-L test (*p* = 0.581 > 0.05)	Bootstrap internal validation	Nomogram
Sun Qing 2024 (17)	0.677	/	/	Calibration curve	Bootstrap internal validation	Nomogram
Wang Fang 2023 (13)	0.810 (0.759, 0.861)	/	/	Calibration curve	Bootstrap internal validation	Nomogram
Liu Yuting 2023 (11)	CHAID: 0.852 CRT: 0.920	/	/	Brier score (CRT: 0.026, CHAID: 0.032)	/	Risk scoring system
Li Yuping 2023 (6)	0.855	0.888	/	Calibration curve	Random split internal validation	Nomogram
Xia Liyao 2022 (14)	0.780	0.790 (0.720, 0.870)	/	/	Random split internal validation	Risk scoring system
Lü Yangyang 2022 (21)	0.870	/	/	/	/	Regression equation
Liao Liping 2022 (18)	0.944(0.922,0.965)	0.930(0.898,0.963)	/	Calibration curve; H-L test (*p* = 0.831 > 0.05)	Random split internal validation	Nomogram
Li Zhe 2021 (15)	0.914 (0.877, 0.943)	/	/	/	/	Regression equation
Eun Young Hur 2019 (12)	/	/	0.970	/	Random split internal validation; Temporal external validation	Risk scoring system

H-L test: Hosmer-Lemeshow test; External validation types differ across reports: Eun Young Hur 2019 conducted temporal external validation (successive time-period cohorts within the same institution); Sufriyana 2025 conducted geographic external validation (institutionally independent cohort). These two validation approaches assess different dimensions of model generalizability and their AUC values should not be interpreted as equivalent indicators of transportability.

### 3.5 Risk of bias and applicability assessment

All 13 reports were assessed using PROBAST across four domains: Participants (D1), Predictors (D2), Outcome (D3), and Analysis (D4). Domain-level risk-of-bias ratings varied: Sufriyana 2025 and Zhu Rui 2024 were rated low risk in D1; Sufriyana 2025 was additionally rated low risk in D2 and D4. All other reports were rated high risk across all four domains, reflecting study-specific methodological deficiencies rather than a single common flaw. A summary of domain-level judgments is presented in Table 4.

**Table 4:** PROBAST risk of bias and applicability assessments of included CAUTI risk prediction models.

Included report	Risk of bias				Applicability concerns			Overall	
	Participants	Predictors	Outcome	Analysis	Participants	Predictors	Outcome	Risk of bias	Applicability concerns
Liu Huiying 2025 (16)	H	H	H	H	H	L	H	H	H
Li Mengchao 2025 (20)	H	H	H	H	H	L	L	H	H
Sufriyana 2025 (5)	L	L	H	L	H	H	H	H	H
Zhu Rui 2024 (19)	L	H	H	H	H	L	H	H	H
Sun Qing 2024 (17)	H	H	H	H	H	L	L	H	H
Wang Fang 2023 (13)	H	H	H	H	H	L	L	H	H
Liu Yuting 2023 (11)	H	H	H	H	H	L	H	H	H
Li Yuping 2023 (6)	H	H	H	H	H	L	H	H	H
Xia Liyao 2022 (14)	H	H	H	H	H	L	L	H	H
Lü Yangyang 2022 (21)	H	H	H	H	H	L	L	H	H
Liao Liping 2022 (18)	H	H	H	H	H	L	L	H	H
Li Zhe 2021 (15)	H	H	H	H	H	L	L	H	H
Eun Young Hur 2019 (12)	H	H	H	H	H	L	L	H	H

Overall risk of bias was high for all 13 reports. Domain-level ratings varied: Sufriyana 2025 was rated low risk in Domains 1, 2, and 4; Zhu Rui 2024 was rated low risk in Domain 1; all other reports were rated high risk in all four domains. High-risk ratings reflect study-specific methodological deficiencies and were not assigned on the basis of retrospective design alone. Applicability concerns are reported separately and should not be conflated with risk of bias ratings. D1 = Participants; D2 = Predictors; D3 = Outcome; D4 = Analysis; H = high risk; L = low risk. Domain-level ratings of H reflect the presence of at least one signaling question rated N or PN within that domain; signaling questions rated U (unclear) are not shown separately but contributed to domain-level assessment where applicable.

#### 3.5.1 Participants domain

Eleven of the 13 reports were rated as high risk of bias in the Participants domain; Sufriyana 2025 and Zhu Rui 2024 were rated low risk.

For Sufriyana 2025, data were drawn from a large multi-institutional electronic health record database (TMUCRD) encompassing 122,386 catheterization episodes across three hospitals, with appropriate and transparent eligibility criteria and an exceptionally large sample size; all four signaling questions were satisfactory. For Zhu Rui 2024, 8,905 patients were enrolled from a geriatric ICU over 10 years with a clearly defined enrolment point; With EPV = 10.4, this report marginally met the commonly cited minimum threshold of 10, though it fell below the more contemporary recommendation of EPV ≥ 20; the cohort size was the most favorable participant-domain characteristic among all single-center reports.

Among the remaining 11 high-risk reports, the primary driver of concern was inadequate sample size relative to the number of candidate predictor parameters considered during model development — not retrospective design *per se*. Critically low EPV values were identified across most single-center reports: EPV = 1.4 in LI Yuping 2023 (17 training-set events, 12 candidate variables), EPV = 3.2 in LI Mengchao 2025 (32 events, 10 candidate variables), EPV = 4.6 in Wang Fang 2023 (88 events, 19 candidate variables), and EPV = 7.1 in LIU Huiying 2025 (57 training-set events, 8 candidate variables). The remaining single-center reports had EPV values ranging from approximately 5–16. In all cases, EPV was calculated against the number of candidate variables considered during model development rather than only those retained in the final model, as the latter systematically underestimates overfitting risk.

Two additional reports were rated high risk on grounds other than sample size. Hur Eun Young 2019 selected non-catheterized inpatients as controls, which is inappropriate for a model intended to predict CAUTI risk in catheterized patients. Sun Qing 2024 used a 1:1 matched case–control design that artificially inflated the event proportion to 50%, distorting the validity of absolute risk estimates despite an adequate absolute event count of 521.

#### 3.5.2 Predictors domain

Twelve of the 13 reports were rated as high risk of bias in the Predictors domain; Sufriyana 2025 was the sole exception, rated low risk.

The dominant concern across high-risk reports was temporal leakage: the systematic inclusion of time-varying predictors in models nominally intended for use at or near the time of catheter insertion or hospital admission. A predictor was classified as time-varying — and therefore subject to temporal leakage — when its value could only be determined after the prediction time point; this classification was supported by distributional evidence of post-event accumulation in CAUTI cases relative to controls.

Catheter dwell time was the most frequently affected predictor, retained as a time-varying variable in seven final models. In every case, distributional data confirmed post-event accumulation rather than a baseline characteristic: CAUTI cases consistently showed two- to fourfold higher dwell times than controls — for example, 21 days versus 7 days in LIU Huiying 2025, and catheter duration >30 days in 90.6% versus 31.5% of controls in LI Mengchao 2025; similar patterns were observed in ZHU Rui 2024 (75.4% vs. 19.7% with duration ≥10 days) and WANG Fang 2023 (64.8% vs. 24.3% with duration >28 days). Where regression coefficients were reported, catheter dwell time carried the largest weight among all final predictors: β = 1.57 (OR = 4.78) in XIA Liyao 2022 and β = 1.143 (OR = 3.135) in LÜ Yangyang 2022. In LIU Yuting 2023, it served as the primary splitting variable in a CRT decision tree (*p* = 0.001) despite the model being designated for admission-time use — a contradiction that exemplifies the broader leakage problem.

ICU or hospital length of stay appeared as a time-varying final predictor in LIAO Liping 2022, LI Zhe 2021, and ZHU Rui 2024. LIAO Liping 2022 exhibited the broadest temporal contamination among all single-center reports: two of its five final predictors — indwelling catheter duration ≥30 days (OR = 6.170) and ICU stay ≥14 days (OR = 5.255) — are both time-varying variables unknowable at admission and together account for 40% of the final model predictors, each carrying the largest effect estimates in the model. In LI Zhe 2021, both ICU admission and prophylactic antibiotic use were determined during hospitalization rather than at catheter insertion, with reverse causality additionally possible for ICU admission (whereby CAUTI itself may precipitate transfer to ICU). In Hur Eun Young 2019, total catheter duration and total length of hospital stay were end-of-stay cumulative values abstracted with full knowledge of outcome status; both are on the causal pathway to CAUTI rather than antecedent predictors, and the automated system’s default rules additionally incorporated urine white blood cell counts, bacteriuria on microscopy, and urine culture results — markers that are proximal indicators of the outcome itself.

The most severe instance of temporal leakage was LI Yuping 2023, in which the dominant predictor — length of neuro-ICU stay greater than 30 days (OR = 272.2, *β* = 5.067) — is defined over the identical observation window as CAUTI ascertainment; this represents an irresolvable overlap between predictor measurement and outcome determination rather than a time-varying predictor that is merely inconvenient to measure at baseline. In each affected report, the presence of time-varying predictors in the final model means the stated purpose of early or admission-time risk stratification cannot be fulfilled: a model cannot be applied at catheter insertion if its most influential predictor requires knowledge of the entire subsequent hospitalization period. The apparent discriminative performance of these models therefore reflects future information rather than genuine predictive signal.

For Sufriyana 2025, the use of time-varying predictors — including daily catheterization duration — was a deliberate design feature of a daily repeated-measures prediction framework rather than inadvertent temporal leakage; predictor values accumulated to the point of each daily prediction are genuinely available at that time point, and predictor measurement was therefore internally consistent with the model’s stated workflow. This differs fundamentally from the ten reports described above, where final-model predictors reflect cumulative values determinable only at the end of hospitalization, while the stated clinical application is admission-time or catheter-insertion-time prediction. The domain was accordingly rated low risk for Sufriyana 2025. Sun Qing 2024 was rated high risk in D2 because three of its four final predictors — duration of catheterization, length of ICU stay, and number of antibiotic types — are time-varying variables unavailable at catheter insertion; the temporal leakage is additionally captured under D3.5.

Retrospective predictor extraction was not rated as high risk unless there was specific evidence that predictor ascertainment could have been influenced by knowledge of outcome status. For XIA Liyao 2022, predictor data were retrospectively completed after catheter removal — meaning the researcher was aware of CAUTI status at the time of recording some predictor values — though most predictors are objective surgical or pathological characteristics unlikely to be systematically distorted. For Hur Eun Young 2019, total catheter duration and total length of stay were end-of-stay values abstracted with full knowledge of case status, and the system’s default rules incorporated outcome-adjacent urine microscopy and culture markers.

#### 3.5.3 Outcome domain

All 13 reports were rated as high risk of bias in the Outcome domain, arising from different combinations of concerns across signaling questions.

The most prevalent concern was concurrent measurement of key predictors and the outcome (D3.5), rated PN or N in ten reports, directly reflecting the temporal leakage identified in D2.3. The most severe instance was LI Yuping 2023 (D3.5 = N), in which the dominant predictor is defined over the same interval as CAUTI ascertainment. LIAO Liping 2022 had two final predictors — catheter duration ≥30 days and ICU stay ≥14 days — both measured concurrently with outcome ascertainment, representing the broadest dual temporal confounding in the series. Hur Eun Young 2019 similarly had D3.5 rated N, as total catheter duration and total hospital stay were finalized after CAUTI onset and are themselves on the causal pathway to infection.

Non-standard or incompletely specified outcome definitions represented a second major concern. Sufriyana 2025 used an administratively derived surrogate — a diagnostic code for UTI or a urine culture order without concurrent pneumonia — that does not correspond to any recognized NHSN/CDC, IDSA 2009, or ESCMID criterion; D3.2 was rated N. LIU Yuting 2023 and LI Yuping 2023 both cited the NHSN/CDC definition but applied only the microbiological component (urine culture ≥10^5^ CFU/mL), omitting the required clinical symptom criterion; the authors of LIU Yuting 2023 explicitly acknowledged this as a potential source of overdiagnosis. Zhu Rui 2024 included catheter-associated asymptomatic bacteriuria alongside symptomatic CAUTI, conflating two clinically distinct entities that are explicitly separated in international standard definitions. Wang Fang 2023, by contrast, applied the most complete and guideline-concordant definition among all included reports — clinical symptoms plus ≥10^3^ CFU/mL per IDSA 2009 — and was rated PY for D3.2.

Critically small absolute event counts were rated N for D3.6 in LIU Yuting 2023 (21 total events; only 5 in the high-risk stratum), LI Yuping 2023 (17 training-set events, 4 validation-set events), and LI Mengchao 2025 (32 events), rendering all performance estimates for these three reports highly unstable.

Outcome blinding (D3.4) could not be confirmed in any retrospective report and was rated U throughout; this is an inherent feature of retrospective electronic record studies and was not elevated to a domain-level concern in isolation.

#### 3.5.4 Analysis domain

Twelve of the 13 reports were rated as high risk of bias in the Analysis domain; Sufriyana 2025 was rated low risk.

The most prevalent analytical concern was univariable pre-screening followed by multivariable modelling (D4.5 = N), identified in nine reports: LIU Huiying 2025, LI Mengchao 2025, Zhu Rui 2024, LI Yuping 2023, XIA Liyao 2022, LÜ Yangyang 2022, LIAO Liping 2022, LI Zhe 2021, and Hur Eun Young 2019. This two-stage approach — selecting predictors based on univariable statistical significance before fitting the multivariable model — is a recognized source of model overfitting, selection instability, and inflated performance estimates. The *P*-value thresholds used for screening varied across reports (*p* < 0.05 in XIA Liyao 2022; *p* < 0.25 in LI Yuping 2023; *p* < 0.2 in LI Zhe 2021), but all represent data-driven selection from the same dataset used for model development. Hur Eun Young 2019 applied the most extreme form of data-driven selection, filtering from an initial candidate pool of 4,200 variables using weight-of-evidence and information value methods followed by stepwise logistic regression. Four reports avoided this problem: Wang Fang 2023 used LASSO regression; LIU Yuting 2023 entered all 16 candidate variables directly into the decision tree without prior screening; Sun Qing 2024 (17) applied conditional logistic regression with a pragmatic two-threshold approach; and Sufriyana 2025 used structural causal modelling with Cox proportional-hazards regression and Benjamini-Hochberg multiple testing correction.

Inappropriate handling of continuous predictors (D4.2 = PN or N) was identified in ten reports. Dichotomization of continuous variables — applied to age, catheter duration, albumin, GCS, and others — was the predominant approach, discarding statistical information and producing threshold instability. No report other than Sufriyana 2025 assessed linearity of continuous predictors on the log-odds scale. LI Mengchao 2025 additionally dichotomized ordinal variables including ASIA score and number of catheterizations without justification.

Missing data were handled by complete-case exclusion in all 13 reports, without description of the missing data mechanism, the number of affected patients, or any sensitivity analysis. The extent of exclusions ranged from 7 patients to 47 patients among those that reported a number at all; most reports did not quantify exclusions.

External validation was absent in 11 reports. Only Sufriyana 2025 performed genuine external validation using data from an independent hospital. Hur Eun Young 2019 used two temporal validation cohorts from the same institution, which provides some reassurance of temporal stability but does not constitute multi-center external validation. The remaining 11 reports relied on random split-sample validation (6, 14, 18), bootstrap internal validation (13, 16, 17, 19, 20), or no validation at all (11, 15, 21).

Calibration assessment was incomplete across all 13 reports. A calibration curve was presented in eight of the 13 reports, but quantitative calibration metrics were inconsistently reported: the Hosmer-Lemeshow test was reported in LI Mengchao 2025, Zhu Rui 2024, and LIAO Liping 2022; Brier score in LIU Yuting 2023; and calibration curves and Brier score in Sufriyana 2025. XIA Liyao 2022 reported no calibration assessment of any kind. Decision curve analysis was provided in LIU Huiying 2025, Sun Qing 2024, Wang Fang 2023, Sufriyana 2025, and LI Yuping 2023.

The derivation AUC of 0.993 reported by LI Mengchao 2025 — from a model with EPV = 3.2 and data-driven variable selection — almost certainly reflects severe overfitting; an AUC approaching unity under such constraints is implausible and should not be taken at face value. Sufriyana 2025 was the only report rated low risk in D4: it employed structural causal modelling for predictor selection, applied five machine-learning algorithms with regularization, conducted comprehensive calibration assessment including slope, intercept, and Brier score, and validated performance on a genuinely independent external test set.

#### 3.5.5 Applicability assessment

All 13 reports raised applicability concerns in at least one domain; these should be distinguished from risk of bias judgments.

Applicability concerns in the Participants domain were rated high for all 13 reports. For most single-center Chinese reports, the primary reason was restriction to highly specific patient populations: stroke inpatients (16), spinal cord injury surgical patients (20), NICU patients (6, 11, 15), traumatic brain injury patients (18), older critically ill patients with APACHE II ≥ 25 (19), post-radical hysterectomy cervical cancer patients (13), gynecological malignancy patients (14), and emergency department patients (21). For Sufriyana 2025, the concern arose from restriction to an ethnic Chinese Taiwanese population aged 20–75 years, excluding patients over 75 — a group at particularly high risk of CAUTI. For Hur Eun Young 2019, the case–control design with non-catheterized controls does not reflect the target population for CAUTI prediction in catheterized patients.

Applicability concerns in the Predictors domain were rated low for 12 of the 13 reports. Sufriyana 2025 was rated high concern in this domain because several of its predictors — including HIV infection status, diabetic nephropathy coding, and system-derived catheterization indication codes — depend on specific administrative coding practices of the Taiwanese TMUCRD database and may not be consistently available or equivalently defined in other institutional electronic health record systems. The remaining 12 reports relied on standard demographic, comorbidity, laboratory, or routine nursing variables available in clinical practice without proprietary instruments.

Applicability concerns in the Outcome domain varied. Reports applying the 2010 Chinese Ministry of Health guideline with both symptom and microbiological criteria were rated low concern (14, 15, 17, 18, 21); Wang Fang 2023, which used the IDSA 2009 definition, and LI Mengchao 2025, which applied a standard postoperative infection definition, were similarly rated low concern. Hur Eun Young 2019 was also rated low concern in the Outcome domain, as the CAUTI definition itself was standard; the applicability concern for this report resides in the Participants domain rather than the outcome definition. Reports using non-standard or partial definitions were rated high: Sufriyana 2025 (administratively derived definition), LIU Yuting 2023 and LI Yuping 2023 (microbiological criterion only), and Zhu Rui 2024 (CAASB included in the outcome group). LIU Huiying 2025 was rated high concern due to incompletely specified diagnostic criteria.

## 4 Discussion

### 4.1 Methodological limitations undermine confidence in reported model performance

High derivation AUC values — nine of 13 exceeded 0.800 — are misleading absent external validation: small samples and data-driven variable selection both inflate apparent performance. PROBAST assessment confirmed this concern, rating all 13 reports at high overall risk of bias, driven principally by deficiencies in the Outcome and Analysis domains. Only two reports conducted external validation, and the available evidence of generalizability is therefore limited.

With respect to study design, all 13 included reports employed a retrospective design. Retrospective studies rely on existing medical records, and systematic exclusion of patients with incomplete documentation narrows the case-mix the model must distinguish, potentially inflating apparent discriminative performance.

A separate and more fundamental concern relates to the intended prediction time point, which varied substantially across reports — from a single assessment at catheter insertion or hospital admission to a daily repeated-measures framework updated throughout the catheterization episode. This heterogeneity is clinically important: models operating at different prediction time points address distinct clinical questions, and their performance metrics are not directly comparable. Among the 12 reports describing admission-time or catheter-insertion-time models, 10 incorporated time-varying predictors — variables unavailable at the intended prediction time point — representing the most pervasive concern in this domain. In these 10 reports, predictors such as total catheter dwell time, total length of hospital or ICU stay, and cumulative exposure variables were incorporated into models stated to be for use at catheter insertion or hospital admission — yet these values are unavailable at that moment. Sufriyana 2025 was the sole exception: its daily repeated-measures framework rendered time-varying predictors methodologically appropriate, as values accumulated up to each daily prediction are genuinely available when that prediction is made. A model that incorporates information generated after the prediction time point cannot be applied prospectively; its apparent discriminative performance therefore reflects future information rather than genuine predictive signal, and the model cannot function as a clinical prediction tool regardless of its reported AUC. Post-event accumulation was quantitatively substantial: catheter dwell time at CAUTI diagnosis was 2–4 times higher in cases than controls across multiple reports, and the most severely affected predictor — neuro-ICU length of stay in LI Yuping 2023 — carried an odds ratio of 272.2, reflecting near-complete co-determination of predictor and outcome rather than any genuine predictive relationship. These effect sizes should not be interpreted as evidence of strong clinical signal; they are artefacts of temporal confounding.

Beyond study design, the included reports exhibited methodological deficiencies across all four PROBAST domains. The most consequential are discussed below.

Insufficient sample size relative to model complexity was the most consequential analytical deficiency identified. EPV-based thresholds apply specifically to standard logistic regression: PROBAST recommends a minimum EPV of 20 for such models (10), yet the majority of logistic regression reports in this review failed to meet this threshold, with EPV values as low as 1.4. When EPV is inadequate, regression coefficients tend to be inflated and calibration poor, and reported AUC values are prone to optimism (22). For the decision tree report (11), EPV thresholds are not directly applicable; with only 21 outcome events, terminal node estimates are inherently unstable regardless of the EPV metric, indicating severe underpowering. For the machine learning report (5), sample size adequacy is better demonstrated by the large absolute sample, built-in regularization, and independent external validation than by EPV alone. Future studies should address these limitations through penalized regression (e.g., Firth’s or ridge regression) and *a priori* sample size calculations appropriate to the chosen modelling method (23, 24).

Dichotomization of continuous predictors represented another important analytical deficiency. Evidence indicates that dichotomizing a continuous predictor with a linear relationship to the outcome results in an approximately 37% loss of statistical power; when the true relationship is nonlinear, dichotomization may additionally introduce systematic bias through misspecification of the predictor–outcome functional form (25). In this review, 11 reports applied dichotomization to predictors including age, serum albumin level, and catheter dwell time (5, 6, 11–15, 17–19, 21). Furthermore, cut-off values for the same predictor differed substantially across reports — for instance, age cut-offs ranged from 60 to 75 years — rendering direct cross-study comparison of effect sizes infeasible. Future studies should retain the continuous nature of predictors by modelling nonlinear relationships using restricted cubic splines or fractional polynomials (26). Where categorization is considered necessary for clinical use, cut-points should be prespecified on clinical or biological grounds. Data-driven cut-point selection is prone to instability and optimism; any threshold derived from the development dataset requires confirmation in an independent validation cohort before clinical adoption (23).

Missing data handling was uniformly inadequate across the included reports. Two reports excluded participants with missing data entirely (6, 17); Sufriyana 2025 addressed missing data at the predictor selection stage by excluding predictors with missing-not-at-random patterns rather than excluding participants (5), while the remaining 10 neither reported the extent of missing data nor described the approach used to handle it (11–16, 18–21). Excluding participants with missing values yields unbiased estimates only when data are missing completely at random (MCAR); when data are missing at random (MAR) or missing not at random (MNAR), this approach introduces selection bias and reduces statistical efficiency. Failure to report missing data handling procedures undermines the transparency and reproducibility of the findings (27). Future studies should adopt multiple imputation as the preferred strategy for handling missing data (10) and report missing data procedures in full accordance with the TRIPOD+AI statement.

Variable selection approaches varied but were predominantly data-driven: nine reports used univariable or data-driven pre-screening as the primary method of predictor selection (6, 12, 14–16, 18–21), an approach that fails to account for interaction effects among predictors and produces unstable selection when sample sizes are limited. One report applied conditional logistic regression with a pragmatic two-threshold screening approach (17). One report adopted LASSO regression (13), which simultaneously addresses multicollinearity and reduces the risk of overfitting through coefficient penalization (28). One report employed structural causal modelling with Benjamini-Hochberg multiple testing correction for predictor selection (5), an approach that explicitly addresses confounding and multiple testing while prespecifying the causal structure underlying predictor selection. One report used decision tree-based variable selection without prior univariable screening (11). Future studies should specify candidate predictors *a priori* on the basis of existing evidence and clinical expertise. When penalization is warranted by sample size and predictor count, LASSO, ridge regression, or elastic net are all reasonable options; the choice should be guided by the modelling context (29).

Model performance reporting was incomplete in the majority of included reports. Only three reports used both calibration curves and the Hosmer-Lemeshow test (18–20). Discrimination and calibration represent complementary and independent dimensions of model performance: a model may achieve a high AUC while exhibiting poor calibration, rendering it clinically unreliable despite apparently strong discriminative ability. Furthermore, the Hosmer-Lemeshow test is sensitive to sample size — yielding spuriously non-significant results in small samples and spuriously significant results in large ones — and conclusions drawn from this test alone are therefore insufficient to characterize model calibration (30). Future studies should report a comprehensive suite of performance metrics, including calibration curves, calibration slope, and the Brier score, in accordance with TRIPOD+AI reporting recommendations.

External validation — the most critical determinant of a model’s clinical applicability — was conducted in only two reports (5, 12), and three reports performed no validation of any kind (11, 15, 21). This gap is the most consequential limitation of the current evidence base. Temporal, geographic, and domain-based validation are essential for distinguishing genuinely generalizable models from overfit ones (31); without external validation, high AUC values cannot be taken as evidence of true predictive ability. Of the two reports that performed external validation, one used temporal validation (AUC = 0.970) (12) and the other used geographic validation (5); in the latter, the best-performing model achieved an AUC of 0.758 in the external cohort. This drop in performance underscores that internal validation results are insufficient to predict how a model will perform in a new clinical setting.

Taken together, existing CAUTI risk prediction models exhibit systematic methodological limitations spanning study design, analytical approach, and reporting practice — including pervasive reliance on retrospective designs, insufficient EPV, inappropriate handling of continuous predictors, and near-complete absence of external validation. Additionally, given that models with poor predictive performance are less likely to be published, this systematic review may itself be susceptible to publication bias, which would further inflate the apparent performance of the published literature. Until these limitations are adequately addressed and robust external validation evidence becomes available, the evidence base for direct clinical application of existing CAUTI risk prediction models remains insufficient.

### 4.2 Differences and commonalities in predictor selection

The number of predictors included in final models ranged from 4 to 14. Despite heterogeneity in study populations, notable overlap was observed in predictor selection across models, with the five most frequently identified predictors being age, catheter dwell time, level of consciousness, diabetes mellitus, and serum albumin level. Across included models, predictor selection reflects variable availability in retrospective records, prevailing clinical assumptions, and analytical convention. Recurrence of a predictor across independently developed models does not validate it as a reliable predictor of CAUTI, nor does it establish causality. Where inclusion of a predictor is nevertheless recommended below, this recommendation rests on its independent biological plausibility and supporting evidence from studies outside this review — not on its frequency of model inclusion.

Age is a well-established CAUTI risk factor with a plausible biological basis. Immunosenescence progressively impairs host defense against uropathogenic organisms, and older catheterized patients consistently demonstrate higher CAUTI incidence than younger counterparts (31). Additionally, diminished bladder contractility in older patients increases post-void residual volume, promoting urinary stasis and creating a microenvironment conducive to bacterial colonization (31). These biological mechanisms are consistent with the clinical literature, and existing catheter management guidelines already recommend heightened attention in older patients (31). The frequency with which age was selected as a predictor across the included models is consistent with this external evidence base, though it does not independently validate it. Such inferences require evidence from appropriately designed causal and interventional studies, which falls outside the scope of this review. The discussion of biological mechanisms below is therefore intended to contextualize associations, not to endorse clinical interventions.

Catheter dwell time was consistently identified as a predictor of CAUTI across the included models. A case–control study of 7,295 patients demonstrated that a dwell time of ≥10 days constitutes an independent risk factor for CAUTI (32), and infection risk increases progressively with dwell time (33). The biological mechanisms underlying this association are well characterized: biofilm accumulates on the catheter surface over time, creating a microenvironment conducive to bacterial adhesion and proliferation, while the catheter’s continuous connection between the urinary tract and the external environment disrupts the natural urethral barrier, elevating infection risk (34). Furthermore, prolonged hospitalization is generally accompanied by a corresponding increase in catheter dwell time, during which extended exposure of the urethral mucosa to mechanical irritation and nosocomial drug-resistant organisms further compounds infection risk (32, 33). Established infection prevention guidelines recommend regular reassessment of catheter necessity and prompt removal once clinical indication has ceased (3), a recommendation supported by independent observational evidence (32, 33); the consistent identification of catheter dwell time as a predictor across the included models is consistent with this external evidence base but does not of itself add to it. Nevertheless, this consistency supports its *a priori* inclusion as a candidate predictor in future model development, provided that measurement is prospectively defined at fixed time points to avoid temporal leakage.

Impaired consciousness was another consistently identified predictor, with a plausible mechanistic basis. Reduced mobility and diminished self-care capacity in this patient group compromise perineal hygiene and elevate the risk of bacterial colonization (35). Impaired consciousness is also frequently associated with prolonged bed rest, urinary retention, and reduced immune responsiveness, all of which are recognized risk factors for nosocomial infection. Patients with reduced consciousness are therefore plausibly at elevated risk, and this is reflected in their consistent identification as a high-risk group across independent observational studies (35). Whether impaired consciousness adds independent predictive value beyond severity markers such as APACHE II or GCS score — which capture overlapping clinical information — was not systematically examined in the included reports. Future studies should formally assess collinearity among consciousness-related severity markers — for instance, by examining variance inflation factors — prior to variable selection, to avoid including redundant predictors that inflate apparent model complexity without improving predictive accuracy.

Diabetes mellitus was also frequently identified as a predictor of CAUTI, consistent with established evidence of a substantially elevated infection risk in this patient population (36). The primary mechanism involves sustained hyperglycemia, which impairs urothelial integrity and accelerates bacterial adhesion and colonization; glucosuria further provides a nutrient-rich substrate for pathogen growth (37, 38). Diabetes mellitus is additionally associated with immune dysregulation — including impaired neutrophil chemotaxis, reduced phagocytic function, and diminished cytokine responsiveness — together impairing the host’s capacity to clear uropathogenic organisms (39). In clinical practice, diabetes mellitus is already recognized as a risk factor for nosocomial infections more broadly, and existing evidence supports careful glycemic management in hospitalized patients on general patient safety grounds (36, 39). Diabetes mellitus was consistently identified as a correlate of CAUTI risk across the included models. Its consistent identification across studies with different populations supports its inclusion as a candidate predictor in future models.

Serum albumin level, an established marker of nutritional status, was identified as a predictor of CAUTI in multiple included models. Hypoalbuminemia impairs immune function through several concurrent mechanisms: it reduces neutrophil function and complement activity, undermining innate immune defenses against uropathogenic organisms, and compromises tissue repair capacity, thereby increasing susceptibility to nosocomial infections including CAUTI (40). Serum albumin is nevertheless susceptible to non-nutritional influences — including acute-phase responses, hepatic dysfunction, and fluid shifts — which may confound its interpretation as a nutritional marker in critically ill patients, and this limits the specificity of hypoalbuminemia as a predictor of infection risk (40). Its appearance in multiple prediction models is therefore consistent with a role as a marker of overall physiological reserve rather than nutritional status per se. Despite its physiological limitations as a marker in critically ill patients, serum albumin is readily available and its inclusion as a candidate predictor is reasonable.

### 4.3 Comparative analysis of modeling approaches

The 18 prediction models included in this review employed three distinct modelling approaches: logistic regression, machine learning algorithms, and decision trees. These approaches differ substantially in interpretability, predictive performance, sample size requirements, and clinical applicability.

#### 4.3.1 Logistic regression models

Logistic regression dominated, used in 11 reports (6, 12–21). Its advantages — interpretable coefficients, straightforward operationalization as nomograms or scoring systems, and reliable performance at moderate sample sizes — make it well suited to clinical prediction contexts (41). Its limitations are equally well characterized: the linearity assumption may misspecify nonlinear predictor–outcome relationships, and it is prone to overfitting when EPV is inadequate (42). Among the logistic regression models included in this review, derivation AUC values ranged from 0.677 to 0.993, reflecting considerable heterogeneity in apparent discriminative performance; these figures reflect performance on the development data and should not be interpreted as estimates of true predictive ability in independent populations.

Logistic regression nevertheless has inherent methodological limitations. It assumes a linear relationship between each predictor and the log-odds of the outcome; for predictors with nonlinear associations — such as age, serum albumin level, and catheter dwell time — this assumption may result in misspecification of the predictor–outcome functional form. It is also susceptible to multicollinearity, which can destabilize coefficient estimates when predictors are strongly intercorrelated, and under conditions of insufficient EPV, prone to overfitting that compromises generalizability to external populations (42).

#### 4.3.2 Machine learning methods

One report compared five approaches directly: ridge regression, decision tree, random forest (RF), XGBoost, and the deep learning-based DI-VNN (5). In the development cohort, DT, RF, and XGBoost achieved AUC values of 0.788, 0.788, and 0.787, respectively, all outperforming ridge regression (AUC = 0.752) and DI-VNN (AUC = 0.611). As these metrics reflect performance on the development data rather than an independent sample, they are susceptible to optimism and cannot be taken as reliable indicators of generalizability; whether this performance advantage is reproducible in external populations remains to be established. The principal strengths of machine learning approaches include the capacity to automatically capture nonlinear predictor relationships and interaction effects, robustness to outliers and missing data, and theoretically superior predictive performance in large, high-dimensional datasets (43).

Machine learning methods nevertheless have notable limitations in clinical prediction modelling. The most consequential is interpretability: the internal processes of ensemble models such as random forest and XGBoost cannot be readily translated into clinically actionable decision rules, limiting their utility at the point of care even when predictive performance is acceptable. These methods also impose substantial sample size requirements and are prone to overfitting on small clinical datasets — a concern not allayed by the fact that the sole included report relying on machine learning evaluated performance through internal validation alone. Hyperparameter optimization adds further analytical complexity that does not consistently translate into improved performance over logistic regression in clinical settings, and the opacity of machine learning pipelines complicates both replication and external validation (44).

#### 4.3.3 Decision tree models

One report (11) applied two decision tree algorithms — CHAID and CRT — which achieved development AUC values of 0.852 and 0.920, respectively; as discussed below, these figures are likely to overestimate true predictive performance given the critically low EPV of 1.3. Decision tree approaches offer several advantages. The resulting tree structure is intuitive and readily interpretable, allowing clinicians to follow the branching rules directly at the point of care without requiring additional computational tools. Decision trees automatically capture interaction effects without assuming linear relationships and accommodate both continuous and categorical predictors without distributional assumptions.

However, decision tree methods have pronounced limitations. They are sensitive to small perturbations in the training data, resulting in high variance and limited reproducibility across datasets. Overly deep decision trees tend to produce complex rule systems, limiting their clinical interpretability. Furthermore, the evidence base for decision tree methods in CAUTI prediction remains insufficient, with only one report identified in this review. That report had only 21 outcome events, grossly insufficient to populate the terminal nodes of a multi-level decision tree with stable probability estimates, and the reported AUC values of 0.852 and 0.920 are likely substantially optimistic (22).

#### 4.3.4 Comparison of model presentation formats

Beyond the choice of modelling algorithm, the presentation format of a prediction model has important implications for clinical utility. Among the models included in this review, the nomogram was the most widely used format, employed in eight reports. Nomograms enable intuitive visualization of each predictor’s contribution to individual risk, making them well suited for individualized risk assessment at the point of care, though their use typically requires a printed chart or digital tool (41). Scoring systems, used in three reports, are the most straightforward to implement and appropriate for rapid clinical screening, at the cost of reduced predictive precision. Regression equations, used in two reports, offer the most comprehensive predictive information but are the least feasible for routine clinical use given their dependence on computational tools.

In summary, logistic regression, machine learning, and decision tree approaches each carry distinct tradeoffs in interpretability, handling of nonlinear relationships, sample size requirements, and point-of-care feasibility. Logistic regression offers the most favorable balance across these dimensions given the sample sizes and clinical workflows typical of CAUTI prediction contexts. Machine learning methods offer theoretical advantages in predictive performance when data are abundant, but at substantial cost to interpretability and methodological accessibility. Decision tree methods are distinctive in their visual interpretability and freedom from distributional assumptions, but raise concerns about structural instability and limited reproducibility across datasets. The choice among these approaches should therefore be guided by the available sample size, the priority placed on clinical interpretability, and the intended use case.

### 4.4 CAUTI risk prediction models are still in the developmental stage

Overall, research on CAUTI risk prediction models remains at an early stage, and the evidence base is both sparse and methodologically immature. The concentration of included reports in Asian clinical settings — predominantly mainland China, with additional contributions from Taiwan and South Korea — further constrains the generalizability of current findings. Given systematic regional differences in patient demographics, catheter management practices, and infection control standards, the transferability of existing models to non-Asian settings cannot be assumed and warrants empirical evaluation in geographically diverse cohorts.

Beyond these regional constraints, the methodological deficiencies documented above are not isolated shortcomings but reflect the broader developmental stage of CAUTI risk prediction research as a whole. Performance evaluation has focused predominantly on discrimination while calibration has been largely overlooked — a pattern that, combined with near-universal absence of external validation, means the current evidence base cannot support reliable conclusions about any existing model’s readiness for clinical deployment.

The evidence base across modelling strategies is also markedly uneven. Logistic regression was applied in 11 of the 13 included reports, while machine learning algorithms and decision tree methods were each employed in only one (5, 11) — a distributional imbalance that precludes evidence-based conclusions about the relative merits of different approaches in this clinical context. Whether the theoretical performance advantages of machine learning or the interpretive accessibility of decision tree structures can be realized in CAUTI prediction remains to be established through adequately powered, head-to-head comparative studies.

On the basis of these findings, several directions merit particular attention. First, given the substantial number of CAUTI prediction models already in the literature, future efforts should prioritize multicenter, temporal, and cross-population external validation of the most methodologically sound existing models, with the explicit goal of establishing whether any can demonstrate sufficient transportability to justify clinical deployment, rather than continuing to generate novel models without such evidence. Second, where new model development is undertaken, the intended prediction time point should be explicitly prespecified and consistently applied; predictor assessment windows should be defined *a priori*, with a clear distinction between variables available at the moment of prediction and those determined only after follow-up begins. Where prospective data collection is not feasible and retrospective sources are used, the temporal sequencing of all predictors relative to the outcome must be explicitly documented and verified to preclude information leakage. Third, model development and reporting should adhere fully to the TRIPOD+AI statement and address all four domains assessed by PROBAST, with sufficient methodological detail to permit independent replication and critical appraisal. Fourth, performance evaluation should encompass both discrimination and calibration as standard, with calibration curves, calibration slope, and the Brier score reported alongside AUC. Fifth, where comparisons of modelling approaches are undertaken, machine learning algorithms may be explored provided that sample sizes are adequate, both internal and external validation are conducted, and interpretability-enhancing methods are employed to support translation into clinical practice.

These priorities point toward a single overarching need: to consolidate rather than proliferate, and to validate rather than develop. In the context of clinical prediction, the utility of any model is ultimately determined not by its derivation performance but by its demonstrated generalizability across independent populations; until existing CAUTI models have been subjected to prospective external validation in diverse clinical settings, the continued development of novel models without validation evidence risks compounding the current evidence gap rather than resolving it.

### 4.5 Limitations

Several limitations of this study should be acknowledged.

1. Lack of prospective registration: This review was not prospectively registered on PROSPERO or any other platform, which limits the verifiability of our analytical decisions. To reduce *post hoc* bias, the research question, eligibility criteria, and data extraction framework were prespecified before data collection.
2. Language restriction: Only studies published in Chinese or English were included, potentially excluding relevant studies published in other languages and limiting the comprehensiveness of the evidence base.
3. Potential publication bias: Formal assessment methods such as funnel plot asymmetry analysis were not feasible given the small number of included reports and the absence of a common quantitative metric suitable for pooling. Publication bias cannot be excluded; models with poor performance are less likely to be published, and the included reports may overestimate true predictive performance.
4. Inability to conduct quantitative synthesis: The included reports exhibited substantial heterogeneity across multiple dimensions — including study populations, modelling approaches, predictor sets, outcome definitions, and validation strategies — precluding both quantitative synthesis and direct cross-report performance comparisons.
5. Limited geographic representativeness: The vast majority of included reports were conducted in mainland China, Hong Kong, and Taiwan, with minimal representation from other regions. The generalizability of findings to clinical settings outside Asia cannot be assumed without further validation.
6. High risk of bias in primary studies: All 13 reports were rated high overall risk of bias by PROBAST, which substantially limits the confidence that can be placed in their reported performance estimates and constrains the strength of conclusions that can be drawn from this review.
7. Scarce external validation evidence: Only two reports (15.4%) performed external validation; the generalizability of the models described in the remaining 11 reports remains unassessed in independent datasets. The externally validated models achieved AUC values ranging from 0.758 to 0.970, indicating considerable uncertainty regarding model transportability across settings.
8. Limited population applicability: All 13 reports (100%) were rated as having high overall applicability concerns in the PROBAST assessment, driven primarily by study populations concentrated in specific clinical subgroups such as neurosurgical intensive care patients and postoperative gynecological oncology patients. This pattern reflects the high-concern rating in the Participants applicability domain across all 13 reports. Generalizable prediction models applicable to the broader population of hospitalized patients with indwelling urinary catheters are currently lacking.

## 5 Conclusion

This review identified 13 reports describing 18 CAUTI risk prediction models from 12 cohorts. Derivation AUC values ranged from 0.611 to 0.993, but external validation was conducted in only two reports, calibration was systematically underreported, and all 13 were rated high overall risk of bias by PROBAST. Temporal leakage — the incorporation of time-varying predictors into nominally admission-time models — was the most pervasive single deficiency, affecting 10 of 12 such models. No existing model has sufficient external validation evidence to support clinical implementation. Research should shift from model development toward multicenter external validation; where new models are built, prospective design, *a priori* predictor specification, adequate sample sizing, and full TRIPOD + AI-compliant reporting are required.

## Data Availability

Publicly available datasets were analyzed in this study. All data analyzed in this study were extracted from previously published peer-reviewed articles, whose full citations are listed in the References section. The extracted summary data are available from the corresponding author upon reasonable request.
