# Supplementary material for: Systematic review of risk prediction models for catheter-associated urinary tract infection
Source: Front Public Health. 2026 Sep 30;14:1884134. doi: 10.3389/fpubh.2026.1884134 (PMC13662166; doi:10.3389/fpubh.2026.1884134)
Supplement: Supplementary file 1 [file Supplementary_file_1.DOCX]

Prespecified Review Protocol

Systematic Review of Risk Prediction Models for Catheter-Associated Urinary Tract Infection

**1. Background and Rationale**

Catheter-associated urinary tract infection (CAUTI) is among the most prevalent healthcare-associated infections, accounting for 70–80% of hospital-acquired urinary tract infections. Prevention relies on standardised catheter management, yet individual risk varies substantially even under comparable catheterisation conditions. Clinical prediction models offer a structured approach to early identification of high-risk individuals and targeted intervention. A growing number of CAUTI risk prediction models have been published in recent years, but their methodological quality and clinical applicability have not been systematically appraised using established frameworks for prediction model reviews. This review synthesises the available evidence and identifies methodological priorities for future model validation and development.

**2. Objectives**

The primary objective was to identify and characterise all published CAUTI risk prediction models for adult inpatients, to appraise their methodological quality and risk of bias, and to summarise their predictive performance. Secondary objectives were to describe patterns of predictor selection across models, to identify methodological limitations in the existing evidence base, and to offer recommendations for future model development and validation.

**3. Research Question (PICOTS Framework)**

Population (P): Adult inpatients aged ≥18 years with an indwelling urinary catheter

Index model (I): Development, validation, or updating of a multivariable CAUTI risk prediction model

Comparator (C): None

Outcome (O): Occurrence of catheter-associated urinary tract infection

Timing (T): During catheterisation or within 48 hours of catheter removal

Setting (S): Acute care hospital settings, including ICUs, general wards, and surgical wards

**4. Eligibility Criteria**

Inclusion criteria

- Studies enrolling adult inpatients aged ≥18 years with an indwelling urinary catheter
- Development, validation, or updating of a formal multivariable CAUTI risk prediction model
- Cohort, case-control, or cross-sectional study design
- Published in Chinese or English

Exclusion criteria

- Studies identifying CAUTI risk factors without constructing a formal multivariable prediction model
- Conference abstracts, studies without accessible full text, or studies with insufficient data for extraction
- Reports derived from the same cohort using identical modelling approaches without independent methodological contribution. Where multiple reports from the same cohort used distinct modelling approaches, all were retained as separate model reports but counted as a single cohort in population-level analyses

**5. Literature Search**

Eight electronic databases were searched from inception to the search date: PubMed, Embase, Web of Science, Cochrane Library, CNKI, Wanfang Data, VIP, and CBM/SinoMed. Search strategies combined controlled vocabulary with free-text terms covering disease concepts (CAUTI and related terms) and modelling concepts (prediction model, risk score, nomogram, and related terms), applied in English and Chinese as appropriate. Full reproducible search strategies for all eight databases are provided in Supplementary Material S2.

**6. Study Selection**

Two reviewers independently screened all retrieved records at the title-and-abstract stage and subsequently at the full-text stage. Disagreements were resolved through discussion or by consulting a third investigator. The selection process and reasons for full-text exclusion are documented in accordance with PRISMA guidance and reported in Supplementary Material S3.

**7. Data Extraction**

Structured data extraction was performed independently by the same two reviewers using a prespecified form based on the CHARMS checklist. Extracted items included:

- Study identifiers: first author, publication year, country, data source
- Participants: study design, setting, eligibility criteria, sample size, number of outcome events, cohort partitioning (development, internal validation, external validation)
- Outcome: CAUTI definition and diagnostic criteria, ascertainment method
- Predictors: number and identity of candidate and final model predictors, variable handling (continuous versus categorised)
- Modelling approach: statistical method, variable selection strategy, missing data handling
- Prediction time point: intended time of model use, start and end of prediction horizon, whether all predictors would be available at that time point, and whether any predictor could have been influenced by the developing outcome
- Model performance: AUC with 95% confidence intervals, calibration statistics (calibration curve, Hosmer-Lemeshow test, calibration slope, intercept, Brier score), decision curve analysis, reported by evaluation type (derivation, internal validation, external validation)
- Model presentation format: nomogram, scoring system, regression equation, or other

All numerical values were independently verified by both reviewers against the original publications. Discrepancies were resolved by returning to the source document and, where necessary, by consulting the third investigator. Values not reported in the original publication were recorded as “not reported”.

To identify potentially overlapping study populations, all included reports were systematically cross-checked by comparing institution name, study period, sample size, patient population, and number of outcome events. Where two or more reports shared the same institution, similar sample size, and overlapping study period, the original publications were obtained and compared in detail to determine whether they originated from the same or independent cohorts.

The complete CHARMS extraction form is provided in Supplementary Material S6.

**8. Risk of Bias and Applicability Assessment**

Risk of bias and applicability were assessed independently by two reviewers using PROBAST across four domains: Participants (D1), Predictors (D2), Outcome (D3), and Analysis (D4). Assessment was conducted at the report level; for reports describing multiple models, a single domain-level judgment was assigned to the report. Disagreements were resolved through discussion or consultation with a third investigator.

Domain-level judgments were based on responses to individual PROBAST signalling questions and were not determined by study design alone. In particular, Domain-level judgments reflected the specific methodological features of each report as documented in the signalling question responses; design type alone was not used as the basis for any domain rating; instead, judgments reflected the specific methodological features of each report, as described in the signalling question responses. Applicability concerns were assessed and reported separately from risk of bias. Full signalling question responses and domain-level justifications for all 13 included reports are provided in Supplementary Material S5.

**9. Statistical Analysis**

Given anticipated heterogeneity across study populations, modelling approaches, outcome definitions, prediction time points, and validation strategies, quantitative synthesis of model performance was not planned a priori. Descriptive analyses were prespecified to summarise study characteristics, predictor frequencies, sample size adequacy, validation approaches, and model performance stratified by evaluation type (derivation, internal validation, and external validation). For logistic regression models, sample size adequacy was assessed using events per variable (EPV), with the number of candidate predictor parameters as the denominator rather than the number of predictors retained in the final model; method-appropriate criteria were applied to other modelling approaches.

**10. Reporting**

This systematic review was conducted and reported in accordance with the TRIPOD-SRMA reporting guideline. This review was not prospectively registered. The research question, eligibility criteria, and data extraction framework were nonetheless prespecified before data collection commenced and are documented in this protocol.
