# Supplementary material for: Systematic review of risk prediction models for catheter-associated urinary tract infection
Source: Front Public Health. 2026 Sep 30;14:1884134. doi: 10.3389/fpubh.2026.1884134 (PMC13662166; doi:10.3389/fpubh.2026.1884134)
Supplement: Supplementary file 2 [file Supplementary_file_2.DOCX]

Search strategies for CAUTI risk prediction model systematic review

Search date: 30 June 2026

Databases: PubMed, Embase, Web of Science, Cochrane Library, CNKI, Wanfang Data, VIP, CBM/SinoMed

**Database 1: PubMed (via NLM; n = 195)**

Search Strategy:((((((Catheter-Related Infections[MeSH Terms]) OR (Catheter-associated urinary tract infection*[Title/Abstract])) OR (Catheter-related urinary tract infection*[Title/Abstract])) OR (Urinary catheter-associated infection*[Title/Abstract])) OR (Urinary catheter infection*[Title/Abstract])) OR (Indwelling urinary catheter[Title/Abstract])) AND ((((((((Risk Assessment[MeSH Terms]) OR (Prediction[Title/Abstract])) OR (Model[Title/Abstract])) OR (Predictive Model[Title/Abstract])) OR (Predictive Tool[Title/Abstract])) OR (Risk Prediction[Title/Abstract])) OR (Risk Score[Title/Abstract])) OR (Nomogram[Title/Abstract]))

**Database 2: Embase (via Elsevier; n = 168)**

Search Strategy:('catheter associated urinary tract infection'/exp OR 'catheter-associated urinary tract infection':ti,ab OR 'catheter-related urinary tract infection':ti,ab OR 'urinary catheter-associated infection':ti,ab OR 'urinary catheter infection':ti,ab OR 'indwelling urinary catheter':ti,ab) AND ('risk assessment'/exp OR 'prediction':ti,ab OR 'prediction model':ti,ab OR 'predictive model':ti,ab OR 'predictive tool':ti,ab OR 'risk prediction':ti,ab OR 'risk score':ti,ab OR 'nomogram':ti,ab OR 'clinical prediction model':ti,ab)

**Database 3: Web of Science (via Clarivate; n = 1,219)**

Search Strategy: TS=("catheter-associated urinary tract infection*" OR "catheter-related urinary tract infection*" OR "urinary catheter-associated infection*" OR "urinary catheter infection*" OR "indwelling urinary catheter") AND TS=(prediction OR "prediction model*" OR "predictive model*" OR "predictive tool*" OR "risk prediction" OR "risk score*" OR nomogram OR "clinical prediction model*" OR "risk assessment")

**Database 4: Cochrane Library (via Wiley; n = 288)**

Search Strategy:

#1 MeSH descriptor: [Catheter-Related Infections] explode all trees

#2 "catheter-associated urinary tract infection*":ti,ab,kw

#3 "catheter-related urinary tract infection*":ti,ab,kw

#4 "urinary catheter-associated infection*":ti,ab,kw

#5 "urinary catheter infection*":ti,ab,kw

#6 "indwelling urinary catheter":ti,ab,kw

#7 #1 OR #2 OR #3 OR #4 OR #5 OR #6

#8 MeSH descriptor: [Risk Assessment] explode all trees

#9 prediction:ti,ab,kw

#10 "prediction model*":ti,ab,kw

#11 "predictive model*":ti,ab,kw

#12 "predictive tool*":ti,ab,kw

#13 "risk prediction":ti,ab,kw

#14 "risk score*":ti,ab,kw

#15 nomogram:ti,ab,kw

#16 "clinical prediction model*":ti,ab,kw

#17 #8 OR #9 OR #10 OR #11 OR #12 OR #13 OR #14 OR #15 OR #16

#18 #7 AND #17

**Database 5: CNKI (中国知网; n = 95)**

Search Strategy:检索字段：主题

( "导尿管相关尿路感染" OR "导管相关尿路感染" OR "留置导尿" OR "尿管相关性尿路感染" OR "导尿管相关性感染" ) AND ( "预测" OR "预测模型" OR "风险预测" OR "预测工具" OR "风险评分" OR "列线图" OR "风险模型" OR "预警模型" OR "评分模型" OR "Nomogram" )

**Database 6: Wanfang Data (万方数据; n = 444)**

Search Strategy: 检索字段：主题

( "导尿管相关尿路感染" OR "导管相关尿路感染" OR "留置导尿管" OR "导尿管相关性感染" OR "尿管相关感染" OR "尿路感染" ) AND ( "预测模型" OR "风险预测" OR "预测" OR "列线图" OR "风险评分" OR "预警模型" OR "风险模型" OR "评分系统" OR "预测工具" OR "Nomogram" )

**Database 7: VIP (维普; n = 92)**

Search Strategy: 检索字段：题名或关键词

( "导尿管相关尿路感染" OR "导管相关尿路感染" OR "留置导尿" OR "尿管相关性感染" OR "导尿管相关性尿路感染" ) AND ( "预测模型" OR "风险预测" OR "列线图" OR "风险评分" OR "预警模型" OR "风险模型" OR "预测" OR "评分系统" )

**Database 8: CBM/SinoMed (中国生物医学文献数据库; n = 258)**

Search Strategy: 检索字段：主题词 + 自由词组合

#1 主题词：导尿管相关感染[不加权：扩展]

#2 自由词："导尿管相关尿路感染"[题名/摘要]

#3 自由词："导管相关性尿路感染"[题名/摘要]

#4 自由词："留置导尿管"[题名/摘要]

#5 自由词："尿管相关感染"[题名/摘要]

#6 #1 OR #2 OR #3 OR #4 OR #5

#7 主题词：风险评估[不加权：扩展]

#8 自由词："预测模型"[题名/摘要]

#9 自由词："风险预测"[题名/摘要]

#10 自由词："列线图"[题名/摘要]

#11 自由词："风险评分"[题名/摘要]

#12 自由词："预警模型"[题名/摘要]

#13 自由词："风险模型"[题名/摘要]

#14 自由词："预测工具"[题名/摘要]

#15 #7 OR #8 OR #9 OR #10 OR #11 OR #12 OR #13 OR #14

#16 #6 AND #15
