# Supplementary material for: Systematic review of risk prediction models for catheter-associated urinary tract infection
Source: Front Public Health. 2026 Sep 30;14:1884134. doi: 10.3389/fpubh.2026.1884134 (PMC13662166; doi:10.3389/fpubh.2026.1884134)
Supplement: Supplementary file 3 [file Supplementary_file_3.DOCX]

| No. | First author | Year | Title | Journal/Source | Reason for exclusion |
| --- | --- | --- | --- | --- | --- |
| 1 | Park JI | 2020 | Knowledge discovery with machine learning for hospital-acquired catheter-associated urinary tract infections | CIN: Computers, Informatics, Nursing | no risk prediction model developed |
| 2 | JIANG Shu-fang | 2020 | Establishment and validation of a risk scoring system for catheter-associated urinary tract infection in intensive care unit patients | Chinese Journal of Nosocomiology | Insufficient data to construct prediction model |
| 3 | WEI Nan | 2018 | Construction and validation of a risk prediction scoring model for catheter-associated urinary tract infections in elderly patients | Chinese Journal of Infection Control | Insufficient data to construct prediction model |
| 4 | CHEN Shu-meng | 2025 | Risk factors and prediction model construction for catheter-associated urinary tract infection in intensive care unit | Yunnan Medical Journal | Insufficient data to construct prediction model |
| 5 | TANG Qi-ying | 2024 | Construction of a nomogram prediction model for the risk of catheter-associated urinary tract infection in ICU patients | Guangxi Medical Journal | Insufficient data to construct prediction model |
| 6 | ZHU Jing-wen | 2022 | Establishment and evaluation of a risk prediction model for catheter-associated urinary tract infection in ICU patients [Master's thesis] | Guangxi Medical University | Insufficient data to construct prediction model |
| 7 | LIU Yu-ting | 2024 | Construction and application of a risk prediction model for catheter-associated urinary tract infection in neurosurgical intensive care unit patients [Master's thesis] | Yangzhou University | duplicate data |

Figure 1. PRISMA flow diagram of study selection.
