# Supplementary material for: Systematic review of risk prediction models for catheter-associated urinary tract infection
Source: Front Public Health. 2026 Sep 30;14:1884134. doi: 10.3389/fpubh.2026.1884134 (PMC13662166; doi:10.3389/fpubh.2026.1884134)
Supplement: Supplementary file 4 [file Supplementary_file_4.DOCX]

| Study | Model | Intended prediction time point | Prediction horizon (start → end) | Predictors measured after prediction time point | Predictors potentially affected by developing outcome | Risk of temporal leakage |
| --- | --- | --- | --- | --- | --- | --- |
| LIU Huiying 2025 | LR | At or near hospital admission; model intended for early identification of at-risk stroke inpatients with indwelling catheters | Hospital admission → CAUTI diagnosis or catheter removal during hospitalisation | Indwelling catheter duration (time-varying, accumulates over hospitalisation); Combined antibiotic use (initiated post-admission) | Indwelling catheter duration (post-event accumulation confirmed: median 21 days in CAUTI group vs 7 days in non-CAUTI group); combined antibiotic use (38% of CAUTI patients received antibiotics for concurrent infections during the same admission) | High: two of five final nomogram predictors are unavailable at admission |
| LI Mengchao 2025 | LR | At catheter insertion or hospital admission; model intended for early identification of spinal cord injury patients at risk of post-operative CAUTI | Catheter insertion → CAUTI diagnosis during hospitalisation | Catheter dwell time (time-varying, accumulates over hospitalisation); antibiotic use duration (post-admission variable) | Catheter dwell time (90.63% of CAUTI group had catheter dwell time >30 days vs 31.48% in non-CAUTI group, reflecting post-event accumulation); Antibiotic use duration (accumulates post-admission) | High: catheter dwell time and antibiotic use duration — two of five final predictors — are unavailable at catheter insertion. |
| Sufriyana 2025 | RR, DT, RF, XGBoost, DI-VNN | Daily repeated assessment during each catheterisation episode; predictions generated each day for the subsequent risk window | Each assessment day → CAUTI onset within a 3-day span over a 15-day range, assessed daily during the catheterisation episode | None: the model is explicitly designed as a daily repeated-measures prediction tool; duration of catheterisation is defined as days from catheter start to the day of prediction (time-varying by design, not by inadvertent leakage) | None identified: ICU stay ≥10 days and hospital stay ≥4 days are time-varying but their use is consistent with the daily prediction framework; no reverse causality identified for the daily prediction design | Low: the use of time-varying predictors is a deliberate and methodologically coherent modelling choice; the model is not intended for use at a single admission time point; rated PN in D2.3 but domain overall RoB rated Low |
| ZHU Rui 2024 | LR | At ICU admission or catheter insertion; model intended for early identification of high-risk elderly critically ill patients during ICU admission | ICU admission → CAUTI diagnosis during ICU stay | Catheter dwell time (categorised as 1–6 d, 7–9 d, ≥10 d; time-varying, accumulates over hospitalisation); number of catheter insertions (1, 2, ≥3; time-varying) | Catheter dwell time ≥10 days: 75.44% in CAUTI group vs 19.65% in non-CAUTI group; number of catheter insertions (39.47% of CAUTI group had ≥2 insertions vs 18.20% in non-CAUTI group) | High: two of five final nomogram predictors (catheter dwell time and number of catheter insertions) are time-varying variables unknowable at ICU admission |
| SUN Qing 2024 | LR | At catheter insertion or hospital admission; model intended to guide early preventive interventions | Catheter insertion → CAUTI diagnosis during hospitalisation | Catheter days ≥7 (time-varying cumulative exposure); ICU stay ≥7 days (time-varying cumulative exposure); number of antibiotic types ≥3 (time-varying cumulative exposure) | All three time-varying predictors are cumulative exposures measured over the same period during which CAUTI develops; catheter duration and ICU stay accumulate concurrently with outcome ascertainment; antibiotic exposure similarly accumulates during the risk period | High: three of four final predictors (75%) are time-varying and unavailable at catheter insertion; only nervous system disease is a baseline variable. |
| WANG Fang 2023 | LR | At early stage of treatment, i.e., at or near the time of surgery or catheter insertion; model intended to help physicians assess UTI risk in patients with cervical cancer at early stage | Catheter insertion (post-radical hysterectomy) → UTI diagnosis during post-operative hospitalisation and follow-up until bladder residual urine <100 mL | Indwelling catheterisation duration ≥14 days (time-varying, cannot be known at catheter insertion); Secondary catheterisation (may reflect post-operative bladder dysfunction developing after index catheter insertion) | Indwelling catheterisation duration (64.8% of infected patients had catheter duration >28 days vs 24.3% of non-infected patients, reflecting post-event accumulation); secondary catheterisation (reflects post-operative clinical trajectory) | High: indwelling catheterisation duration — the single most influential predictor (highest coefficient weight in nomogram) — is fundamentally unknowable at catheter insertion |
| LIU Yuting 2023 | CHAID, CRT | At NICU admission; model explicitly designed for use at admission for early identification of high-risk patients | NICU admission → CAUTI diagnosis during NICU stay | Time of indwelling catheter (incorporated as a splitting variable in the CRT decision tree; time-varying, unknowable at admission); length of stay in NICU (time-varying) | Time of indwelling catheter (incorporated in CRT tree as a splitting variable; P=0.001; accumulates concurrently with outcome ascertainment); NICU length of stay (similarly concurrent) | High: the CRT tree incorporates catheter dwell time as a splitting variable, yet the stated application is admission-time risk identification. The final scoring model excludes this variable but is derived from a tree built with temporal leakage; the development process is therefore contaminated. Derived from the same cohort as LI Yuping 2023. |
| LI Yuping 2023 | LR | At NICU admission; model intended for early risk identification; three of four predictors can be assessed on admission | NICU admission → CAUTI diagnosis during NICU stay | Length of neuro-ICU stay >30 days (time-varying, fundamentally unknowable at NICU admission) | Length of neuro-ICU stay >30 days (OR=272.2, β=5.067; this predictor is defined over the same time window as CAUTI ascertainment, making it intrinsically confounded with the outcome) | High: length of neuro-ICU stay >30 days is the dominant predictor (OR=272.2) but is unknowable at NICU admission; the OR magnitude likely reflects reverse causality rather than predictive signal. Derived from the same cohort as LIU Yuting 2023. |
| XIA Liyao 2022 | LR | At time of postoperative transurethral catheter insertion; model intended for early identification of high-risk patients among postoperative gynecologic malignancy patients | Catheter insertion (post-surgery) → CAUTI diagnosis during hospitalisation and post-discharge follow-up (telephone follow-up every two weeks after discharge) | Indwelling catheter duration >7 days (time-varying, cannot be known at catheter insertion); Postoperative bladder irrigation (may reflect intraoperative urinary tract injury; not fully determinable at catheter insertion) | Indwelling catheter duration >7 days (predictor with largest regression coefficient β=1.57, OR=4.78 in the scoring model; accumulates concurrently with outcome ascertainment); postoperative bladder irrigation (determined by post-operative clinical course) | High: indwelling catheter duration >7 days — the highest-weighted predictor in the scoring model — is fundamentally unknowable at catheter insertion |
| LÜ Yangyang 2022 | LR | At catheter insertion in the emergency room; model intended for risk assessment at the time of catheterisation | Catheter insertion (emergency room) → CAUTI diagnosis during emergency room stay | Catheter dwell time ≥3 days (time-varying, cannot be known at the time of catheter insertion) | Catheter dwell time ≥3 days (OR=3.135, β=1.143; the largest regression coefficient among all five final predictors; accumulates concurrently with outcome ascertainment) | High: catheter dwell time ≥3 days (OR=3.135, β=1.143) is unavailable at catheter insertion. |
| LIAO Liping 2022 | LR | At catheter insertion or ICU/neurosurgery admission; model intended for early identification of high-risk TBI patients | Hospital/ICU admission → CAUTI diagnosis during hospitalisation | Indwelling catheter ≥30 days (time-varying, unknowable at admission); ICU stay ≥14 days (time-varying, unknowable at admission); antibiotic use duration ≥20 days (time-varying, unknowable at admission) | Indwelling catheter ≥30 days (OR=6.170; CAUTI risk 6.1 times that of patients with catheter duration <30 days; accumulates concurrently with outcome); ICU stay ≥14 days (OR=5.255; accumulates concurrently with outcome); both predictors affect 40% of final model predictors simultaneously | High: two of five final nomogram predictors are time-varying variables with large effect estimates (OR=6.170 and OR=5.255) unknowable at admission |
| LI Zhe 2021 | LR | At catheter insertion in neurosurgery ward; model intended for early warning of CAUTI | Catheter insertion → CAUTI diagnosis during hospitalisation | ICU admission (time-varying; determined during hospitalisation, not at catheter insertion); prophylactic antibiotic use (time-varying; initiated during hospitalisation) | ICU admission (risk of reverse causality: CAUTI aggravation may lead to ICU admission); prophylactic antibiotic use (may be prescribed in response to early infection signs rather than administered prophylactically before CAUTI onset) | High: ICU admission and prophylactic antibiotic use are determined during hospitalisation and unavailable at catheter insertion; reverse causality cannot be excluded for both variables. |
| Eun Young Hur 2019 | LR | During hospitalisation, with daily updated risk scores; system intended for automated real-time CAUTI surveillance | Hospital admission → CAUTI diagnosis (cases defined as positive urine culture on or after hospital day 3) | Total length of stay (end-of-stay cumulative value, unknowable during early hospitalisation); total indwelling urinary catheter duration (end-of-stay cumulative value, unknowable during early hospitalisation) | Total length of stay (reverse causality: infection itself prolongs hospitalisation); total catheter duration (reverse causality: CAUTI prolongs catheterisation); both predictors are on the causal pathway with CAUTI; default system rules also incorporate urine WBC, bacteria on microscopy, and urine culture ≥10³ CFU/mL — early markers of the outcome itself | High: total length of stay and total catheter duration are end-of-stay values trained on final totals, yet the model is intended for daily use with partial-duration inputs — a fundamental mismatch. Default rules incorporate urine WBC, bacteriuria, and urine culture results, which are early markers of the outcome itself. Control group selected from non-catheterised patients, misaligning with the target population. |

LR, Logistic regression; RF, Random forest; RR, Ridge regression; DT, Decision tree; CHAID, Chi-squared automatic interaction detector; CRT, Classification and regression tree; XGBoost, Extreme gradient boosting; DI-VNN, Deep integrated variational neural network
