# Supplementary material for: Systematic review of risk prediction models for catheter-associated urinary tract infection
Source: Front Public Health. 2026 Sep 30;14:1884134. doi: 10.3389/fpubh.2026.1884134 (PMC13662166; doi:10.3389/fpubh.2026.1884134)
Supplement: Supplementary file 5 [file Supplementary_file_5.DOCX]

Here are the PROBAST completion rules translated into English:

- Y = Yes (low-risk signal); PY = Probably Yes; N = No (high-risk signal); PN = Probably No; NI = No Information
- A domain is rated High RoB if ≥1 signalling question within that domain is answered N or PN
- Overall RoB is rated Low only when all four domains are rated Low; otherwise Overall RoB is rated High

**LIU Huiying Liu Huiying 2025**

**Report:** Liu HY, Xiao Y, Huang J, et al. Risk factors and prediction model for catheter-associated urinary tract infections in stroke patients with indwelling urinary catheters. *Guangdong Medical Journal.* 2025; 46(3): 459–465.

**Domain 1 — Participants**

|  | **Signalling Question** | **Response** | **Justification** |
| --- | --- | --- | --- |
| D1.1 | Were appropriate data sources used? | Y | Data were collected from electronic medical records of stroke patients with indwelling urinary catheters admitted to the neurology and neurosurgery departments at Zhongshan Traditional Chinese Medicine Hospital between January 2021 and December 2022. This is an appropriate data source for a CAUTI prediction model in stroke inpatients. Hospital infection control records are a standard and accepted data source for this type of research question. |
| D1.2 | Were all inclusions and exclusions of participants appropriate? | Y | Inclusion criteria required confirmed stroke diagnosis (ischaemic or haemorrhagic), age ≥18 years, negative urinalysis (white blood cells, nitrites) and negative urine culture at 48 hours post-admission, and meeting the diagnostic criteria for CAUTI (urinary tract infection occurring during indwelling catheterisation or within 48 hours after catheter removal). Exclusion criteria included diagnosis of only transient ischaemic attack or cerebral venous sinus thrombosis, hospitalisation ≤2 days, urinary tract infection at admission, and incomplete clinical data. These criteria are clinically appropriate and ensure that only genuinely at-risk patients are included, pre-existing infection appropriately excluded at baseline. |
| D1.3 | Were participants enrolled at a similar and relevant point in their disease or condition? | PY | Patients were enrolled at hospital admission, with urinary tract infection status confirmed negative at 48 hours post-admission. Enrolment thus occurs at or close to the start of the risk period, which is appropriate. However, the 48-hour window introduces a minor ambiguity: patients enrolled are those who survived at least 48 hours without infection, which may slightly select against the most severely ill patients with very early CAUTI. No evidence of prevalent cohort bias in the strict sense. |
| D1.4 | Was the sample size appropriate? | PN | Among the 699 patients, 84 cases of CAUTI were identified. The cohort was randomly divided into a training set (70%) and a validation set (30%). The training set therefore contained approximately 489 patients with approximately 57 CAUTI events. Multivariate logistic regression included age, NIHSS score, length of hospital stay, indwelling catheter duration, antibiotic exposure duration, ICU stay, combined antibiotic use, and quinolone use — at least 8 candidate variables entered into the multivariable model. With 57 events and 8 candidate variables in the training set, EPV ≈ 7.1. This falls below the commonly cited threshold of 10 and well below more contemporary recommendations of EPV ≥ 20 for stable logistic regression models. |

**Domain 1 overall RoB: H**

Rationale: D1.4 is rated PN due to inadequate EPV (~7.1) in the training set. D1.1–D1.3 are satisfactory.

**Domain 1 Applicability: H**

The study population consists exclusively of stroke patients (ischaemic and haemorrhagic) with indwelling urinary catheters admitted to the neurology and neurosurgery departments of a single tertiary Traditional Chinese Medicine hospital in Zhongshan, Guangdong. This is a highly specific population (stroke only, single centre, Traditional Chinese Medicine hospital setting) that may differ systematically from general medical wards, mixed ICU populations, or non-stroke catheterised patients. Applicability to broader catheterised inpatient populations is limited.

**Domain 2 — Predictors**

|  | **Signalling Question** | **Response** | **Justification** |
| --- | --- | --- | --- |
| D2.1 | Were predictors defined and assessed in a similar way for all participants? | PY | Clinical data were retrospectively collected from the electronic medical record system, including age, sex, length of hospitalisation, indwelling catheter duration, NIHSS score, modified Rankin Scale (MRS) score, antibiotic exposure duration, use of quinolones, combined antibiotic use, and comorbidities including diabetes, hypertension, renal insufficiency, and immune dysfunction. These are routinely recorded variables in standardised electronic systems. No formal data dictionary or inter-rater reliability assessment is reported, but objective administrative and clinical variables are unlikely to suffer from substantial measurement inconsistency across patients. |
| D2.2 | Were predictor assessments made without knowledge of outcome data? | PY | Most predictors (age, sex, NIHSS score, MRS score, comorbidities) are fixed or measured at or near admission, independent of subsequent CAUTI status. Retrospective extraction of these values from the medical record is unlikely to have been influenced by knowledge of outcome. However, some uncertainty exists for variables such as antibiotic exposure duration and combined antibiotic use, which reflect evolving clinical decisions during admission rather than a single time-point measurement. |
| D2.3 | Are all predictors available at the time the model is intended to be used? | PN | The model is intended to help early identification of stroke patients with indwelling urinary catheters at risk of CAUTI. The five independent predictors incorporated into the nomogram are age, NIHSS score, indwelling catheter duration, combined antibiotic use, and ICU stay. Of these, **indwelling catheter duration** is a time-varying variable that accumulates over the course of hospitalisation and cannot be known at admission. In the training set, the median indwelling catheter duration in CAUTI patients was 21.00 (IQR 12.00–35.00) days, compared with 7.00 (IQR 4.00–13.00) days in non-CAUTI patients — a difference that reflects post-event accumulation rather than a baseline predictor. Similarly, combined antibiotic use in 38% of CAUTI patients was initiated in response to infections developing during the same admission, making it a post-admission variable rather than a fixed baseline predictor. These constitute temporal leakage: the model cannot be applied at admission as intended because key predictors are not available at that time point. |

**Domain 2 overall RoB: H**

Rationale: D2.3 is rated PN. Indwelling catheter duration — one of the five final predictors in the nomogram — is intrinsically a time-varying variable that accumulates over the hospitalisation course and is not available at admission. This represents a fundamental temporal leakage problem that undermines the stated clinical utility of the model for early identification of at-risk patients.

**Domain 2 Applicability: L**

The predictors used (age, NIHSS score, ICU stay, antibiotic use, catheter duration) are standard clinical variables available in routine stroke inpatient care. No proprietary instruments or highly specialised measurements are required.

**Domain 3 — Outcome**

|  | **Signalling Question** | **Response** | **Justification** |
| --- | --- | --- | --- |
| D3.1 | Was the outcome determined appropriately? | PY | The diagnostic criterion for CAUTI was defined as urinary tract infection occurring during indwelling catheterisation or within 48 hours after catheter removal, with negative urinalysis confirmed at 48 hours post-admission. Microbiological confirmation is implied but the specific culture threshold and symptomatic criteria are not explicitly stated in the methods section. |
| D3.2 | Was a pre-specified or standard outcome definition used? | PN | The CAUTI definition provided in the inclusion criteria — "urinary tract infection occurring during indwelling catheterisation or within 48 hours after catheter removal" — is a clinical/temporal definition rather than a full microbiologically confirmed standard definition. The study does not specify whether the NHSN/CDC definition or Chinese national infection surveillance standards were applied. No urine culture threshold (e.g., 10³ or 10⁵ CFU/mL) or symptom criteria are stated. The outcome definition is therefore incompletely reported and cannot be confirmed as a pre-specified standard definition. |
| D3.3 | Were predictors excluded from the outcome definition? | Y | The CAUTI outcome is a microbiologically and temporally defined infection event. None of the model predictors (age, NIHSS score, catheter duration, antibiotic use, ICU stay) are components of the outcome definition itself. |
| D3.4 | Was the outcome assessed without knowledge of predictor values? | U | No information is provided on whether clinicians or infection control staff who ascertained CAUTI status were blinded to predictor values. In a retrospective electronic record study, formal blinding is not performed. This cannot be confirmed or refuted. |
| D3.5 | Was the time interval between predictor assessment and outcome determination appropriate? | PN | The follow-up period covers the duration of hospitalisation. As noted in D2.3, indwelling catheter duration and combined antibiotic use accumulate during the same period in which CAUTI is being ascertained. The time interval between "predictor assessment" and "outcome" is therefore not cleanly separable for these two variables — they are effectively measured concurrently with, or as a consequence of, the outcome process. |
| D3.6 | Was there a sufficient proportion of participants with the outcome? | PN | Among the 699 patients, 84 cases of CAUTI were identified (12.02%). The overall event rate of 12% is clinically reasonable. However, the random 70/30 split yields approximately 57 events in the training set, which is marginal for stable logistic regression model development (see D1.4 and D4.1). |

**Domain 3 overall RoB: H**

Rationale: D3.2 is rated PN due to incomplete and non-standard outcome definition reporting (no urine culture threshold, no symptom criteria specified). D3.5 is rated PN due to concurrent measurement of key predictors and the outcome. D3.6 is rated PN due to the small number of events in the training set.

**Domain 3 Applicability: H**

The incomplete outcome definition makes it difficult to confirm comparability with other studies using full NHSN/CDC criteria. If a less stringent definition was applied, the CAUTI rate and case mix may not be comparable to settings using the standard three-condition criterion.

**Domain 4 — Analysis**

|  | **Signalling Question** | **Response** | **Justification** |
| --- | --- | --- | --- |
| D4.1 | Were there an appropriate number of participants with the outcome? | PN | The training set contained approximately 489 participants with 57 CAUTI events. Eight variables were entered into the multivariable model (age, NIHSS score, length of hospital stay, indwelling catheter duration, antibiotic exposure duration, ICU stay, combined antibiotic use, and quinolone use). EPV ≈ 57/8 = 7.1, which is below the commonly accepted threshold of 10 and substantially below the more rigorous contemporary threshold of 20 for stable prediction models. |
| D4.2 | Were continuous and categorical predictors handled appropriately? | PN | Age, NIHSS score, indwelling catheter duration, and antibiotic exposure duration were treated as continuous variables in the regression analysis. This is appropriate in principle. However, the nomogram presents age across a range of 25–95, and catheter duration across 0–65 days, suggesting continuous modelling. No assessment of linearity on the log-odds scale (e.g., restricted cubic splines or fractional polynomials) is reported. The assumption of log-linear relationships for continuous predictors is not tested or acknowledged. |
| D4.3 | Were all enrolled participants included in the analysis? | PN | Patients with incomplete clinical data were excluded. The number of excluded patients and the nature of missing data are not reported. It is therefore unclear whether the exclusion of incomplete cases introduced selection bias, and the extent of data missingness cannot be evaluated. |
| D4.4 | Were missing data handled appropriately? | N | As noted in D4.3, patients with incomplete clinical data were excluded with no description of the missing data mechanism, the number of affected patients, or any sensitivity analysis. Complete-case analysis without justification is inappropriate when the missingness mechanism is unknown. No imputation strategy is reported. |
| D4.5 | Was selection of predictors based on univariable analysis avoided? | N | Univariate and multivariate logistic regression analyses were used to identify independent risk factors: variables found significant in univariate analysis were entered into the multivariate model. This two-stage approach — univariable screening followed by multivariable modelling — is known to introduce selection bias, may exclude important variables with moderate univariable associations, and inflates optimism in the final model. This is explicitly flagged as a methodological limitation by contemporary prediction modelling guidelines |
| D4.6 | Were complexities in the data accounted for appropriately? | U | No information is provided on whether potential clustering effects (e.g., admission period, treating physician, ward) or the hierarchical structure of the data were considered. The study spans two years across two departments (neurology and neurosurgery), and potential within-cluster correlation is not addressed. |
| D4.7 | Were relevant model performance measures evaluated appropriately? | PY | The model was evaluated using ROC curve (AUC), calibration curve with Bootstrap 1000-iteration internal resampling (Hosmer-Lemeshow test P = 0.341 for training set), and decision curve analysis (DCA). The validation set AUC was 0.758 (95% CI: 0.661–0.855), and the validation set calibration curve showed P = 0.621. AUC, calibration curve, and DCA together represent a reasonably comprehensive performance evaluation. However, calibration is reported only via Hosmer-Lemeshow test P-value, without a calibration plot slope or intercept, and Brier score is not reported. |
| D4.8 | Were model overfitting and optimism accounted for? | PN | Bootstrap resampling (1000 iterations) was used for internal validation, and the calibration curve showed good agreement between predicted and observed probabilities (P = 0.341). Bootstrap calibration was performed, which is a positive feature. However, it is unclear whether optimism-corrected (shrinkage-adjusted) performance estimates are reported, or whether the bootstrap was used solely for calibration plot construction. Given the low EPV of ~7.1 and the use of univariable pre-selection, the reported AUC of 0.857 is likely optimistic. |
| D4.9 | Do predictors and their assigned weights correspond to the multivariable analysis results? | Y | The regression coefficients, standard errors, Z-statistics, P-values, and odds ratios with 95% CIs are reported for all variables in the multivariable model in Table 3. The nomogram is constructed directly from these multivariable results. The predictor weights in the nomogram are internally consistent with the reported regression analysis. |
| D4.10 | Was model performance evaluated in different subgroups of participants? | N | No subgroup performance analyses are reported. Given that the model is intended for use in both ischaemic and haemorrhagic stroke patients, and across neurology and neurosurgery departments, the absence of subgroup analyses represents a missed opportunity to assess heterogeneity of model performance. |
| D4.11 | Was model performance evaluated using calibration? | PY | Calibration was assessed using calibration curves with Bootstrap resampling in the training set (Hosmer-Lemeshow P = 0.341) and in the validation set (P = 0.621). Calibration curves are presented visually (Figures 3 and 6). However, calibration slope and intercept are not reported numerically, and Brier score is absent. Visual inspection of the calibration plots (Figures 3 and 6) suggests reasonable calibration, but formal quantitative calibration metrics beyond the H-L test P-value are lacking. |
| D4.12 | Was model performance evaluated in an external validation dataset? | PN | The cohort was randomly divided into a training set (70%) and a validation set (30%). This is a random split-sample internal validation, not external validation. A random split of a single cohort does not constitute independent external validation, as both sets share the same patient population, time period, institution, and case-mix. No external validation in an independent cohort has been performed. |

**Domain 4 overall RoB: H**

Rationale: Multiple significant analytical problems: inadequate EPV (~7.1) in the training set; univariable pre-screening for predictor selection; complete-case analysis without missing data handling; no test of linearity assumptions for continuous variables; random split-sample validation only (not external validation); and incomplete calibration reporting. These collectively compromise the reliability of the reported performance estimates.

**Overall Judgment Summary**

| Domain | Risk of Bias | Applicability |
| --- | --- | --- |
| D1 Participants | H | H |
| D2 Predictors | H | L |
| D3 Outcome | H | H |
| D4 Analysis | H | — |
| Overall | H | H |

**PROBAST Assessment: Li Mengchao 2025**

**Report:** Li MC, Lü H, Sun QX, Zhang Y. Construction and validation of a nomogram for predicting catheter-associated urinary tract infection risk after spinal cord injury surgery. *J Med Theor & Prac.* 2025; 38(18): 3200–3202.

**Domain 1 — Participants**

|  | **Signalling Question** | **Response** | **Justification** |
| --- | --- | --- | --- |
| D1.1 | Were appropriate data sources used? | Y | Data were collected from the hospital electronic information system from patients with spinal cord injury admitted to the spinal surgery department of the Affiliated Hospital of Jiujiang University. An institutional electronic record system is an appropriate data source for CAUTI prediction modelling in surgical inpatients with spinal cord injury. |
| D1.2 | Were all inclusions and exclusions of participants appropriate? | PY | Inclusion criteria required imaging-confirmed spinal cord injury with surgical treatment, negative urine culture at admission, stable disease status, and age >18 years. CAUTI diagnosis in the infection group was required to meet the 2009 IDSA international clinical practice guidelines. Exclusion criteria included spinal shock stage, combined cardiopulmonary organ dysfunction, urinary tract malformation, and incomplete data. These criteria are clinically appropriate and ensure that only patients genuinely at risk of post-operative CAUTI are included. However, the exclusion of patients with "incomplete data" without reporting how many were excluded or whether missingness was related to outcome introduces uncertainty. |
| D1.3 | Were participants enrolled at a similar and relevant point in their disease or condition? | PY | Inclusion required negative urine culture at admission, and patients in spinal shock were excluded. Enrolment therefore occurs at the start of the relevant risk period (post-operative catheterisation), which is appropriate. However, the exact enrolment time point relative to catheter insertion is not explicitly stated, and the study spans over three years (July 2020 to November 2023), during which institutional infection control practices may have changed. |
| D1.4 | Was the sample size appropriate? | N | Of the 140 patients, 32 developed CAUTI (infection rate 22.86%). Univariate analysis identified statistically significant variables which were then entered into logistic regression analysis. The candidate variables entered into logistic regression included: sex, catheter dwell time, antibiotic use duration, stress hyperglycaemia, fluid intake, urinary bag replacement frequency, neurogenic bladder, ASIA score, number of catheterisations, and serum albumin — at least 10 candidate variables. With 32 events and 10 candidate variables, EPV = 3.2, critically below any accepted threshold for stable logistic regression. No sample size calculation is reported. |

**Domain 1 overall RoB: H**

Rationale: D1.4 is rated N due to a critically low EPV of 3.2 (32 events, 10 candidate variables). D1.1–D1.3 are satisfactory. The high-risk judgment is driven entirely by the severely inadequate sample size, not by retrospective design.

**Domain 1 Applicability: H**

The study was conducted exclusively in patients with spinal cord injury undergoing surgical treatment at a single tertiary hospital in Jiujiang, Jiangxi province. This is a highly specialised surgical population in a single-centre setting. The model's applicability is restricted to post-operative spinal cord injury patients and may not generalise to non-surgical SCI patients, catheterised medical inpatients, or multi-centre settings.

**Domain 2 — Predictors**

|  | **Signalling Question** | **Response** | **Justification** |
| --- | --- | --- | --- |
| D2.1 | Were predictors defined and assessed in a similar way for all participants? | PY | Clinical data were collected through the hospital electronic information system, including age, sex, catheter dwell time, catheter type, antibiotic use duration, stress hyperglycaemia, fluid intake, urinary bag replacement frequency, neurogenic bladder, ASIA score, number of catheterisations, and serum albumin level. These are standard clinical variables recorded in routine electronic records. No formal data dictionary or inter-rater reliability assessment is reported, but objective administrative and laboratory variables are unlikely to suffer from systematic measurement inconsistency across patients. |
| D2.2 | Were predictor assessments made without knowledge of outcome data? | PY | Fixed or early-measured predictors (age, sex, ASIA score, neurogenic bladder, serum albumin at admission) are unlikely to have been influenced by knowledge of CAUTI outcome in a retrospective electronic record extraction. However, stress hyperglycaemia, antibiotic use duration, urinary bag replacement frequency, and number of catheterisations reflect evolving clinical management decisions during the admission and may have been recorded or influenced by awareness of developing infection. |
| D2.3 | Are all predictors available at the time the model is intended to be used? | N | The nomogram was intended to enable early identification of patients at risk of CAUTI. However, catheter dwell time and ASIA score are among the final independent predictors incorporated into the nomogram. Catheter dwell time is a time-varying variable that accumulates over the hospitalisation course and is intrinsically unknowable at the time of intended model use (admission or catheter insertion). In the univariate analysis, 90.63% of the infection group had catheter dwell time >30 days versus only 31.48% in the non-infection group — a difference that reflects post-event accumulation rather than a true baseline predictor. Furthermore, antibiotic use duration is similarly a post-admission variable that cannot be known at the point of early risk identification. These constitute temporal leakage: the model cannot be applied at admission as intended because key predictors are not available at that time. |

**Domain 2 overall RoB: H**

Rationale: D2.3: N. Catheter dwell time and antibiotic use duration — two of five final predictors — are unavailable at catheter insertion. Temporal leakage undermines the stated early-identification purpose.

**Domain 2 Applicability: L**

The predictors used (ASIA score, serum albumin, stress hyperglycaemia, neurogenic bladder, catheter dwell time) are standard clinical variables in spinal cord injury care. ASIA score is a validated, widely used neurological classification tool. No proprietary or highly specialised measurements are required.

**Domain 3 — Outcome**

|  | **Signalling Question** | **Response** | **Justification** |
| --- | --- | --- | --- |
| D3.1 | Was the outcome determined appropriately? | PY | CAUTI diagnosis was required to meet the 2009 IDSA international clinical practice guidelines for the diagnosis, prevention, and treatment of catheter-associated urinary tract infection in adults. Use of a published, internationally recognised guideline is appropriate. |
| D3.2 | Was a pre-specified or standard outcome definition used? | PY | The 2009 IDSA guideline is a recognised standard definition for CAUTI. However, the specific criteria applied (e.g., urine culture threshold, symptom requirements) are not explicitly stated in the methods section, making it impossible to confirm full adherence to the standard. The 2009 IDSA definition requires both microbiological confirmation and the presence of signs or symptoms attributable to the urinary tract, which are not described in the results. |
| D3.3 | Were predictors excluded from the outcome definition? | Y | The CAUTI outcome is defined by microbiological and clinical criteria per the IDSA guideline. None of the model predictors (ASIA score, albumin, stress hyperglycaemia, neurogenic bladder, catheter dwell time) are components of the outcome definition. |
| D3.4 | Was the outcome assessed without knowledge of predictor values? | U | No information is provided on whether clinicians or infection control staff who determined CAUTI status were blinded to predictor values. In a retrospective electronic record study, formal blinding is not performed and cannot be confirmed. |
| D3.5 | Was the time interval between predictor assessment and outcome determination appropriate? | PN | As noted in D2.3, catheter dwell time and antibiotic use duration are measured concurrently with, or as a cumulative consequence of, the hospitalisation period during which CAUTI is being ascertained. The temporal separation between predictor measurement and outcome determination is therefore not cleanly established for these two predictors. |
| D3.6 | Was there a sufficient proportion of participants with the outcome? | N | Of the 140 patients, 32 developed CAUTI, an infection rate of 22.86%. The event rate of 22.86% is clinically plausible for this population, but the absolute number of 32 events is critically small for logistic regression model development with 10 candidate variables (EPV = 3.2). This is insufficient for any accepted standard of stable prediction model development. |

**Domain 3 overall RoB: H**

Rationale: D3.5 is rated PN due to concurrent measurement of key predictors (catheter dwell time, antibiotic duration) and the outcome. D3.6 is rated N due to the critically small absolute number of events (32). D3.2 is rated PY given the reference to a recognised guideline, but the specific diagnostic criteria applied are incompletely reported.

**Domain 3 Applicability: L**

The IDSA 2009 guideline is an internationally recognised standard definition for CAUTI. Although specific criteria are incompletely described, the stated use of this guideline is broadly consistent with definitions used in other included reports, supporting reasonable comparability.

**Domain 4 — Analysis**

|  | **Signalling Question** | **Response** | **Justification** |
| --- | --- | --- | --- |
| D4.1 | Were there an appropriate number of participants with the outcome? | N | With 32 CAUTI events and 10 candidate variables entered into logistic regression, EPV = 3.2. This is critically inadequate. The model incorporates five final predictors: stress hyperglycaemia, catheter dwell time, neurogenic bladder, ASIA score, and serum albumin. Even relative to the final model (EPV = 32/5 = 6.4), the sample remains insufficient. The candidate EPV of 3.2 is the more relevant metric, as model selection was data-driven. |
| D4.2 | Were continuous and categorical predictors handled appropriately? | PN | Serum albumin level was treated as a continuous variable (measured value). ASIA score was coded as an ordinal variable (E=0, D=1, C=2, B=3, A=4). Catheter dwell time was dichotomised (≤30d vs >30d). Antibiotic use duration was dichotomised (≤15d vs >15d). Dichotomisation of continuous and ordinal variables (catheter dwell time, antibiotic duration, number of catheterisations) is a recognised methodological limitation that loses information, introduces threshold instability, and artificially inflates apparent model performance. No assessment of linearity for continuous predictors (albumin) on the log-odds scale is reported. |
| D4.3 | Were all enrolled participants included in the analysis? | PN | Patients with incomplete data were excluded (criterion 4 of exclusion criteria). The number of patients excluded for incomplete data and the nature of missing variables are not reported. It is therefore impossible to assess whether this exclusion introduced selection bias. |
| D4.4 | Were missing data handled appropriately? | N | As noted in D4.3, patients with incomplete data were simply excluded with no description of the missing data mechanism, the number affected, or any sensitivity analysis. Complete-case analysis without justification is inappropriate when the missingness mechanism is unknown and potentially related to outcome severity. |
| D4.5 | Was selection of predictors based on univariable analysis avoided? | N | Variables with statistical significance in univariate analysis (P<0.05) were entered as independent variables into logistic regression. This two-stage approach — univariable screening followed by multivariable modelling — is a well-recognised source of optimism and selection bias. It may exclude clinically important predictors that fail to reach significance individually and introduces circularity into the model selection process. This is a critical methodological flaw. |
| D4.6 | Were complexities in the data accounted for appropriately? | U | No information is provided on whether clustering effects (e.g., surgeon, admission period, ward) or temporal trends across the three-year study period were considered. The study spans July 2020 to November 2023, covering the COVID-19 pandemic period, during which infection control practices may have changed substantially. |
| D4.7 | Were relevant model performance measures evaluated appropriately? | PY | Model performance was evaluated using ROC curve (AUC = 0.993, 95% CI 0.984–1.000, sensitivity 96.9%, specificity 96.3%, Youden index 0.932). Calibration was assessed using bootstrap resampling with a calibration curve, and Hosmer-Lemeshow test (χ²=0.336, df=2, P=0.845). AUC, calibration curve, and H-L test together represent a reasonable performance evaluation framework. However, Brier score, calibration slope, and calibration intercept are not reported. Decision curve analysis is absent. |
| D4.8 | Were model overfitting and optimism accounted for? | N | Bootstrap resampling was used to validate the model and generate a calibration curve. However, the bootstrap appears to have been used only for calibration curve construction, not for optimism-corrected discrimination estimates. The reported AUC of 0.993 is an apparent (derivation) performance metric. With an EPV of 3.2 and data-driven variable selection, this figure almost certainly reflects severe overfitting rather than true predictive performance. No shrinkage correction, optimism-corrected AUC, or cross-validation is reported. An apparent AUC of 0.993 with EPV = 3.2 is inconsistent with genuine predictive performance and almost certainly reflects severe overfitting. |
| D4.9 | Do predictors and their assigned weights correspond to the multivariable analysis results? | PY | The logistic regression results report regression coefficients (β), standard errors, Wald statistics, degrees of freedom, P-values, odds ratios, and 95% CIs for all variables in the multivariable model. The nomogram model formula is explicitly stated: logit(P) = 5.788 × stress hyperglycaemia + 7.739 × catheter dwell time + 5.225 × neurogenic bladder + 5.664 × ASIA score − 0.592 × albumin + 4.770. The nomogram is constructed directly from these regression results and is internally consistent. |
| D4.10 | Was model performance evaluated in different subgroups of participants? | N | No subgroup performance analyses are reported. Given that the model is intended for use in spinal cord injury patients with varying ASIA grades (A through E) and varying injury mechanisms, the absence of subgroup analyses is a notable omission. |
| D4.11 | Was model performance evaluated using calibration? | PY | Calibration was assessed using a bootstrap calibration curve, with Hosmer-Lemeshow test χ²=0.336, P=0.845, indicating that the model calibration curve approximated the ideal curve. Calibration curve and H-L test are reported. However, The H-L test with only two degrees of freedom (χ²=0.336, df=2) uses an atypically small number of groups, which reduces the test's ability to detect miscalibration across the probability range. Calibration slope and intercept are not reported numerically. |
| D4.12 | Was model performance evaluated in an external validation dataset? | N | Bootstrap resampling was used to validate the model. Bootstrap resampling is a form of internal validation only. No external validation in an independent cohort is reported. All performance metrics reflect apparent or internally validated derivation performance. |

**Domain 4 overall RoB: H**

Rationale: Multiple critical analytical failures: EPV of 3.2 (critically inadequate); univariable pre-screening for predictor selection; complete-case analysis without missing data handling; dichotomisation of continuous variables without justification; no optimism-corrected discrimination estimate; no external validation; and an implausibly high apparent AUC of 0.993 that almost certainly reflects severe overfitting given the sample constraints.

**Overall Judgment Summary**

| Domain | Risk of Bias | Applicability |
| --- | --- | --- |
| D1 Participants | H | H |
| D2 Predictors | H | L |
| D3 Outcome | H | L |
| D4 Analysis | H | — |
| Overall | H | H |

**PROBAST Assessment: Sufriyana 2025**

**Report:** Sufriyana H, Chen C, Chiu HS, et al. Estimating individual risk of catheter-associated urinary tract infections using explainable artificial intelligence on clinical data. *Am J Infect Control.* 2025; 53: 368–374.

**Domain 1 — Participants**

|  | **Signalling Question** | **Response** | **Justification** |
| --- | --- | --- | --- |
| D1.1 | Were appropriate data sources used? | Y | Data were drawn from the Taipei Medical University Clinical Research Database (TMUCRD), consisting of deidentified, curated electronic medical records from three hospitals. The database covers 4.1 million patients across Taiwan up to 23 years (1998–2021). This is a high-quality, multi-institutional administrative database appropriate for a CAUTI prediction model among hospitalised individuals receiving urinary catheterisation. |
| D1.2 | Were all inclusions and exclusions of participants appropriate? | Y | Data were selected if the subject's age ranged from 20 to 75 years and urinary catheterisation was prescribed. All data from the time of catheterisation initiation to 48 hours post-removal were included, defining a catheterisation "episode." These criteria are clinically appropriate and directly relevant to the research question of CAUTI risk among catheterised inpatients. The age restriction (20–75 years) is acknowledged as a potential limitation but is clearly stated. |
| D1.3 | Were participants enrolled at a similar and relevant point in their disease or condition? | Y | The observational unit was each day within a catheterisation episode, from initiation to 48 hours post-removal. Enrolment begins at the time of catheter insertion, which is the appropriate and clinically relevant starting point for a CAUTI risk prediction model. There is no evidence of prevalent cohort bias; patients with pre-existing CAUTI at catheter insertion are not explicitly described as excluded, but the episode-based design ensures that prediction is assessed from the start of catheterisation. |
| D1.4 | Was the sample size appropriate? | Y | A total of 122,386 instances (catheterisation-days) were included across the three sites. The development set comprised 101,263 instances (13,432 CAUTI-positive; 87,831 CAUTI-negative), split into training (70,334 negative/10,676 positive) and validation (17,497/2,756) sets. The test set comprised 21,123 instances (4,074 CAUTI-positive). With 10,676 events in the training set and 15 final predictors, EPV > 700, which is vastly adequate for all modelling approaches employed including random forest and XGBoost. |

**Domain 1 overall RoB: L**

Rationale: All four signalling questions are satisfactory. The data source is a large, multi-institutional electronic health record database; eligibility criteria are appropriate and transparent; enrolment begins at catheter insertion; and the sample size is by far the largest among all included reports, with EPV exceeding 700.

**Domain 1 Applicability: H**

The study cohort only consisted of an ethnic Chinese population in Taiwan, and the authors note that validation in other ethnicities or countries should be considered. Additionally, data were selected from subjects aged 20 to 75 years, excluding elderly patients over 75 — a group at particularly high risk of CAUTI. The multi-hospital Taiwanese setting and the restricted age range may limit direct applicability to other healthcare systems, ethnic populations, or age groups outside this range.

**Domain 2 — Predictors**

|  | **Signalling Question** | **Response** | **Justification** |
| --- | --- | --- | --- |
| D2.1 | Were predictors defined and assessed in a similar way for all participants? | PY | Twenty candidate predictors were predetermined based on a literature review, and their data availability was identified after data partitioning. Thirty indicators for 19 candidate predictors were identified in the training set. Predictors were extracted from a curated, deidentified electronic medical record database using standardised data definitions. Consistent extraction procedures across a centralised database system support uniform predictor assessment. However, the database covers 23 years (1998–2021), during which coding practices, diagnostic criteria, and clinical documentation standards may have changed, introducing potential temporal inconsistency in predictor measurement. |
| D2.2 | Were predictor assessments made without knowledge of outcome data? | PY | Predictors were extracted from electronic health records in a retrospective cohort design. Most predictors (age group, sex, diagnoses of T2DM, hypertension, HIV, malignant neoplasms, neurological issues, urological issues, previous UTI diagnosis, immobilisation, DM with nephropathy) are administrative or diagnostic codes recorded prior to or independently of CAUTI ascertainment. Retrospective extraction from a curated database is unlikely to introduce systematic outcome-informed predictor recording. However, some predictors — notably ICU stay ≥10 days and hospital stay ≥4 days — are time-varying variables that accumulate during the same admission in which CAUTI is ascertained, introducing potential concurrent measurement issues. |
| D2.3 | Are all predictors available at the time the model is intended to be used? | PN | The model is described as fitting into two time points of clinical workflow: when a clinician encounters a patient, and when a clinician encounters a patient with urinary catheterisation already in place. The duration of catheterisation as a candidate predictor was defined as days from the start of urinary catheterisation to the day of prediction — this is a time-varying predictor that changes each day of the episode and is by design not a fixed baseline value. Furthermore, hospital stay ≥4 days is similarly time-varying. The model is therefore designed as a daily repeated-measures prediction tool rather than a single-point admission prediction model, which is an important design distinction. While this daily prediction approach is methodologically defensible, it means the model is not applicable at the time of catheter insertion alone, and the intended prediction time point differs fundamentally from the single admission-time models developed in other included reports. This distinction must be made explicit in the systematic review's comparison of prediction time points. |

**Domain 2 overall RoB: L**

Rationale: D2.1 and D2.2 are satisfactory for a large electronic health record database study. D2.3 is rated PN but the use of time-varying predictors in a daily repeated-measures design is a deliberate and methodologically coherent modelling choice rather than an inadvertent temporal leakage problem. The overall domain is rated Low because the predictor measurement approach is internally consistent with the model's stated design, though the D2.3 finding has important implications for cross-study comparison.

**Domain 2 Applicability: H**

Some health care-associated and behavioural variables, such as the compliance with the bundle care of urinary catheters and the drainage system, were not available in the database. More importantly, the daily repeated-measures prediction framework differs fundamentally from single admission-time prediction models used in most other included reports, limiting comparability. The 14 final predictors are all administrative or diagnostic codes that may not be uniformly available in electronic health record systems outside large academic hospitals, particularly in lower-resource settings.

**Domain 3 — Outcome**

|  | **Signalling Question** | **Response** | **Justification** |
| --- | --- | --- | --- |
| D3.1 | Was the outcome determined appropriately? | PN | A case of CAUTI was identified if (1) a diagnosis of UTI or an order for a urinary bacterial culture was recorded, and (2) there was no concurrent diagnosis of pneumonia. This is an administrative claims-based surrogate definition rather than a microbiologically confirmed CAUTI using standard clinical criteria. The definition relies on ICD diagnostic codes and order entries, without requiring a positive urine culture result, a specific bacterial count threshold, or clinical symptoms attributable to the urinary tract. The exclusion of concurrent pneumonia diagnosis is not explained and does not correspond to any standard CAUTI definition. This definition is likely to misclassify some cases — both over-identifying (UTI codes without confirmed infection) and under-identifying (confirmed CAUTI without a recorded UTI code). |
| D3.2 | Was a pre-specified or standard outcome definition used? | N | The outcome definition used — administrative UTI diagnosis code or urine culture order without concurrent pneumonia — does not correspond to any recognised standard CAUTI definition, including the NHSN/CDC, IDSA 2009, or ESCMID criteria. No standard guideline is cited for the outcome definition. The definition is operationally derived from available administrative data rather than pre-specified clinical criteria. This is a fundamental concern in a prediction model study, as the outcome being predicted does not correspond to the clinically defined entity that the model is presented as targeting. |
| D3.3 | Were predictors excluded from the outcome definition? | Y | The outcome is defined by administrative diagnostic codes and culture order entries. None of the 14 final predictors (age, sex, comorbidity codes, ICU stay, hospital stay, previous UTI history) are components of the outcome definition itself, though hospital stay and ICU stay are correlated with the likelihood of outcome ascertainment. |
| D3.4 | Was the outcome assessed without knowledge of predictor values? | Y | In an electronic health record database study, CAUTI ascertainment (administrative codes and culture orders) is recorded by clinical and administrative staff as part of routine care, independent of any research predictor extraction. The retrospective extraction process does not introduce knowledge of predictor values into outcome determination. |
| D3.5 | Was the time interval between predictor assessment and outcome determination appropriate? | PY | The model predicts CAUTI onset within a 3-day span over a 15-day range, assessed daily during the catheterisation episode. The duration of catheterisation predictor is defined as days from catheter start to the day of prediction, and outcome onset is assessed prospectively from the prediction day. This forward-looking structure is methodologically sound for a daily prediction model. However, the 48-hour post-removal window for outcome ascertainment introduces some ambiguity about whether post-removal UTIs represent true CAUTI or early post-catheter UTI. |
| D3.6 | Was there a sufficient proportion of participants with the outcome? | Y | The training set contained 10,676 CAUTI-positive instances out of 81,010 total (13.18%), and the test set contained 4,074 CAUTI-positive instances out of 21,123 (19.29%). The absolute number of events is very large and fully adequate for all modelling approaches. The higher event rate in the test set compared to the development set is noted by the authors as a good challenge to model robustness. |

**Domain 3 overall RoB: H**

Rationale: D3.2 is rated N and D3.1 is rated PN due to the use of a non-standard, administratively derived outcome definition that does not correspond to any recognised CAUTI clinical guideline. This is the single most important methodological concern in this study. A prediction model for "CAUTI" that uses an administratively coded surrogate outcome may predict administrative coding behaviour and healthcare utilisation patterns rather than clinically confirmed CAUTI, and its reported performance metrics cannot be interpreted as evidence of ability to predict true CAUTI as defined by standard criteria.

**Domain 3 Applicability: H**

The administratively derived CAUTI definition differs substantially from the microbiologically and clinically confirmed definitions used in most other included reports and in standard clinical practice. The model's predictions may not translate to settings using NHSN/CDC or IDSA criteria for CAUTI diagnosis, fundamentally limiting cross-study comparability and real-world applicability.

**Domain 4 — Analysis**

|  | **Signalling Question** | **Response** | **Justification** |
| --- | --- | --- | --- |
| D4.1 | Were there an appropriate number of participants with the outcome? | Y | The training set contained 10,676 CAUTI-positive instances with 14 final predictors, yielding EPV > 700. This is vastly adequate for all five ML algorithms employed. No sample size concerns exist in this study. |
| D4.2 | Were continuous and categorical predictors handled appropriately? | Y | All 14 final predictors are categorical (binary or ordinal). The duration of catheterisation is treated as a categorical predictor (defined in days from catheter start to prediction day, binned into categorical groups for the nomogram). Cox proportional-hazards modelling was used for predictor selection, and multiple ML algorithms handle non-linearity and interaction terms inherently. No inappropriate dichotomisation of continuous variables is reported. The use of random forest and XGBoost naturally accommodates non-additive and non-linear relationships. |
| D4.3 | Were all enrolled participants included in the analysis? | PY | Before predictor selection, candidate predictors with unavailable data, perfect separation problems, or missing not at random patterns were excluded. Among the remaining predictors, missing values appear to be handled within the modelling pipeline. The total of 122,386 instances is derived from a clear flow diagram (Figure 1A) with explicit exclusion criteria. However, the handling of missing predictor values within included instances is not fully described for all predictors beyond the preliminary exclusion step. |
| D4.4 | Were missing data handled appropriately? | PY | Predictors with missing not at random patterns were excluded at the predictor selection stage rather than imputed. For remaining predictors with missing values, the study notes that some indicators include missing values in the baseline table, and the total percentages may not sum to 100% due to missing values. The random forest algorithm is capable of handling missing values through surrogate splits. However, no formal multiple imputation or sensitivity analysis for missing data is reported, and the proportion of missing data for each predictor is not fully disclosed. |
| D4.5 | Was selection of predictors based on univariable analysis avoided? | Y | Predictor selection employed Cox proportional-hazards modelling for both univariable and multivariable regression analyses, following principles of structural causal modelling and using variance inflation factor analogues to address collinearity. Multiple testing correction was applied using the Benjamini-Hochberg method. Crucially, the structural causal modelling framework means that predictor selection was guided by pre-specified causal assumptions about the data-generating process rather than purely data-driven univariable screening. This is a more principled approach than the two-stage univariable-then-multivariable screening used in most other included reports. |
| D4.6 | Were complexities in the data accounted for appropriately? | PY | The study used data from three hospitals across different time periods, with explicit partitioning: two hospitals for development and one for external validation. The 80:20 random split of the development set into training and validation sets is straightforward. The multi-site nature of the data provides implicit diversity. However, potential clustering effects within hospitals (e.g., ward-level infection control practices, physician-level catheter management), temporal trends across the 23-year database period (1998–2021), and potential within-patient clustering from multiple catheterisation episodes is not addressed. |
| D4.7 | Were relevant model performance measures evaluated appropriately? | Y | Model performance was evaluated using calibration (calibration plot, intercept, slope, Brier score), clinical utility (decision curve analysis), and discrimination (AUC-ROC) on both the internal validation set and the external test set. The best model was selected based on validation set performance across all three criteria. Four threshold-based metrics (sensitivity, specificity, PPV, NPV) were also reported for the test set. This is a comprehensive performance evaluation framework that substantially exceeds the reporting standards of most other included reports. |
| D4.8 | Were model overfitting and optimism accounted for? | Y | The best model was selected using the internal validation set (not the training set), and its robustness was confirmed on the external test set from a separate hospital. The use of a held-out validation set for model selection and a genuinely independent test set for final performance estimation substantially mitigates overfitting concerns. Bootstrap confidence intervals (30-time bootstrapping) were used for interval estimation of performance metrics. Regularisation was applied across multiple algorithms (ridge regression with C=0.01 to C=10; tree depth pruning; minimum sample constraints). The combination of regularisation, internal validation, and external validation represents the most rigorous overfitting control among all included reports. |
| D4.9 | Do predictors and their assigned weights correspond to the multivariable analysis results? | Y | The predictor selection results are reported in Table 2 with both unadjusted and adjusted hazard ratios, 95% CIs, and corrected P-values for all 14 final predictors. The random forest model's predictor importance is visualised through SHAP values at both population and individual levels. A nomogram was constructed following a published protocol for algorithm-agnostic nomograms, providing a transparent paper-based implementation of the RF model. |
| D4.10 | Was model performance evaluated in different subgroups of participants? | PN | No formal subgroup performance analyses by patient characteristics (age group, sex, primary diagnosis, hospital ward type, ICU vs non-ICU) are reported. Given the broad inclusion criteria (all hospitalised catheterised patients aged 20–75) and the known heterogeneity of CAUTI risk across patient subgroups, the absence of subgroup analyses is a missed opportunity. SHAP-based individual-level explainability is provided but does not constitute systematic subgroup performance evaluation. |
| D4.11 | Was model performance evaluated using calibration? | Y | Calibration was assessed using a calibration plot with regression line on the validation set, reporting intercept −0.193 (95% CI: −0.412, 0.027), slope 0.844 (95% CI: −0.490, 1.198), and Brier score 0.453 (95% CI: 0.452, 0.453). The calibration plot visually shows predicted probability distributions for CAUTI-positive and CAUTI-negative instances. Calibration intercept, slope, and Brier score together constitute a thorough quantitative calibration assessment that substantially exceeds the reporting standard of most other included reports. |
| D4.12 | Was model performance evaluated in an external validation dataset? | Y | External validation was performed using data from Taipei Municipal Wanfang hospital, which was entirely separate from the two development hospitals and constituted 20.85% of all instances (n = 21,123). The external test set had a higher CAUTI prevalence (19.29%) than the development set (13.26%), providing a genuine challenge to model robustness. This is one of only two reports among all included studies to conduct external validation. |

**Domain 4 overall RoB: L**

Rationale: The analytical approach is substantially more rigorous than all other included reports. Sample size is vastly adequate; predictor selection follows structural causal modelling principles with multiple testing correction; five ML algorithms are systematically compared; overfitting is controlled through regularisation, internal validation, and external validation; and performance is comprehensively evaluated using discrimination, calibration, and clinical utility metrics. The only notable gaps are the absence of subgroup analyses (D4.10) and incomplete description of missing data handling for individual predictors (D4.4), neither of which rises to the level of high risk in the context of the overall analytical framework.

**Overall Judgment Summary**

| Domain | Risk of Bias | Applicability |
| --- | --- | --- |
| D1 Participants | L | H |
| D2 Predictors | L | H |
| D3 Outcome | H | H |
| D4 Analysis | L | — |
| Overall | H | H |

**PROBAST Assessment: Zhu Rui 2024**

**Report:** Zhu R, Tian YL, Huang M, Chu MJ. Establishment of a risk prediction model for catheter-associated urinary tract infection in elderly critically ill patients. *J Clin Med Pract.* 2024; 28(24): 143–148.

**Domain 1 — Participants**

|  | **Signalling Question** | **Response** | **Justification** |
| --- | --- | --- | --- |
| D1.1 | Were appropriate data sources used? | Y | Data were drawn from the hospital electronic medical record information system of the geriatric ICU at Jiangsu Provincial People's Hospital, a major tertiary academic hospital. An institutional electronic record system is an appropriate and well-established data source for CAUTI prediction modelling in critically ill inpatients. |
| D1.2 | Were all inclusions and exclusions of participants appropriate? | PY | Inclusion criteria required age ≥65 years, CAUTI diagnosis per the 2010 Chinese Ministry of Health guideline, and APACHE II score ≥25. Exclusion criteria included urinary tract infection unrelated to catheterisation, death within 48 hours of ICU admission, and incomplete data. These criteria are clinically appropriate. However, the inclusion of catheter-associated asymptomatic bacteriuria (CAASB) in the infection group introduces diagnostic heterogeneity, as CAASB is not universally considered equivalent to symptomatic CAUTI in prediction modelling. |
| D1.3 | Were participants enrolled at a similar and relevant point in their disease or condition? | PY | All patients were enrolled at the time of catheter insertion in the geriatric ICU, and those who died within 48 hours of ICU admission were excluded. This ensures a minimum follow-up period and consistent enrolment point. However, the study spans ten years (January 2014 to June 2024), during which infection control practices and surveillance standards may have evolved substantially, potentially introducing temporal heterogeneity in both predictor measurement and outcome ascertainment. |
| D1.4 | Was the sample size appropriate? | PY | A total of 8,905 patients were included, of whom 114 developed CAUTI. Eleven variables with statistical significance in univariate analysis were entered into the multivariate logistic regression model using stepwise backward elimination, ultimately yielding 5 model variables: age, consciousness status, renal dysfunction, catheter dwell time, and number of catheter insertions. With 114 events and 11 candidate variables entered into the multivariable model, EPV = 10.4, which meets the commonly cited minimum threshold of 10 but remains below more contemporary recommendations of EPV ≥ 20 for stable logistic regression models. Relative to the final 5-variable model, EPV = 22.8, which is adequate. The large total sample size (8,905) is the most favourable characteristic of this study among all included reports. |

**Domain 1 overall RoB: L**

Rationale: D1.1–D1.3 are satisfactory or close to satisfactory. D1.4 is rated PY given the marginal but acceptable EPV relative to candidate variables, and adequate EPV relative to the final model. This is the only included report in which sample size concerns do not rise to the level of high risk, driven by the unusually large cohort of 8,905 patients accumulated over 10 years.

**Domain 1 Applicability: H**

The study was conducted at a single centre, and whether the constructed model can accurately perform using data from different healthcare systems across a wide geographic area requires further validation. Furthermore, the study population is restricted to elderly patients (age ≥65) with APACHE II score ≥25 in a geriatric ICU at a single tertiary hospital in Nanjing, Jiangsu province. This highly specific population (elderly critically ill only, single centre, geriatric ICU) limits direct applicability to general ICU, non-elderly, or mixed ward populations.

**Domain 2 — Predictors**

|  | **Signalling Question** | **Response** | **Justification** |
| --- | --- | --- | --- |
| D2.1 | Were predictors defined and assessed in a similar way for all participants? | PY | Retrospective collection included: general information such as age, sex, admission and discharge dates; primary diseases or comorbidities including stroke, cardiovascular disease, diabetes, liver dysfunction, renal dysfunction, and malignant tumours; ICU management including ICU length of stay, prophylactic antibiotic use, surgical and invasive procedures; and infection status including CAUTI occurrence, timing, and microbiological results. These are standard variables recorded in routine electronic medical records. The 10-year data collection period introduces potential for temporal inconsistency in documentation practices, but no systematic assessment of inter-period or inter-recorder reliability is reported. |
| D2.2 | Were predictor assessments made without knowledge of outcome data? | PY | Fixed or early-measured predictors (age, sex, comorbidities, consciousness status at admission) are objective clinical variables recorded independent of subsequent CAUTI status. Retrospective extraction from electronic records is unlikely to have been systematically influenced by knowledge of CAUTI outcome for these variables. However, some uncertainty exists for prophylactic antibiotic use and ICU length of stay, which reflect evolving clinical management decisions during the same admission in which CAUTI is ascertained. |
| D2.3 | Are all predictors available at the time the model is intended to be used? | PN | The model is intended for early identification of high-risk patients during ICU admission. The five final predictors in the nomogram are age, consciousness status, renal dysfunction, catheter dwell time, and number of catheter insertions. Of these, catheter dwell time (categorised as 1–6 d, 7–9 d, ≥10 d) and number of catheter insertions (1, 2, ≥3) are time-varying variables that accumulate over the course of hospitalisation and cannot be known at admission or catheter insertion. In the univariate analysis, 75.44% of the infection group had catheter dwell time ≥10 days versus only 19.65% in the non-infection group, and 39.47% of the infection group had ≥2 catheter insertions versus 18.20% in the non-infection group. These strong associations reflect post-event accumulation rather than true baseline predictors. Same temporal leakage issue as Liu Huiying 2025 and Li Mengchao 2025. |

**Domain 2 overall RoB: H**

Rationale: D2.3 is rated PN. Catheter dwell time and number of catheter insertions — two of the five final nomogram predictors — are fundamentally time-varying variables that cannot be known at the intended prediction time point (ICU admission or catheter insertion). This represents temporal leakage that undermines the model's stated clinical purpose of early risk identification, consistent with the same flaw identified in multiple other included reports.

**Domain 2 Applicability: L**

The predictors used (age, consciousness status, renal dysfunction, catheter dwell time, catheter insertion count) are standard clinical variables available in routine ICU care. No proprietary instruments or highly specialised measurements are required.

**Domain 3 — Outcome**

|  | **Signalling Question** | **Response** | **Justification** |
| --- | --- | --- | --- |
| D3.1 | Was the outcome determined appropriately? | PY | CAUTI diagnosis was made per the 2010 Chinese Ministry of Health "Technical Guidelines for Prevention and Control of Catheter-Associated Urinary Tract Infection (Trial)" and required clinical symptoms (urinary irritation symptoms, suprapubic tenderness, costovertebral angle pain, fever) combined with microbiological criteria: clean-catch midstream urine or catheter-collected urine culture with Gram-positive cocci ≥10⁴ CFU/mL or Gram-negative bacilli ≥10⁵ CFU/mL; or suprapubic bladder puncture urine culture ≥10³ CFU/mL; or fresh urine sediment showing bacteria in half of 30 microscopic fields. The use of a published national guideline with combined clinical and microbiological criteria is appropriate. |
| D3.2 | Was a pre-specified or standard outcome definition used? | PN | Although the 2010 Chinese Ministry of Health guideline is referenced, the outcome definition applied in this study includes CAASB (catheter-associated asymptomatic bacteriuria) in the infection group. For patients without clinical symptoms, those meeting the diagnostic criteria for catheter-associated asymptomatic bacteriuria (CAASB) — defined as urinary catheterisation or endoscopic examination within 1 week, with urine culture showing Gram-positive cocci ≥10⁴ CFU/mL or Gram-negative bacilli ≥10⁵ CFU/mL — were also included in the CAUTI infection group. The inclusion of asymptomatic bacteriuria cases alongside symptomatic CAUTI conflates two clinically distinct entities. Standard definitions (NHSN/CDC, IDSA) explicitly separate CAUTI from CAASB and treat them differently. This broadened definition may inflate the apparent CAUTI rate and introduce heterogeneity in the outcome, making the model's predictions difficult to interpret or apply in settings using standard symptomatic-only CAUTI definitions. |
| D3.3 | Were predictors excluded from the outcome definition? | Y | The CAUTI/CAASB outcome is defined by microbiological culture results and clinical symptoms. None of the five final model predictors (age, consciousness status, renal dysfunction, catheter dwell time, catheter insertions) are components of the outcome definition. |
| D3.4 | Was the outcome assessed without knowledge of predictor values? | U | No information is provided on whether clinicians or infection control staff who ascertained CAUTI/CAASB status were blinded to predictor values. In a retrospective electronic record study spanning 10 years, formal blinding is not performed and cannot be confirmed. |
| D3.5 | Was the time interval between predictor assessment and outcome determination appropriate? | PN | As noted in D2.3, catheter dwell time and number of catheter insertions are measured concurrently with, or as cumulative consequences of, the hospitalisation period during which CAUTI is being ascertained. The temporal separation between predictor measurement and outcome determination is not cleanly established for these two variables. |
| D3.6 | Was there a sufficient proportion of participants with the outcome? | PY | Among 8,905 patients, 114 developed CAUTI, with a CAUTI incidence rate of 2.55‰ (calculated as CAUTI cases / total catheter dwell days × 1,000‰). The absolute number of 114 events is adequate for logistic regression model development, particularly relative to the 11 candidate variables (EPV = 10.4). The event rate of 1.28% (114/8905) is low but the large total sample compensates for this. |

**Domain 3 overall RoB: H**

Rationale: D3.2 is rated PN due to the non-standard inclusion of CAASB cases in the CAUTI outcome group, which conflates two clinically distinct entities and deviates from NHSN/CDC and IDSA definitions. D3.5 is rated PN due to concurrent measurement of key predictors and the outcome. These two issues together raise the domain to high risk.

**Domain 3 Applicability: H**

The inclusion of CAASB in the outcome definition differs from standard definitions used in most other included reports and in international clinical practice. Results may not be directly comparable to studies or clinical settings applying symptomatic-only CAUTI definitions, and the model may predict a combined symptomatic/asymptomatic endpoint rather than clinically relevant CAUTI.

**Domain 4 — Analysis**

|  | **Signalling Question** | **Response** | **Justification** |
| --- | --- | --- | --- |
| D4.1 | Were there an appropriate number of participants with the outcome? | PY | With 114 events and 11 candidate variables, EPV = 10.4, marginally meeting the commonly cited threshold of 10. Relative to the final 5-variable model, EPV = 22.8. While not ideal by contemporary standards (EPV ≥ 20 for candidate variables), this is the most favourable EPV among all included reports except Sufriyana 2025. |
| D4.2 | Were continuous and categorical predictors handled appropriately? | PN | Age was categorised into three ordinal groups (65–74, 75–84, ≥85). Consciousness status was treated as an ordinal variable (alert, drowsy, stupor, coma, dementia). Catheter dwell time was categorised (1–6 d, 7–9 d, ≥10 d). Number of catheter insertions was categorised (1, 2, ≥3). All continuous variables were categorised or dichotomised, with cut-points that appear data-driven rather than pre-specified based on clinical or external evidence. No assessment of linearity on the log-odds scale for any continuous variable is reported. Categorisation of continuous predictors inflates apparent model performance and produces threshold instability. |
| D4.3 | Were all enrolled participants included in the analysis? | PN | Patients with incomplete medical records and unavailable data were excluded. The number of patients excluded for incomplete data is not reported. It is therefore impossible to assess whether this exclusion introduced selection bias or whether missingness was related to outcome severity. |
| D4.4 | Were missing data handled appropriately? | N | As noted in D4.3, patients with incomplete data were excluded without any description of the missing data mechanism, the number affected, or sensitivity analysis. No imputation strategy is reported. Complete-case analysis without justification is inappropriate when the missingness mechanism is unknown. |
| D4.5 | Was selection of predictors based on univariable analysis avoided? | N | Eleven variables with statistical significance in univariate analysis were entered into the multivariate logistic regression model using stepwise backward elimination to identify independent risk factors for CAUTI. This two-stage approach — univariable screening followed by multivariable stepwise selection — is a well-recognised source of optimism and predictor selection instability. Stepwise methods are additionally problematic as they use the same data for both variable selection and parameter estimation, inflating apparent precision. This is explicitly flagged as methodologically suboptimal by contemporary prediction modelling guidelines. |
| D4.6 | Were complexities in the data accounted for appropriately? | U | No information is provided on whether clustering effects (e.g., admission period, ward, treating team) or temporal trends across the 10-year study period are addressed. The 10-year span (2014–2024) is the longest among all included reports and covers substantial changes in ICU care practices, antibiotic stewardship, and catheter bundle implementation in China. Potential within-period correlation and secular trends are not modelled or discussed. |
| D4.7 | Were relevant model performance measures evaluated appropriately? | PY | The clinical prediction model ROC curve AUC was 0.802 (95% CI: 0.796–0.809), demonstrating good discrimination between positive (CAUTI) and negative (no CAUTI) patients. The calibration curve showed the risk prediction nomogram calibration curve was close to the ideal curve. The Hosmer-Lemeshow goodness-of-fit test showed χ²=8.525, P=0.581, indicating the prediction model has high calibration accuracy. AUC, calibration curve, and Hosmer-Lemeshow test together constitute a reasonable performance evaluation. However, Brier score, calibration slope, calibration intercept, and decision curve analysis are absent. |
| D4.8 | Were model overfitting and optimism accounted for? | PN | Bootstrap resampling with 5,000 iterations was used to draw the ROC curve and calculate AUC for internal validation of the model's discrimination; Bootstrap resampling with 5,000 iterations was also used to draw the calibration curve and perform the Hosmer-Lemeshow test for internal validation of calibration. Bootstrap resampling was applied to both ROC and calibration curve construction, which is methodologically appropriate. However, it is not stated whether optimism-corrected (shrinkage-adjusted) performance estimates are reported, or whether the bootstrap was used solely for confidence interval estimation and curve plotting. Given the use of stepwise variable selection and data-driven cut-points, residual optimism in the reported AUC of 0.802 remains likely even with bootstrap resampling. |
| D4.9 | Do predictors and their assigned weights correspond to the multivariable analysis results? | Y | Regression coefficients (B), standard errors SE(B), Wald χ², odds ratios, 95% CIs, and P-values are reported for all variables in the multivariate logistic regression model in Table 3. Age, consciousness status, renal dysfunction, catheter dwell time, and catheter insertion count were incorporated as final risk prediction factors in the logistic regression equation and developed into a nomogram. The nomogram is constructed directly from the multivariable regression results and is internally consistent. |
| D4.10 | Was model performance evaluated in different subgroups of participants? | N | No subgroup performance analyses are reported. Given that the study covers 10 years and includes patients from a geriatric ICU with diverse comorbidities (stroke, cardiovascular disease, malignancy), the absence of subgroup analyses by time period, primary diagnosis, or comorbidity burden is a notable omission. |
| D4.11 | Was model performance evaluated using calibration? | PY | The calibration curve was close to the standard curve, and the Hosmer-Lemeshow goodness-of-fit test result was χ²=8.525, P=0.581, indicating the prediction model has high calibration accuracy. Calibration curve and Hosmer-Lemeshow test are reported. However, the degrees of freedom for the H-L test are not reported, calibration slope and intercept are absent, and Brier score is not provided. The H-L test P-value alone (0.581) is insufficient to fully characterise calibration across the probability range. |
| D4.12 | Was model performance evaluated in an external validation dataset? | N | The study's limitations include that whether the constructed model can accurately perform using data from different healthcare systems across a wide geographic area still requires further validation, and the model should be further tested in different populations with various medical practices. All reported performance is from the derivation dataset using bootstrap internal validation only. No external validation in an independent cohort has been performed. |

**Domain 4 overall RoB: H**

Rationale: Multiple significant analytical problems: univariable pre-screening with stepwise selection for predictor identification; data-driven categorisation of all continuous variables without linearity testing; complete-case analysis without missing data handling; no external validation; and incomplete calibration reporting. Although the bootstrap approach and the relatively adequate EPV are positive features, these do not offset the identified methodological deficiencies.

**Overall Judgment Summary**

| Domain | Risk of Bias | Applicability |
| --- | --- | --- |
| D1 Participants | L | H |
| D2 Predictors | H | L |
| D3 Outcome | H | H |
| D4 Analysis | H | — |
| Overall | H | H |

**PROBAST Assessment: Sun Qing 2024**

**Report:** Sun Q, Mao QY. Construction and validation of a nomogram model for risk prediction of catheter-associated urinary tract infection in a tertiary hospital. Master's Thesis, Shandong First Medical University. 2024.

**Domain 1 — Participants**

|  | **Signalling Question** | **Response** | **Justification** |
| --- | --- | --- | --- |
| D1.1 | Were appropriate data sources used? | Y | Retrospective clinical and epidemiological data were extracted from the hospital electronic medical record system and real-time nosocomial infection surveillance system of a tertiary hospital in China. These routine institutional data sources are appropriate for identifying indwelling catheter patients and confirming CAUTI events in the inpatient population. |
| D1.2 | Were all inclusions and exclusions of participants appropriate? | PY | Eligibility criteria are clinically appropriate: cases met the standard CAUTI diagnosis with indwelling urinary catheter; controls were 1:1 matched by admission timing, age group, department, disease diagnosis and sex, with indwelling catheter but no CAUTI. Exclusions included pre-admission catheterisation, pre-existing UTI before catheterisation, and severely incomplete clinical records. However, the number and proportion of patients excluded due to incomplete data are not reported, introducing uncertainty about potential selection bias. The matched case-control design also restricts control selection, which may limit the representativeness of the control group. |
| D1.3 | Were participants enrolled at a similar and relevant point in their disease or condition? | Y | Both cases and controls were drawn from hospitalised patients undergoing indwelling urinary catheterisation. Matching by admission time, department and disease type ensures that participants entered the risk period at a comparable clinical baseline. No prevalent cohort bias is identified. |
| D1.4 | Was the sample size appropriate? | PN | The study included 521 matched pairs (1042 total participants) with 521 CAUTI events. With 4 final predictors in the model, the events per variable (EPV) ratio is 130.3, which substantially exceeds the conventional minimum threshold of 20. Even when accounting for the 10 candidate variables entered into the multivariable model, the candidate EPV remains 52.1. However, no *a priori* sample size calculation was reported. |

**Domain 1 overall RoB: H**

**Rationale:** D1.2 is rated PY due to unquantified exclusions for incomplete data and potential selection bias from the matched design. D1.4 is rated PN due to the absence of a pre-specified sample size calculation and the distortion of baseline risk by the 1:1 matching design. Although the absolute event count is large, these limitations together place the domain at high risk of bias.

**Domain 1 Applicability: H**

The study was conducted at a single tertiary hospital in China using a matched case-control design. The patient population spans multiple clinical departments but is limited to one institution. The 1:1 matching further reduces the generalisability of absolute risk estimates to unselected catheterised inpatient populations. Multicentre external validation is required before the model can be applied broadly.

**Domain 2 — Predictors**

|  | **Signalling Question** | **Response** | **Justification** |
| --- | --- | --- | --- |
| D2.1 | Were predictors defined and assessed in a similar way for all participants? | PY | Eighteen candidate predictors covering patient demographics, comorbidities and treatment-related factors were collected retrospectively from medical records using a standardised data collection form. A table of variable definitions is provided. However, no formal data dictionary, inter-rater reliability testing, or detailed description of data abstraction protocols is provided. Cut-off points for dichotomised continuous variables were described as "optimal cut-off values combined with clinical practice", but the method for determining these thresholds is not specified. |
| D2.2 | Were predictor assessments made without knowledge of outcome data? | PY | Baseline comorbidities such as nervous system disease and diabetes were recorded at admission, prior to CAUTI onset. However, time-varying predictors including total catheter duration, ICU length of stay, and number of antibiotic types are cumulative exposures measured over the entire admission. In this retrospective study, these variables were abstracted after CAUTI status was known, creating potential for differential ascertainment bias. |
| D2.3 | Are all predictors available at the time the model is intended to be used? | N | Only one of the four final independent predictors — nervous system disease — is known at the time of catheter insertion or hospital admission. The remaining three predictors (catheter days ≥7, ICU stay ≥7, and ≥3 types of antibiotics) are all time-varying, cumulative exposures that cannot be determined at the point when risk stratification would guide preventive interventions. Three of four final predictors are unavailable at catheter insertion; the model cannot function as an admission-time risk stratification tool. |

**Domain 2 overall RoB: H**

**Rationale:** D2.3 is rated N. Three out of four final predictors are time-varying variables that are unknowable at the clinically relevant time point of catheter insertion. This represents extensive temporal leakage that fundamentally undermines the model's purpose as a predictive tool for early intervention. D2.1 and D2.2 are rated PY due to incomplete reporting of data collection methods and retrospective ascertainment of time-varying predictors.

**Domain 2 Applicability: L**

All included predictors are routinely documented variables available in standard electronic medical records. No specialised laboratory tests, proprietary instruments or complex scoring systems are required. Nervous system disease, ICU admission status and antibiotic prescribing information are all readily accessible to clinical and infection control staff.

**Domain 3 — Outcome**

|  | **Signalling Question** | **Response** | **Justification** |
| --- | --- | --- | --- |
| D3.1 | Was the outcome determined appropriately? | PY | CAUTI was diagnosed according to the 2010 Chinese Ministry of Health *Guidelines for Prevention and Control of Catheter-associated Urinary Tract Infection (Trial)*, which requires both compatible clinical symptoms and confirmatory urine culture results. Cases were jointly confirmed by infection control specialists and clinicians and reported through the hospital surveillance system. However, the specific symptom criteria and exact colony count thresholds applied are not explicitly enumerated in the text, preventing full verification of diagnostic adherence. |
| D3.2 | Was a pre-specified or standard outcome definition used? | Y | The study adopted the nationally standardised 2010 Chinese Ministry of Health CAUTI diagnostic criteria, which is a well-established, pre-specified definition widely used in hospital infection surveillance and research across China. |
| D3.3 | Were predictors excluded from the outcome definition? | Y | The CAUTI outcome is defined by clinical manifestations and microbiological evidence. None of the four final predictors (catheter duration, ICU stay, nervous system disease, number of antibiotic types) are included as components of the diagnostic definition. |
| D3.4 | Was the outcome assessed without knowledge of predictor values? | U | No information is provided on whether clinicians or infection control staff who confirmed CAUTI diagnoses were blinded to predictor values such as catheter duration or antibiotic exposure. Blinding cannot be confirmed in this retrospective study design. |
| D3.5 | Was the time interval between predictor assessment and outcome determination appropriate? | N | Catheter duration ≥7 days and ICU stay ≥7 days are cumulative exposures measured over the same period in which CAUTI is ascertained; in patients who develop CAUTI, these values reflect exposure accumulated up to and including the infection event, inflating their apparent association with the outcome. The number of antibiotic types also accumulates during the same risk period. This concurrent measurement creates severe temporal confounding and inflates the apparent predictive strength of these variables. |
| D3.6 | Was there a sufficient proportion of participants with the outcome? | Y | The 1:1 matched design yielded 521 CAUTI events, providing a large absolute number of outcomes for statistical modelling. While the 50% event proportion in the analysis sample does not reflect the low population incidence, the absolute event count is more than adequate for logistic regression analysis. |

**Domain 3 overall RoB: H**

**Rationale:** D3.5 is rated N — two of four final predictors are measured concurrently with outcome ascertainment, resulting in substantial temporal confounding. D3.1 is rated PY due to incomplete specification of diagnostic criteria. D3.4 is rated Unclear. Combined, these issues place the outcome domain at high risk of bias.

**Domain 3 Applicability: L**

The 2010 Chinese Ministry of Health CAUTI definition is the national standard for hospital infection surveillance in China. The outcome is directly applicable to routine clinical practice, infection control auditing and quality improvement programmes in Chinese healthcare settings.

**Domain 4 — Analysis**

|  | **Signalling Question** | **Response** | **Justification** |
| --- | --- | --- | --- |
| D4.1 | Were there an appropriate number of participants with the outcome? | PN | With 521 CAUTI events and 4 final predictors, the EPV is 130.3, which well exceeds the conventional minimum of 20. Even with 10 candidate variables entered into multivariable analysis, the candidate EPV remains 52.1. However, no *a priori* sample size calculation was performed. The matched case-control design also distorts baseline risk, which affects the calibration of absolute probability estimates. |
| D4.2 | Were continuous and categorical predictors handled appropriately? | N | All continuous predictors including catheter duration, ICU stay and hospital stay were dichotomised into binary variables based on unspecified optimal cut-offs. No assessment of linearity on the log-odds scale was conducted. Dichotomisation of continuous variables discards statistical information, reduces model power, and introduces threshold-dependent instability. No justification for the selected cut-points is provided beyond general clinical convention. |
| D4.3 | Were all enrolled participants included in the analysis? | PY | All 521 matched pairs were included in the final analysis with no further exclusions after matching. However, the number of eligible patients excluded before matching due to incomplete clinical data is not reported, so the completeness of follow-up of the original eligible cohort cannot be confirmed. |
| D4.4 | Were missing data handled appropriately? | U | Patients with severely incomplete clinical data were excluded as an eligibility criterion. The proportion of excluded patients, the pattern of missingness, and the variables most affected by missing data are not described. No multiple imputation or sensitivity analysis for missing data was performed. The impact of missing data on model results cannot be determined. |
| D4.5 | Was selection of predictors based on univariable analysis avoided? | N | Variable selection followed a two-stage procedure: variables with *P*<0.05 in univariate analysis were entered into the multivariate model, with additional variables showing *P*<0.1 also included for clinical relevance. This univariable pre-screening approach is a well-recognised source of model overfitting, selection bias and inflated effect estimates, consistent with methodology commonly used in similar single-centre CAUTI prediction studies. |
| D4.6 | Were complexities in the data accounted for appropriately? | PN | The study appropriately used conditional logistic regression to account for the 1:1 matched case-control design. However, the analysis does not address potential clustering by clinical department, temporal trends over the 4-year study period (2017–2021), or changes in infection control practice during the COVID-19 pandemic period covered by the study. |
| D4.7 | Were relevant model performance measures evaluated appropriately? | PN | The study evaluated discrimination via ROC curve (AUC = 0.677), calibration via calibration curve, and clinical utility via decision curve analysis and clinical impact curve. However, 95% confidence intervals for the AUC are not explicitly reported. No quantitative calibration metrics such as Hosmer-Lemeshow test statistics, Brier score, calibration slope or calibration intercept are provided, relying instead on qualitative descriptions of calibration performance. |
| D4.8 | Were model overfitting and optimism accounted for? | PN | Internal validation was performed using bootstrap resampling with 1000 replications, which is an appropriate method to assess model optimism. However, no penalised regression (e.g., LASSO) or coefficient shrinkage was applied to adjust for overfitting. No external validation in an independent cohort was conducted. The relatively modest AUC (0.677) may indicate less severe overfitting than studies with very high AUC values, but optimism-adjusted performance estimates are not explicitly presented. |
| D4.9 | Do predictors and their assigned weights correspond to the multivariable analysis results? | Y | Multivariate logistic regression results including β coefficients, OR values and 95% CIs are tabulated for all four final predictors. The nomogram is constructed directly from these regression coefficients, and the relative variable weights in the nomogram correspond consistently with the magnitude of the reported β estimates. |
| D4.10 | Was model performance evaluated in different subgroups of participants? | N | No subgroup analyses of model discrimination or calibration are reported. The study population spans multiple departments with widely varying baseline CAUTI risk (from 0.144‰ to 7.494‰), but model performance across these clinical subgroups is not evaluated. |
| D4.11 | Was model performance evaluated using calibration? | PN | A calibration curve is presented, and the study states that predicted probabilities correspond well with observed probabilities. Bootstrap internal validation was also applied to calibration assessment. However, no quantitative calibration statistics are reported, including Hosmer-Lemeshow test *P*-value, calibration slope, Brier score or mean absolute error, preventing formal assessment of calibration quality. |
| D4.12 | Was model performance evaluated in an external validation dataset? | N | Only internal bootstrap validation was performed. No external validation using an independent patient cohort from a different hospital, time period or clinical setting was conducted. The transportability and generalisability of the model remain untested. |

**Domain 4 overall RoB: H**

**Rationale:** Critical analytical limitations include univariable pre-screening for variable selection (D4.5 = N), inappropriate dichotomisation of all continuous predictors (D4.2 = N), absence of external validation (D4.12 = N), and incomplete quantitative reporting of calibration and discrimination metrics (D4.7, D4.11 = PN). Relative strengths include appropriate use of conditional logistic regression for matched data and bootstrap internal validation, but these do not mitigate the fundamental methodological biases.

**Overall Judgment Summary**

| Domain | Risk of Bias | Applicability |
| --- | --- | --- |
| D1 Participants | H | H |
| D2 Predictors | H | L |
| D3 Outcome | H | L |
| D4 Analysis | H | — |
| Overall | H | H |

**PROBAST Assessment: Wang Fang 2023**

**Report:** Wang F, Wang X, Shi Y, et al. Development of a risk nomogram predicting urinary tract infection in patients with indwelling urinary catheter after radical surgery for cervical cancer. *Progrès en urologie.* 2023; 33: 492–502.

**Domain 1 — Participants**

|  | **Signalling Question** | **Response** | **Justification** |
| --- | --- | --- | --- |
| D1.1 | Were appropriate data sources used? | Y | Clinical records from 380 patients undergoing radical hysterectomy at a single tertiary military hospital; appropriate for a post-operative UTI prediction model in this surgical population. |
| D1.2 | Were all inclusions and exclusions of participants appropriate? | PY | The criteria for inclusion were: aged ≥18 years, normal intelligence, and cervical squamous cell carcinomas or adenocarcinoma diagnosed by cervical histological biopsy. The surgical methods were laparoscopic vaginal and abdominal RH. All patients underwent type C radical hysterectomy based on Querleu-Morrow classification. The exclusion criteria include patients with preoperative indwelling catheters, intraoperative urinary tract injuries, kidney diseases, kidney transplants, and postoperative intermittent catheterization. These criteria are clinically appropriate and effectively restrict the population to patients genuinely at risk of post-operative catheter-associated UTI. However, the exclusion of patients with incomplete medical records is noted without reporting the number excluded or whether missingness was related to outcome. |
| D1.3 | Were participants enrolled at a similar and relevant point in their disease or condition? | Y | All patients possess comprehensive medical records. The complete medical record included a complete postoperative review and follow-up records. Follow-up records continued until the patient had <100 mL of residual bladder urine after catheter removal and did not require continued indwelling catheterization. Enrolment occurs at the time of radical hysterectomy with postoperative catheter insertion, which is the appropriate and consistent time point for this specific prediction model. The exclusion of patients with preoperative indwelling catheters ensures that all participants begin the risk period at the same point. |
| D1.4 | Was the sample size appropriate? | PN | Following the inclusion and exclusion criteria, 380 patients were finally included in the training set. Patients were classified as infected and non-infected groups. From Table 1, 88 patients developed UTI (23.2% event rate). Among the 19 features of the 380 patients in the cohort, five predictors with non-zero LASSO regression coefficients were firstly chosen, which included age, history of UTIs, whether it is secondary catheterization, indwelling catheterization duration, and history of preoperative chemotherapy. LASSO was applied to 19 candidate variables. With 88 events and 19 candidate variables, EPV = 4.6, which is critically below the accepted threshold of 10 and substantially below the contemporary recommendation of EPV ≥ 20 for stable logistic regression model development. No formal sample size calculation is reported. |

**Domain 1 overall RoB: H**

Rationale: D1.4 is rated PN due to a critically low EPV of 4.6 (88 events, 19 candidate variables). D1.1–D1.3 are satisfactory. As with all previously assessed reports, the high-risk judgment is driven by sample size inadequacy relative to the number of candidate variables, not by retrospective design per se.

**Domain 1 Applicability: H**

For CC patients with stage IB to IIA, radical hysterectomy and pelvic lymphadenectomy is one of the main treatments for cervical cancer. The study population is exclusively female patients with cervical cancer (squamous cell carcinoma or adenocarcinoma, stages Ib–IIb) undergoing type C radical hysterectomy at a single tertiary military hospital in Chongqing. This is a highly specific surgical oncology population at a single centre. The model's applicability is restricted to post-radical hysterectomy patients with cervical cancer and may not generalise to other catheterised surgical patients, non-oncological patients, or multi-centre settings.

**Domain 2 — Predictors**

|  | **Signalling Question** | **Response** | **Justification** |
| --- | --- | --- | --- |
| D2.1 | Were predictors defined and assessed in a similar way for all participants? | PY | This study adopted a self-designed general information survey form, which included personal factors, disease factors, and treatment-related factors. The personal factors include age, BMI, education level, and place of residence. The disease factors included histological biopsy, staging of cervical cancer, method of operation, hospitalization days, operation time, history of UTI, the medical record of diabetes, hypertension history, history of urinary retention, whether it is secondary catheterization and indwelling catheterization duration. The treatment-related factors included hypoalbuminemia or anemia, preoperative chemotherapy, the number of antibiotics used, and days of antibiotics used. Data were entered by two people and double-checked. The use of a standardised self-designed survey form with dual data entry is a positive feature supporting measurement consistency. However, no formal data dictionary, inter-rater reliability assessment, or validation of the survey form is reported. |
| D2.2 | Were predictor assessments made without knowledge of outcome data? | PY | Most predictors (age, BMI, education, place of residence, cancer stage, histology, comorbidities, history of UTI, history of urinary retention) are fixed characteristics recorded at or prior to surgery, independent of subsequent UTI development. Retrospective extraction of these values from medical records is unlikely to have been systematically influenced by knowledge of UTI outcome. However, some predictors — hospitalization days, days of antibiotics used, number of antibiotics used, and indwelling catheterization duration — are time-varying variables that accumulate during the same admission in which UTI is being ascertained, introducing potential concurrent measurement issues. |
| D2.3 | Are all predictors available at the time the model is intended to be used? | PN | A prognostic nomogram has been developed with good accuracy which can help physicians to assess the risk of UTI in patients with ICa-RCCS at the early stage of treatment. Based on individualized risk assessment, physicians and patients could take more measures in diagnosis, treatment, and prevention. The model is therefore intended for use at the early stage of treatment — implying use at or near the time of surgery or catheter insertion. However, LASSO regression analysis suggested that indwelling catheterization duration ≥14 days and catheterization ≥twice were independent risk factors of UTIs in patients with ICa-RCCS. Indwelling catheterization duration is a time-varying variable that cannot be known at the time of catheter insertion. In Table 1, 64.8% of infected patients had catheter duration >28 days versus only 24.3% of non-infected patients — a difference that primarily reflects post-event accumulation rather than a baseline predictor. Whether secondary catheterization is similarly a variable that in part reflects evolving post-operative bladder dysfunction and may not be fully knowable at the point of first catheter insertion. These constitute temporal leakage for a model intended for early-stage use. |

**Domain 2 overall RoB: H**

Rationale: D2.3 is rated PN. Indwelling catheterization duration — the single most influential predictor in the nomogram (carrying the highest coefficient weight in Figure 2) — is fundamentally a time-varying variable that accumulates over the post-operative period and cannot be known at the time of intended model use (early stage of treatment / catheter insertion).

**Domain 2 Applicability: L**

The predictors used (age, history of UTI, secondary catheterization, catheterization duration, preoperative chemotherapy) are standard clinical variables routinely recorded in gynaecological oncology settings. No proprietary instruments or highly specialised measurements are required, and the variables are clinically intuitive for practitioners managing post-radical hysterectomy care.

**Domain 3 — Outcome**

|  | **Signalling Question** | **Response** | **Justification** |
| --- | --- | --- | --- |
| D3.1 | Was the outcome determined appropriately? | PY | CAUTI is defined as the presence of UTI-related symptoms or signs, and no other identified source of infection, as well as bacterial load ≥10³ cfu/mL in single catheter urine or in a midstream voided urine specimen from a patient catheter was removed within the past 48 hours. The definition requires both clinical symptoms/signs and microbiological confirmation, which is appropriate. The bacterial threshold of ≥10³ CFU/mL is consistent with the 2009 IDSA guideline for symptomatic CAUTI. |
| D3.2 | Was a pre-specified or standard outcome definition used? | PY | The CAUTI definition applied (symptoms/signs plus ≥10³ CFU/mL) is consistent with the 2009 IDSA guideline cited as reference [15] in this paper. The inclusion of the 48-hour post-removal window is also consistent with standard CAUTI definitions. However, the specific symptoms or signs used to confirm UTI are not enumerated (e.g., fever threshold, dysuria, suprapubic tenderness), making full verification of adherence to the standard impossible. The definition is nonetheless substantially more complete than those used in Liu 2023 or Liu Huiying 2025. |
| D3.3 | Were predictors excluded from the outcome definition? | Y | The UTI outcome is defined by microbiological culture and clinical signs/symptoms. None of the five final nomogram predictors (age, history of UTI, secondary catheterization, catheterization duration, preoperative chemotherapy) are components of the outcome definition. |
| D3.4 | Was the outcome assessed without knowledge of predictor values? | U | No information is provided on whether clinicians or infection control staff who ascertained UTI status were blinded to predictor values. In a retrospective medical record study, formal blinding is not performed and cannot be confirmed. |
| D3.5 | Was the time interval between predictor assessment and outcome determination appropriate? | PN | As noted in D2.3, indwelling catheterization duration is measured over the same period during which UTI is being ascertained. The relationship between catheter duration and UTI outcome is therefore partially concurrent rather than purely prospective. The "whether secondary catheterization" variable similarly reflects evolving post-operative bladder management during the follow-up period. The temporal separation between predictor assessment and outcome determination is not cleanly established for these two predictors. |
| D3.6 | Was there a sufficient proportion of participants with the outcome? | PY | Of 380 patients, 88 developed UTI, an event rate of 23.2%. CAUTI's incidence is about 5 to 18% in this population according to the literature cited, and the observed 23.2% is slightly above the expected range, though within the plausible upper range for this high-risk surgical population. The absolute number of 88 events is the largest among all included reports in this series, and is marginally adequate for modelling, though EPV remains low relative to the 19 candidate variables (see D1.4). |

**Domain 3 overall RoB: H**

Rationale: D3.5 is rated PN due to concurrent measurement of the key predictor (catheterization duration) and the outcome over the same hospitalisation period. D3.4 cannot be confirmed. D3.2 is rated PY — the outcome definition is the most complete and guideline-concordant among all included reports evaluated to date, which is a relative strength of this study.

**Domain 3 Applicability: L**

The UTI definition used (symptoms/signs plus ≥10³ CFU/mL, based on the 2009 IDSA guideline) is consistent with internationally recognised standards and broadly comparable to definitions used in other included reports. The post-removal 48-hour window is standard. Applicability concern

is low for the outcome domain.

**Domain 4 — Analysis**

|  | **Signalling Question** | **Response** | **Justification** |
| --- | --- | --- | --- |
| D4.1 | Were there an appropriate number of participants with the outcome? | PN | With 88 UTI events and 19 candidate variables submitted to LASSO, EPV = 4.6. Even relative to the five final predictors selected by LASSO (EPV = 88/5 = 17.6), the candidate EPV of 4.6 is the more relevant metric as LASSO itself is a data-driven selection procedure. Contemporary guidance recommends EPV ≥ 20 for stable logistic regression, and EPV ≥ 100 for reliable LASSO-based variable selection. The sample is therefore inadequate for the modelling approach employed. |
| D4.2 | Were continuous and categorical predictors handled appropriately? | PN | Age was categorised into three groups (<45, 45–60, >60 years). Indwelling catheter days was categorised into three groups (<14, 14–28, >28 days). The best parametric (lambda) choice in the LASSO model was made using a five-fold cross-validation of the minimum criteria. Categorisation of continuous variables (age, catheter duration) loses information and introduces threshold instability. No assessment of the linearity assumption on the log-odds scale for continuous predictors is reported. The cut-points used appear to be clinically motivated rather than data-driven, which is slightly preferable to purely data-driven thresholds, but no formal justification is provided. |
| D4.3 | Were all enrolled participants included in the analysis? | PY | Following the inclusion and exclusion criteria, 380 patients were finally included in the training set. The study describes a clear patient flow from the surgical department records, with explicit exclusion criteria stated. Unlike other included reports, there is no mention of additional exclusions for missing data beyond the stated eligibility criteria, suggesting relatively complete data capture. However, the exclusion of patients requiring "comprehensive medical records" (D1.2) implies some degree of case exclusion for data completeness without explicit quantification. |
| D4.4 | Were missing data handled appropriately? | U | All patients possess comprehensive medical records is stated as an inclusion criterion, which implies that patients with incomplete data were excluded at the eligibility stage rather than handled analytically. The number of patients excluded for incomplete records and the nature of missing variables are not reported. It is therefore unclear whether the completeness requirement introduced selection bias. No imputation or sensitivity analysis is reported. However, unlike Li Mengchao 2025 and Liu Huiying 2025, the missing data issue is embedded in the eligibility criteria rather than explicitly noted as a post-enrolment exclusion, making the extent of the problem difficult to assess. This is rated U (No Information) rather than N, as the scale of the problem cannot be determined. |
| D4.5 | Was selection of predictors based on univariable analysis avoided? | Y | The best predictive risk factor for patients with ICa-RCCS was selected by LASSO regression. Firstly, the significant features with non-zero coefficients were selected. Then, multivariate logistic regression analyses were performed to establish a risk model by integrating these selected features. The use of LASSO for variable selection — rather than univariable screening followed by multivariable analysis — is a methodologically superior approach that avoids the well-documented problems of two-stage univariable-then-multivariable selection. This is a notable methodological strength relative to Liu Huiying 2025 and Li Mengchao 2025, both of which used univariable pre-screening. LASSO applies regularisation simultaneously across all candidate variables, reducing but not eliminating optimism. |
| D4.6 | Were complexities in the data accounted for appropriately? | U | No information is provided on whether clustering effects (e.g., operating surgeon, admission period, surgical approach — laparoscopic vs. abdominal) or temporal trends across the two-year study period were considered. The study spans January 2020 to December 2021, overlapping with the COVID-19 pandemic period, during which surgical volumes, infection control practices, and antibiotic stewardship may have changed. The two surgical approaches (laparoscopic 95.3% vs. abdominal 4.7%) are not addressed as a potential clustering factor. |
| D4.7 | Were relevant model performance measures evaluated appropriately? | PY | A calibration curve was drawn to assess the calibration of this risk model. The C-index was calculated to assess the model's discriminating efficiency. To compute the accurate C-index, the bootstrap repeated sampling method was used with 1000 random samples to obtain the value. DCA was used to ascertain the model's clinical utility by analyzing net benefit at different threshold probabilities. C-index (equivalent to AUC for binary outcomes), calibration curve, and DCA together constitute a reasonable performance evaluation framework. However, Hosmer-Lemeshow test, Brier score, calibration slope, and calibration intercept are not reported. The calibration curve is presented visually (Figure 3) but without quantitative calibration metrics. |
| D4.8 | Were model overfitting and optimism accounted for? | PN | The prediction nomogram's C-index was 0.810 (95% CI: 0.759–0.861), while the bootstrapping validation C-index was 0.7930, indicating strong discrimination. Bootstrap resampling (1000 samples) was used for internal validation, and the bootstrap-corrected C-index (0.793) is modest lower than the apparent C-index (0.810), suggesting some degree of optimism correction. This is a positive feature. However, it is unclear whether the bootstrap was used specifically to compute an optimism-corrected C-index (as per Harrell's method) or primarily for calibration curve construction. The reduction from 0.810 to 0.793 is modest, which is consistent with the relatively low EPV (4.6) producing moderate overfitting. No shrinkage correction of regression coefficients is reported. No external validation has been performed. |
| D4.9 | Do predictors and their assigned weights correspond to the multivariable analysis results? | PY | Table 2 presents the prediction factors for the UTI risk nomogram, reporting hazard ratios (labelled as HR but likely OR given logistic regression), 95% CIs, and P-values for all five final predictors. The nomogram (Figure 2) is constructed directly from the multivariable logistic regression results and is internally consistent with the reported coefficients. However, the regression output labels estimates as 'HR (95% CI)' rather than 'OR (95% CI)'. Given that the stated method is logistic regression, this is likely a reporting error; the coefficients and values reported are consistent with logistic regression output. Readers should interpret these as odds ratios. |
| D4.10 | Was model performance evaluated in different subgroups of participants? | N | No subgroup performance analyses are reported. Given that the model includes patients with both stage IB and stage IIb cervical cancer, and both laparoscopic and abdominal surgical approaches, the absence of subgroup analyses represents a missed opportunity to assess performance heterogeneity across clinically meaningful patient subgroups. |
| D4.11 | Was model performance evaluated using calibration? | PN | When the calibration curve of the nomogram was used to predict the risk of UTIs in patients with ICA-RCCS, it revealed a good level of agreement with the UTIs that were detected. A calibration curve is presented visually (Figure 3) showing reasonable agreement between predicted and observed probabilities. However, no Hosmer-Lemeshow test P-value, calibration slope, calibration intercept, or Brier score is reported. Visual inspection of the calibration curve alone is insufficient for formal calibration assessment, as the curve does not include confidence intervals and quantitative deviations from ideal calibration cannot be assessed from the figure alone. |
| D4.12 | Was model performance evaluated in an external validation dataset? | N | Firstly, this is a single-centre retrospective study with small sample size. Further external validation is required to be performed to determine whether targeted interventions based on this nomogram could reduce the risk of UTI. All reported performance is from the derivation dataset with bootstrap internal validation only. No external validation in an independent cohort has been performed, which the authors themselves acknowledge as a limitation. |

**Domain 4 overall RoB: H**

Rationale: Multiple analytical limitations: critically low candidate EPV (4.6); categorisation of continuous variables without linearity assessment; no formal quantitative calibration metrics; bootstrap internal validation only without external validation; and a reporting inconsistency (HR vs. OR labelling) that cannot be resolved from the published text. The use of LASSO for variable selection (D4.5 = Y) is a relative strength compared to other included reports, and the bootstrap-corrected C-index (D4.8) represents a partial mitigation of overfitting concerns, but these do not offset the other analytical deficiencies.

**Overall Judgment Summary**

| Domain | Risk of Bias | Applicability |
| --- | --- | --- |
| D1 Participants | H | H |
| D2 Predictors | H | L |
| D3 Outcome | H | L |
| D4 Analysis | H | — |
| Overall | H | H |

**PROBAST Assessment: Yuting Liu 2023**

**Report:** Liu Y, Li Y, Huang Y, et al. Prediction of catheter-associated urinary tract infections among neurosurgical intensive care patients: a decision tree analysis. *World Neurosurg.* 2023; 170: 123–132.

**Domain 1 — Participants**

|  | **Signalling Question** | **Response** | **Justification** |
| --- | --- | --- | --- |
| D1.1 | Were appropriate data sources used? | Y | Data were extracted from the hospital information system and patients' files, which were saved in the database maintained by the infection control office and the department of neurosurgery. Patient demographics, comorbidities, GCS, admission season, length of stay, assisted ventilation, surgical operation, and laboratory indicators were collected. An institutional electronic record system combined with infection control office data is an appropriate data source for a CAUTI prediction model in NICU inpatients. |
| D1.2 | Were all inclusions and exclusions of participants appropriate? | Y | The study participants were adult patients (age ≥18 years) who were admitted to the NICU, and patients with newly inserted urethral catheters that had been indwelling for more than 48 hours were enrolled. Non-catheterised patients and those with positive urine culture within less than 48 hours after admission to the NICU were excluded. Moreover, patients who stayed in the NICU for less than 48 hours were excluded. These criteria are clinically appropriate and ensure that only genuinely at-risk patients with confirmed negative baseline urine culture are included. |
| D1.3 | Were participants enrolled at a similar and relevant point in their disease or condition? | Y | Enrolment required a newly inserted urethral catheter indwelling for more than 48 hours with a negative baseline urine culture, ensuring that all participants enter the risk period at a comparable and clinically relevant point. No prevalent cohort bias is evident. |
| D1.4 | Was the sample size appropriate? | N | A total of 537 adult patients admitted to the NICU during the study period were finally included. Of the 537 study participants, 21 patients developed CAUTI. All 16 independent variables were included in both the CHAID and CRT decision models. With 21 CAUTI events and 16 candidate variables, EPV = 1.3 — critically inadequate by any accepted standard. This is the lowest EPV among all reports evaluated in this series. No formal sample size calculation is reported. |

**Domain 1 overall RoB: H**

Rationale: D1.4 is rated N due to a critically low EPV of 1.3 (21 events, 16 candidate variables). D1.1–D1.3 are satisfactory. The high-risk judgment is driven entirely by the severely inadequate sample size, not by the retrospective design.

**Domain 1 Applicability: H**

Compared with patients admitted to the ICU, the use of urinary catheters and the rate of CAUTIs are more frequently seen in patients admitted to the neurosurgical intensive care units (NICUs) because patients admitted to the NICU often suffer from a disturbance of consciousness and a brain injury. The study population is exclusively NICU patients at a single tertiary hospital in Yangzhou, Jiangsu. This highly specific neurosurgical critical care population may not generalise to general ICU, mixed medical-surgical, or non-ICU catheterised inpatients.

**Domain 2 — Predictors**

|  | **Signalling Question** | **Response** | **Justification** |
| --- | --- | --- | --- |
| D2.1 | Were predictors defined and assessed in a similar way for all participants? | PY | Patient demographics (age and sex), presentation date and time, comorbidities, Glasgow Coma Scale (GCS), admission month divided into 4 seasons according to temperature and relative humidity, length of stay in the NICU, assisted ventilation, surgical operation, and laboratory indicators, including albumin, neutrophil count, and lymphocyte count within 48 hours, were collected. Data were double-checked and imported into SPSS software for data analysis. Double data entry is a positive feature supporting measurement consistency. However, no formal data dictionary or inter-rater reliability assessment is reported. |
| D2.2 | Were predictor assessments made without knowledge of outcome data? | PY | Most predictors (age, sex, admission diagnosis, GCS, comorbidities, laboratory values within 48 hours, admission season) are fixed or early-measured characteristics recorded at or near admission, independent of subsequent CAUTI development. However, length of stay in the NICU is a time-varying variable that accumulates during the same admission in which CAUTI is ascertained, introducing potential concurrent measurement. |
| D2.3 | Are all predictors available at the time the model is intended to be used? | PN | The scoring model is largely based on clinical variables that can be obtained on admission, including age ≥60 years, female sex, GCS score ≤8, epilepsy diagnosis, admission to the NICU in the summer, and ALB level <35 g/L. Therefore, this established scoring model allows the early and easy identification of NICU patients at high risk of CAUTIs. The model is explicitly designed for use at admission. However, the 9 variables associated with CAUTI in the CRT model included time of indwelling catheter (P = 0.001), which is a time-varying variable that cannot be known at admission. Furthermore, length of stay in the NICU similarly accumulates over the course of hospitalisation. While the final scoring model (Clinical Implications section) focuses on admission-available variables, the decision tree itself incorporates catheter dwell time as a splitting variable, creating an inconsistency between the model development process and the stated clinical application. |

**Domain 2 overall RoB: H**

Rationale: D2.3 is rated PN. The CRT decision tree — the primary model reported — incorporates indwelling catheter time as one of nine splitting variables, yet the stated clinical application is admission-time risk identification. The final scoring model selectively uses only admission-available variables, but the derivation tree on which it is based includes a time-varying predictor, creating a fundamental inconsistency.

**Domain 2 Applicability: L**

The predictors used in the final scoring model (age, sex, GCS, epilepsy diagnosis, admission season, albumin) are standard clinical variables universally available at NICU admission. No proprietary instruments or specialised measurements are required.

**Domain 3 — Outcome**

|  | **Signalling Question** | **Response** | **Justification** |
| --- | --- | --- | --- |
| D3.1 | Was the outcome determined appropriately? | PN | CAUTI was diagnosed according to the National Health and Safety Net and National Centers for Disease Control in 2015, with the growth of a single uropathogen with >100,000 colony forming units (CFU)/ml representing CAUTI. This definition requires microbiological confirmation with a specific bacterial count threshold, which is appropriate. However, the definition used omits the clinical symptom/sign requirement. |
| D3.2 | Was a pre-specified or standard outcome definition used? | PN | The study cites NHSN/CDC 2015 as the source for the CAUTI definition but applies only part of that standard. According to the NHSN/CDC in 2005, the definition used for CAUTI needs to meet 3 conditions (fever; suprapubic tenderness, costovertebral angle pain or tenderness; positive urine culture and bacterial species ≥10⁵ CFU/ml), but this study defined CAUTI as meeting the conditions of fever and urine culture with more than 10⁵ CFU/mL of one bacterial species. This may lead to an overdiagnosis of CAUTI. The authors themselves acknowledge this as a limitation. The applied definition is therefore a non-standard partial adaptation of the NHSN/CDC criteria that may systematically overidentify CAUTI cases. |
| D3.3 | Were predictors excluded from the outcome definition? | Y | The CAUTI outcome is defined by microbiological culture and fever. None of the final scoring model predictors (age, sex, GCS, epilepsy, admission season, albumin) are components of the outcome definition. |
| D3.4 | Was the outcome assessed without knowledge of predictor values? | U | No information is provided on whether clinicians or infection control staff who ascertained CAUTI status were blinded to predictor values. In a retrospective study, formal blinding is not performed and cannot be confirmed. |
| D3.5 | Was the time interval between predictor assessment and outcome determination appropriate? | PN | As noted in D2.3, indwelling catheter time is incorporated in the CRT tree as a splitting variable and is measured concurrently with the outcome ascertainment period. The temporal separation between predictor measurement and outcome determination is not cleanly established for this variable. |
| D3.6 | Was there a sufficient proportion of participants with the outcome? | N | Of the 537 study participants, 21 patients developed CAUTI. The CAUTI rate was 4.44‰ (21/4730), with a total of 4730 indwelling urinary days. The absolute number of 21 CAUTI events is critically small — the smallest absolute event count among all reports evaluated in this series. The study has limitations such as its single-centre nature and relatively small sample size, in particular the inclusion of only 5 patients as the "high risk" group in our scoring model. The authors themselves acknowledge this. With only 5 patients in the high-risk group, risk stratification estimates are highly unstable. |

**Domain 3 overall RoB: H**

Rationale: D3.2 is rated PN due to the use of a non-standard partial CAUTI definition that omits symptom criteria and may overdiagnose CAUTI, acknowledged by the authors themselves. D3.5 is rated PN due to concurrent measurement issues. D3.6 is rated N due to the critically small absolute event count of 21, with only 5 patients in the high-risk stratum.

**Domain 3 Applicability: H**

The non-standard CAUTI definition (fever plus urine culture ≥10⁵ CFU/mL, without the full three-condition NHSN/CDC requirement) may identify a different case mix from studies using the complete standard definition. The very low CAUTI rate (4.44 per 1000 catheter days) may reflect both the quality improvement programme in this specific NICU and the definitional differences, limiting comparability with other included reports.

**Domain 4 — Analysis**

|  | **Signalling Question** | **Response** | **Justification** |
| --- | --- | --- | --- |
| D4.1 | Were there an appropriate number of participants with the outcome? | N | EPV = 1.3 (see D1.4); critically inadequate for any modelling approach. Even against the final 6-variable scoring model, EPV = 3.5. Both figures are critically inadequate by any accepted standard, and substantially below all other reports in this series. The decision tree algorithm's data-driven recursive partitioning with EPV = 1.3 almost certainly produces severely overfit trees that capitalise on random variation. |
| D4.2 | Were continuous and categorical predictors handled appropriately? | PN | Age was dichotomised at 60 years. GCS was dichotomised at 8. ALB was dichotomised at 35 g/L. Length of indwelling catheter was trichotomised (<7, 7–14, >14 days). The parameters of the final tree model were set as follows: 1) maximum tree depth of 5, 2) minimum number of cases in the parent node of 10 and 3) in the child node of 5 and significance level of 0.05. The dichotomisation of continuous variables loses information and introduces threshold instability. The cut-points appear clinically motivated, but no formal justification or linearity assessment is provided. With only 21 events, tree depth of 5 and minimum child node size of 5 are permissive parameters that will produce highly overfit trees. |
| D4.3 | Were all enrolled participants included in the analysis? | PY | From the flow chart, 662 patients were initially screened, 118 were excluded for eligibility reasons (age <18 years n=38, NICU time <48h n=68, no indwelling catheters n=12), and 7 were excluded with missing data, leaving 537 included. The flow diagram is explicit and transparent. Seven patients were excluded for missing data, a small and clearly reported number. |
| D4.4 | Were missing data handled appropriately? | PY | Seven patients with missing data were excluded and explicitly reported in the flow diagram. This is a small number (1.3% of the screened post-eligibility cohort) and is unlikely to introduce substantial selection bias. This is the most transparent missing data reporting among all included reports evaluated to date. |
| D4.5 | Was selection of predictors based on univariable analysis avoided? | Y | The construction of the decision tree model was carried out in 2 phases. In the first phase, potential variables related to the dependent variable were selected in terms of the clinical and professional reasons. In the second phase, both outstanding variables in the tree and significant variables in the table of importance to the model of independent variables were chosen to attempt to construct the most concise and accurate tree. Finally, through different methods and parameter adjustments, various variable combinations were tried. All 16 independent variables were included in both the CHAID and CRT decision models. Unlike Liu Huiying 2025, Li Mengchao 2025, and Liu 2023 (the previously assessed Chinese logistic regression reports), this study enters all 16 candidate variables directly into the decision tree models without prior univariable screening. This is a methodological strength relative to two-stage univariable-then-multivariable selection. |
| D4.6 | Were complexities in the data accounted for appropriately? | U | No information is provided on whether clustering effects (e.g., treating neurosurgeon, admission period, type of neurological condition) or temporal trends within the one-year study period were considered. The study population covers a diverse range of admission diagnoses (cerebral haemorrhage 50.1%, brain injury 25.7%, aneurysm 13.6%, epilepsy 8.0%, brain tumour 2.6%), and potential heterogeneity of CAUTI risk across diagnostic subgroups is not addressed. |
| D4.7 | Were relevant model performance measures evaluated appropriately? | PY | The performance of the decision tree model was evaluated by determining the sensitivity, specificity, overall accuracy, and area under the receiver operating characteristic curve. Brier score was defined as the mean squared difference between the observed and predicted outcomes (range, 0–1), with smaller values indicating better calibration of the model. The CRT model (AUC = 0.920, 95% CI: 0.786–0.918) performs better than the CHAID model (AUC = 0.852, 95% CI: 0.883–0.918). The Brier score was 0.026 in the CRT model and 0.032 in the CHAID model. The reporting of both AUC and Brier score is a relative strength. However, calibration curves, Hosmer-Lemeshow test, and decision curve analysis are absent. Reported CIs contain apparent typographical errors: the CRT CI lower bound (0.786) is below the point estimate (0.920), and the CHAID CI upper bound (0.918) exceeds the point estimate in one reported version. These values require verification against the source publication. |
| D4.8 | Were model overfitting and optimism accounted for? | N | No bootstrap resampling, cross-validation, or optimism correction is performed. All performance metrics (AUC = 0.920, Brier score = 0.026) are apparent derivation performance estimates with no correction for optimism. Patients were classified into 3 risk groups based on the cut-off value of the predicted probability. Among all the patients in this study, the incidence of CAUTIs was 1.1% (5/452) in low-risk patients, 15% (12/80) in medium-risk patients and 80% (4/5) in high-risk patients. With EPV = 1.3 and no overfitting correction, the reported AUC of 0.920 almost certainly reflects severe overfitting. The high-risk group contains only 5 patients, making the 80% estimate highly unstable. No external validation is reported. |
| D4.9 | Do predictors and their assigned weights correspond to the multivariable analysis results? | PY | The decision tree structures are presented as figures (Figure 2A and 2B) with node-level CAUTI proportions and P-values for each split. The final scoring model variables are explicitly stated in the Clinical Implications section. The tree structures are internally consistent with the reported node statistics. However, the relationship between tree node probabilities and the final scoring model construction is not formally described — it is unclear exactly how the six scoring model variables were derived from the tree output. |
| D4.10 | Was model performance evaluated in different subgroups of participants? | N | No subgroup performance analyses are reported. Given the diversity of admission diagnoses (cerebral haemorrhage, brain injury, aneurysm, epilepsy, brain tumour), the absence of subgroup analyses is a notable omission, particularly since epilepsy is identified as a key risk factor and constitutes only 8% of the study population. |
| D4.11 | Was model performance evaluated using calibration? | PN | The Brier score was 0.026 in the CRT model and 0.032 in the CHAID model, indicating good agreement between the predicted and observed rates in the 2 models. The Brier score is reported, which is a relative strength compared to most other included reports. However, no calibration curve, calibration slope, calibration intercept, or Hosmer-Lemeshow test is presented. The Brier score alone is insufficient for comprehensive calibration assessment, particularly given the very low event rate (3.9%) which can produce artificially low Brier scores even for poorly discriminating models. |
| D4.12 | Was model performance evaluated in an external validation dataset? | N | No external validation is performed. All performance metrics are derivation-set apparent performance estimates. The study has limitations such as its single-centre nature and relatively small sample size. Therefore, future multi-centre studies could be considered. The authors acknowledge the need for external validation. |

**Domain 4 overall RoB: H**

Rationale: Multiple critical analytical failures: EPV of 1.3 is the lowest in this series and critically inadequate; no overfitting correction of any kind (no bootstrap, no cross-validation); all performance metrics are apparent derivation estimates; the high-risk group contains only 5 patients; no calibration curve or DCA; and no external validation. The use of all 16 variables in the decision tree without univariable pre-screening (D4.5 = Y) and the reporting of Brier score (D4.11 = PN) are relative strengths but do not offset the other critical deficiencies.

**Overall Judgment Summary**

| Domain | Risk of Bias | Applicability |
| --- | --- | --- |
| D1 Participants | H | H |
| D2 Predictors | H | L |
| D3 Outcome | H | H |
| D4 Analysis | H | — |
| Overall | H | H |

**PROBAST Assessment: Li Yuping 2023**

**Report:** Li Y, Liu Y, Huang Y, et al. Development and validation of a user-friendly risk nomogram for the prediction of catheter-associated urinary tract infection in neuro-intensive care patients. *Intensive Crit Care Nurs.* 2023; 74: 103329.

**Domain 1 — Participants**

|  | **Signalling Question** | **Response** | **Justification** |
| --- | --- | --- | --- |
| D1.1 | Were appropriate data sources used? | Y | Data were extracted from the hospital health information system. Before deciding on the potential risk variables, the investigators carried out a comprehensive literature review and held a panel meeting with both doctors and nurses. Three independent researchers extracted, reviewed, and verified the data from the electronic medical records. An institutional electronic health information system is an appropriate data source for a CAUTI prediction model in NICU inpatients. |
| D1.2 | Were all inclusions and exclusions of participants appropriate? | PY | This retrospective cohort study was performed on neuro-ICU patients admitted to one tertiary hospital in Yangzhou city from January 2019 to January 2020. Patients aged above 18 years and who received an indwelling catheter when admitted to the neuro-ICU were included. Patients with UTIs before admission or with a length of neuro-ICU stay of less than 48 hours were excluded. These criteria are clinically appropriate. However, among the 719 patients admitted to the neuro-ICU, 47 patients had missing data and were excluded. The number excluded for missing data and whether missingness was related to CAUTI outcome is not reported, introducing uncertainty about potential selection bias. |
| D1.3 | Were participants enrolled at a similar and relevant point in their disease or condition? | Y | Inclusion required an indwelling catheter at NICU admission and exclusion of patients with pre-existing UTI, ensuring all participants entered the risk period at a comparable and clinically relevant starting point. No prevalent cohort bias is evident. |
| D1.4 | Was the sample size appropriate? | N | The overall incidence of CAUTIs was 3.91% (21/537), with 4.37% (17/389) in the training set and 2.70% (4/148) in the validation set. The nomogram was developed in the training set of 389 patients with 17 CAUTI events. A multivariate logistic regression analysis based on the results of the univariate analyses was performed. Variables included in the final multivariable models were selected according to their physiological relevance and statistical significance in univariate analysis using a P value threshold of 0.25. The univariate screening admitted 12 variables to multivariable consideration (Table 2 footnote identifies 12 variables passing P<0.25). With 17 events in the training set and 12 candidate variables entering logistic regression, EPV = 1.4 — critically inadequate by any accepted standard, and among the lowest in this series. No formal sample size calculation is reported. |

**Domain 1 overall RoB: H**

Rationale: D1.4 is rated N due to a critically low EPV of 1.4 (17 events in the training set, 12 candidate variables). This is the second-lowest EPV in this series, comparable to the twin publication Yuting Liu 2023 (EPV=1.3). D1.1–D1.3 are satisfactory.

**Domain 1 Applicability: H**

The neuro-ICU is a specialized ICU that typically provides personalized multidisciplinary clinical care for critically ill neurological and neurosurgical patients. Principle diagnoses leading to neuro-ICU admission include brain trauma, spinal cord injury, acute cerebrovascular disease, severe infection of the central nervous system, continued seizures, and post-operative care following neurosurgical procedures. The study population is exclusively NICU patients at a single tertiary hospital in Yangzhou, limiting generalisability to general ICU, mixed medical-surgical, or non-ICU catheterised patients.

**Domain 2 — Predictors**

|  | **Signalling Question** | **Response** | **Justification** |
| --- | --- | --- | --- |
| D2.1 | Were predictors defined and assessed in a similar way for all participants? | PY | The following data were collected: (1) patients' general information (age, sex, BMI, comorbidities, length of neuro-ICU stay); (2) information on admission, including diagnosis, admission season, and Glasgow coma scale (GCS); (3) laboratory examination (levels of albumin, white blood cell, neutrophil count, and lymphocyte count within 48 hours); and (4) whether the patient received assisted ventilation or surgery. Three independent researchers extracted and verified data. This supports measurement consistency. However, no formal data dictionary or inter-rater reliability assessment is reported. |
| D2.2 | Were predictor assessments made without knowledge of outcome data? | PY | Most predictors (age, sex, BMI, comorbidities, admission diagnosis, GCS, admission season, laboratory values within 48 hours) are fixed or early-measured characteristics recorded at or near admission, independent of subsequent CAUTI development. However, length of neuro-ICU stay is a time-varying variable that accumulates during the same admission in which CAUTI is ascertained. |
| D2.3 | Are all predictors available at the time the model is intended to be used? | N | Three of the four factors incorporated in the prediction model can be assessed on admission, and these included age, admission diagnosis, and serum albumin levels. Moreover, the model does not require any clinician input or nonroutine biomarker data and thus makes it easier for both doctors and nurses to apply during clinical practice. However, the fourth predictor — length of neuro-ICU stay greater than 30 days (OR: 272.2; 95% CI: 8.3–8963.5) — is a time-varying variable that is fundamentally unknowable at the time of catheter insertion or NICU admission. The nomogram mainly included clinical risk factors at admission and length of neuro-ICU stay. Length of neuro-ICU stay >30 days is the single most influential predictor in the nomogram, carrying the highest regression coefficient (β=5.067) and the largest OR (272.2) among all four final predictors. This variable cannot be known at the time the model is intended to be used for early risk identification, constituting temporal leakage of the same fundamental nature as identified in Liu Huiying 2025, Li Mengchao 2025, Wang Fang 2023, and Yuting Liu 2023. |

**Domain 2 overall RoB: H**

Rationale: D2.3 is rated N. Length of neuro-ICU stay >30 days is the dominant predictor in the nomogram (OR=272.2, the largest effect estimate in the model) but is intrinsically unknowable at NICU admission.

**Domain 2 Applicability: L**

The nomogram contains a small number of true risk factors and showed a good overall predictive value. Three of the four factors incorporated in the prediction model can be assessed on admission, including age, admission diagnosis, and serum albumin levels. Moreover, the model does not require any clinician input or nonroutine biomarker data. The admission-available predictors are standard, universally available clinical variables.

**Domain 3 — Outcome**

|  | **Signalling Question** | **Response** | **Justification** |
| --- | --- | --- | --- |
| D3.1 | Was the outcome determined appropriately? | PN | The NHSN defined CAUTI as a urine culture of ≥10⁵ CFU/mL plus one or more of the clinical criteria, including temperature >38°C, frequency, urgency, dysuria, or suprapubic tenderness. However, this definition was difficult to apply here because neuro-ICU patients usually had severe neurological symptoms or were unconscious. Therefore, in this study, CAUTIs were defined based on a urine culture with more than 10⁵ CFU/mL of one bacterial species, which is consistent with several previous studies. The applied definition omits the clinical symptom/sign requirement of the full NHSN standard. |
| D3.2 | Was a pre-specified or standard outcome definition used? | PN | The study cites the NHSN definition but explicitly applies only the microbiological component, omitting the symptom criteria. The justification provided — that neuro-ICU patients often cannot report symptoms — is clinically reasonable, but results in a non-standard partial adaptation that may overidentify CAUTI cases. This is the same definitional issue identified in the twin publication Yuting Liu 2023, which the authors of that report themselves acknowledged as a limitation causing potential overdiagnosis. |
| D3.3 | Were predictors excluded from the outcome definition? | Y | The CAUTI outcome is defined by urine culture ≥10⁵ CFU/mL. None of the four final nomogram predictors (age, admission diagnosis, length of neuro-ICU stay, albumin levels) are components of the outcome definition. |
| D3.4 | Was the outcome assessed without knowledge of predictor values? | U | Urine tests and urine cultures were routinely collected when patients were first admitted to the neuro-ICU. After admission, routine urine cultures were regularly carried out every three days during their stay in the neuro-ICU. No information is provided on whether clinicians or infection control staff who ascertained CAUTI status were blinded to predictor values. In a retrospective study, formal blinding is not performed and cannot be confirmed. |
| D3.5 | Was the time interval between predictor assessment and outcome determination appropriate? | N | Length of neuro-ICU stay >30 days is both a predictor and a measure of time elapsed during the outcome ascertainment period. A patient staying >30 days in the NICU has by definition been at risk of CAUTI for the entire duration, meaning that the predictor and the outcome accumulate over the same time window. This is the most direct form of temporal confounding identified in this series —the predictor is defined by the same time interval as the outcome ascertainment period — a patient staying >30 days has been at risk throughout, making causal direction indeterminate. |
| D3.6 | Was there a sufficient proportion of participants with the outcome? | N | The overall incidence of CAUTIs was 3.91% (21/537), with 4.37% (17/389) in the training set and 2.70% (4/148) in the validation set. The absolute number of 17 CAUTI events in the training set is critically small — the second-lowest absolute event count in this series — and with only 4 events in the validation set, performance assessment in the validation set is entirely unstable. |

**Domain 3 overall RoB: H**

Rationale: D3.2 is rated PN due to use of a non-standard partial CAUTI definition. D3.5 is rated N — the most severe temporal confounding in this series, as the dominant predictor (length of neuro-ICU stay >30 days) is constitutively defined by the same time window over which CAUTI is ascertained. D3.6 is rated N due to 17 events in the training set and only 4 events in the validation set.

**Domain 3 Applicability: H**

The non-standard CAUTI definition (urine culture ≥10⁵ CFU/mL without symptom requirement) is the same adaptation used in the twin publication Yuting Liu 2023 and differs from the complete NHSN/CDC, IDSA 2009, and ESCMID definitions used in other included reports. The very low CAUTI rate (3.91%) may reflect both the quality of the NICU infection control programme and the modified definition, limiting comparability.

**Domain 4 — Analysis**

|  | **Signalling Question** | **Response** | **Justification** |
| --- | --- | --- | --- |
| D4.1 | Were there an appropriate number of participants with the outcome? | N | With 17 CAUTI events in the training set and 12 candidate variables entering logistic regression, EPV = 1.4. Even relative to the four final predictors (EPV = 17/4 = 4.25), the sample is inadequate. The candidate EPV of 1.4 is the most relevant metric and is critically below any accepted threshold. |
| D4.2 | Were continuous and categorical predictors handled appropriately? | PN | Age was dichotomised at 60 years. The nomogram model included age (<60 years, ≥60 years), length of neuro-ICU (<7 days, 7–14 days, 15–30 days or >30 days), admission diagnosis (cerebral haemorrhage, aneurysm, brain tumour, epilepsy or brain injury), and albumin levels (<35 g/L, ≥35 g/L). Dichotomisation of continuous variables (age, albumin) loses information and introduces threshold instability. Length of neuro-ICU stay is categorised into four groups, which introduces multiple thresholds without formal justification of cut-points. No assessment of linearity on the log-odds scale for continuous predictors is reported. |
| D4.3 | Were all enrolled participants included in the analysis? | PY | Among the 719 patients admitted to the neuro-ICU from January 2019 to January 2020, 70 stayed in the neuro-ICU for less than 48 hours, 47 did not receive indwelling catheters, 47 patients had missing data, and 18 patients were aged below 18 years. The remaining 537 patients fulfilled the inclusion criteria and were further analysed. The flow diagram is explicit and transparent, with the number excluded for missing data clearly stated as 47. |
| D4.4 | Were missing data handled appropriately? | N | As noted in D4.3, 47 patients (6.5% of the 719 screened) were excluded for missing data. Unlike the twin publication Yuting Liu 2023 (which excluded only 7 patients for missing data), this report excludes 47 — a substantially larger proportion. No description of which variables were missing, whether missingness was related to CAUTI outcome, or any sensitivity analysis is provided. Complete-case exclusion of 47 patients (including potential CAUTI cases) without justification of the missing data mechanism introduces meaningful risk of selection bias. |
| D4.5 | Was selection of predictors based on univariable analysis avoided? | N | A multivariate logistic regression analysis based on the results of the univariate analyses was performed to calculate the odds ratios and 95% confidence intervals of the independent variables. Variables included in the final multivariable models were selected according to their physiological relevance and statistical significance in univariate analysis using a P value threshold of 0.25. This two-stage univariable-then-multivariable selection approach is a well-recognised source of optimism and selection bias, identical to the approach used in Liu Huiying 2025, Li Mengchao 2025, and the twin publication Yuting Liu 2023. |
| D4.6 | Were complexities in the data accounted for appropriately? | U | No information is provided on whether clustering effects (e.g., treating neurosurgeon, admission period, type of neurological condition) or temporal trends within the one-year study period were considered. The dataset is shared with Yuting Liu 2023, which covers the same patient population with the same diversity of admission diagnoses. |
| D4.7 | Were relevant model performance measures evaluated appropriately? | PY | The predictive model was evaluated using three metrics, including discriminative capacity, calibration ability, and clinical effectiveness. The AUC was used to evaluate the discriminative ability of the nomogram. Calibration accuracy was evaluated by a calibration plot. The clinical effectiveness was evaluated by decision curve analysis and clinical impact curve analysis. The AUC achieved using the nomogram model was 0.855 (95% CI: 0.8–1.0) in the training set and 0.888 (95% CI: 0.8–1.0) in the validation set. AUC, calibration curve, DCA, and clinical impact curve together constitute a reasonable performance evaluation framework. However, Hosmer-Lemeshow test, Brier score, calibration slope, and calibration intercept are not reported. The confidence intervals for AUC are extremely wide (0.8–1.0 in both sets), reflecting the critically small event counts. |
| D4.8 | Were model overfitting and optimism accounted for? | N | No bootstrap resampling, cross-validation, or optimism correction of any kind is reported for the discrimination estimate. The training set AUC of 0.855 is an apparent derivation performance estimate with no correction for optimism. The mean absolute error was 0.013, revealing good prediction consistency and calibration — but this calibration metric is derived from the validation set calibration curve without bootstrap correction. With EPV=1.4 and two-stage variable selection, the reported AUC of 0.855 almost certainly reflects severe overfitting. |
| D4.9 | Do predictors and their assigned weights correspond to the multivariable analysis results? | PY | Table 2 presents the multivariate analysis results including regression coefficients (β), standard errors (SE), Wald statistics, ORs with 95% CIs, and P-values for all four final predictors. The nomogram is constructed from these results and is internally consistent with the reported coefficients. |
| D4.10 | Was model performance evaluated in different subgroups of participants? | N | No subgroup performance analyses are reported. Given the diverse admission diagnoses (cerebral haemorrhage 50.1%, brain injury 25.7%, aneurysm 13.6%, epilepsy 8.0%, brain tumour 2.6%) and the fact that epilepsy is identified as a key risk factor, the absence of subgroup analyses is a notable omission. |
| D4.11 | Was model performance evaluated using calibration? | PN | In the validation process, the calibration curve of the nomogram was drawn in the validation set. The mean absolute error was 0.013, revealing good prediction consistency and calibration. A calibration curve is presented (Figure S1) with mean absolute error reported. However, no Hosmer-Lemeshow test P-value, calibration slope, calibration intercept, or Brier score is provided. With only 4 CAUTI events in the validation set, the calibration curve is based on extremely sparse data and the mean absolute error estimate is highly unstable. |
| D4.12 | Was model performance evaluated in an external validation dataset? | N | The 537 patients were split into a training set (n=389) and validation set (n=148) from the same institution and time period. The model was developed in a large teaching hospital within a single city; thus, the predictive model requires further external validation and multicentre studies to confirm its clinical applicability. This is internal temporal/random split validation only. No external validation in an independent cohort has been performed. |

**Domain 4 overall RoB: H**

Rationale: Multiple critical analytical failures: EPV of 1.4 is critically inadequate; two-stage univariable pre-screening; no overfitting correction for discrimination estimates; 47 patients excluded for missing data without justification; no external validation; and validation set performance is based on only 4 CAUTI events, rendering all validation-set metrics highly unstable. The reporting of DCA and clinical impact curve (D4.7) is a relative strength, but does not offset the fundamental sample size and analytical deficiencies.

**Overall Judgment Summary**

| Domain | Risk of Bias | Applicability |
| --- | --- | --- |
| D1 Participants | H | H |
| D2 Predictors | H | L |
| D3 Outcome | H | H |
| D4 Analysis | H | — |
| Overall | H | H |

**PROBAST Assessment: Xia Liyao 2022**

**Report:** Xia L, Wang C, Liu S. Development and validation of a risk prediction model for postoperative catheter-associated urinary tract infection in patients with gynecologic malignancies. *Chin J Mod Nurs.* 2022; 28(6): 809–813.

**Domain 1 — Participants**

|  | **Signalling Question** | **Response** | **Justification** |
| --- | --- | --- | --- |
| D1.1 | Were appropriate data sources used? | Y | Convenience sampling was adopted to select postoperative 800 patients with gynecologic malignancies and indwelling urinary catheters in Zhejiang Cancer Hospital as the research object. A single tertiary oncology hospital's inpatient records constitute an appropriate data source for this specific population. |
| D1.2 | Were all inclusions and exclusions of participants appropriate? | PY | Inclusion criteria were: (1) meeting the diagnostic criteria for gynecologic malignancies; (2) age ≥18 years; (3) postoperative transurethral indwelling catheter; (4) no mental illness, with signed informed consent. Exclusion criteria were: (1) preoperative indwelling catheter; (2) urinary tract infection already present before catheterisation; (3) combined with serious physical diseases of the heart, lung, brain, or kidney. Post-enrolment exclusion criteria (termed “elimination criteria” by the authors): recurrence during treatment; requirement for second surgery. These criteria are clinically appropriate and restrict the population to catheterised postoperative patients at genuine risk. However, the "elimination criteria" (recurrence during treatment, need for second surgery) are post-enrolment exclusions that may introduce selection bias if eliminated patients differ systematically from retained patients in their CAUTI risk profile. The number eliminated on these criteria is not reported. |
| D1.3 | Were participants enrolled at a similar and relevant point in their disease or condition? | Y | All patients are enrolled at the time of postoperative transurethral catheter insertion following gynecologic malignancy surgery. Exclusion of patients with pre-existing UTI ensures a comparable starting point for the risk period. No prevalent cohort bias is evident. |
| D1.4 | Was the sample size appropriate? | PN | In the derivation group, 124 cases developed CAUTI, with a CAUTI incidence of 20.7% (124/600). In the validation group, 39 cases developed CAUTI, with a CAUTI incidence of 19.5% (39/200). Univariate analysis identified tumour type, age, operation time, intraoperative urinary tract injury, postoperative bladder irrigation, and indwelling catheter duration as influential factors (P<0.05), and these six variables were entered into binomial logistic regression analysis. With 124 CAUTI events in the derivation set and 6 candidate variables entered into logistic regression, EPV = 20.7 — the highest among all single-centre Chinese reports evaluated in this series, and marginally meeting the conventional EPV ≥ 20 threshold. However, the full survey instrument included more candidate variables than the 6 entering the multivariable step; the effective candidate EPV is lower than 20.7, though the exact value cannot be determined from the published text. |

**Domain 1 overall RoB: H**

Rationale: Rationale: D1.2 is rated PY due to post-enrolment elimination criteria applied to an unquantified number of patients, which may have introduced selection bias if eliminated patients differed systematically from retained patients in CAUTI risk. D1.4 is rated PN: although EPV = 20.7 relative to the six variables entering the multivariable step appears adequate, this figure is inflated by the two-stage pre-screening that restricted the effective candidate pool to statistically significant variables only; the true candidate EPV against the full survey instrument variable pool is substantially lower. No formal sample size calculation was reported. Overall, the combination of unquantified post-enrolment exclusions and inflated apparent EPV from two-stage variable selection places Domain 1 at high risk of bias.

**Domain 1 Applicability: H**

Only cases from one tertiary hospital were collected, so whether the results are applicable to other institutions remains to be further verified. The study population is exclusively female patients with gynecologic malignancies (cervical cancer, endometrial cancer, ovarian cancer, vaginal cancer) at a single specialist oncology centre in Hangzhou. The model's applicability is restricted to postoperative gynecologic oncology patients and may not generalise to other catheterised surgical populations, non-oncological patients, or multi-centre settings.

**Domain 2 — Predictors**

|  | **Signalling Question** | **Response** | **Justification** |
| --- | --- | --- | --- |
| D2.1 | Were predictors defined and assessed in a similar way for all participants? | PY | Through reviewing domestic and foreign literature and holding research group discussions, CAUTI risk factors were selected and a survey questionnaire was developed. The questionnaire included: (1) patient basic information: age, marital status, reproductive history, BMI, etc.; (2) disease-related factors: tumour type, diabetes, hypertension, etc.; (3) treatment-related factors: preoperative chemotherapy, surgical approach, operation time, intraoperative urinary tract injury, indwelling catheter duration, etc. A standardised survey instrument with literature-derived items is a positive feature. However, no formal inter-rater reliability assessment or data dictionary is reported, and the survey was completed retrospectively by researchers from medical records. |
| D2.2 | Were predictor assessments made without knowledge of outcome data? | PY | For patients whose urinary catheters were removed during hospitalisation, researchers completed the CAUTI risk factor survey form after catheter removal. For patients who were discharged with urinary catheters, the same researcher conducted telephone follow-up every two weeks after discharge, inquiring about and recording urinary tract infection symptoms and catheter status. The retrospective completion of the survey form after catheter removal means that the researcher knew whether CAUTI had occurred at the time of recording some predictor values, introducing potential for outcome-informed predictor recording. This is a meaningful concern for subjectively assessed variables, though most predictors (age, tumour type, surgical details) are objective and unlikely to be systematically distorted. |
| D2.3 | Are all predictors available at the time the model is intended to be used? | N | Binomial Logistic regression analysis showed that age ≥60 years old, intraoperative urinary tract injury, postoperative bladder irrigation, and indwelling catheter for more than 7 days were risk factors for CAUTI. The model is presented as a tool for early identification of high-risk patients. However, indwelling catheter duration >7 days is a time-varying variable that cannot be known at the time of catheter insertion. Furthermore, postoperative bladder irrigation — while knowable in principle as a planned post-operative intervention — may itself be a consequence of intraoperative urinary tract injury and evolving post-operative management rather than a fixed baseline predictor. These two of the four final predictors introduce temporal leakage of the same fundamental nature identified across multiple other reports in this series. |

**Domain 2 overall RoB: H**

Rationale: D2.3 is rated N. Indwelling catheter duration >7 days — the predictor with the largest regression coefficient (β=1.57, OR=4.78) and highest assigned score in the risk scoring model — is fundamentally unknowable at the time of catheter insertion.

**Domain 2 Applicability: L**

Age ≥60 years old, intraoperative urinary tract injury, postoperative bladder irrigation, and indwelling catheter for more than 7 days are standard clinical variables routinely recorded in gynecologic oncology settings. No proprietary instruments or specialised measurements are required.

**Domain 3 — Outcome**

|  | **Signalling Question** | **Response** | **Justification** |
| --- | --- | --- | --- |
| D3.1 | Was the outcome determined appropriately? | PY | The CAUTI diagnostic criteria were judged according to the "Guidelines for Prevention and Control of Catheter-associated Urinary Tract Infections (Trial)" issued by the former Ministry of Health of China in 2010. Patients had urinary irritation symptoms such as frequent urination, urgency, and painful urination, or lower abdominal tenderness, renal percussion pain, with or without fever, and met one of the following conditions: (1) bacterial culture of clean midstream urine indicated Gram-positive bacteria or fungi >10⁴ CFU/mL; (2) bacterial culture of clean midstream urine indicated Gram-negative bacteria >10⁵ CFU/mL. This definition requires both clinical symptoms/signs AND microbiological confirmation, with organism-specific thresholds. This is the most detailed and internally differentiated CAUTI outcome definition among all single-centre Chinese reports evaluated in this series. |
| D3.2 | Was a pre-specified or standard outcome definition used? | PY | The applied definition is based on the 2010 Chinese Ministry of Health guideline, which is a nationally recognised standard. The differentiated bacterial count thresholds (Gram-positive/fungi >10⁴ CFU/mL; Gram-negative >10⁵ CFU/mL) reflect established microbiological principles and are consistent with international standards. This definition is more complete than that used in Yuting Liu 2023 and Li Yuping 2023 (which omitted symptom criteria) and comparable to Wang Fang 2023 (which used the IDSA 2009 definition). However, the follow-up approach introduces some heterogeneity: in-hospital CAUTI is ascertained by clinical teams, while post-discharge CAUTI is ascertained by telephone follow-up, raising questions about whether the diagnostic threshold was uniformly applied. |
| D3.3 | Were predictors excluded from the outcome definition? | Y | The CAUTI outcome is defined by clinical symptoms/signs and microbiological culture. None of the four final model predictors (age, intraoperative urinary tract injury, postoperative bladder irrigation, catheter duration >7 days) are components of the outcome definition. |
| D3.4 | Was the outcome assessed without knowledge of predictor values? | U | For patients whose catheters were removed during hospitalisation, researchers completed the survey form after catheter removal. For patients discharged with catheters, the same researcher conducted telephone follow-up every two weeks. The same researcher who collected predictor data also ascertained outcomes via telephone follow-up, without any blinding procedure. This introduces potential for outcome ascertainment influenced by knowledge of predictor values, though the impact is limited by the objective nature of the diagnostic criteria. |
| D3.5 | Was the time interval between predictor assessment and outcome determination appropriate? | PN | As noted in D2.3, indwelling catheter duration >7 days is measured over the same period during which CAUTI is being ascertained. The temporal overlap between the key predictor and the outcome ascertainment window is the same concurrent measurement problem identified across multiple other reports in this series. |
| D3.6 | Was there a sufficient proportion of participants with the outcome? | Y | In the derivation group, 124 cases developed CAUTI, with a CAUTI incidence of 20.7% (124/600). In the validation group, 39 cases developed CAUTI, with a CAUTI incidence of 19.5% (39/200). With 124 events in the derivation set and 39 events in the validation set, absolute event counts are the largest among all single-centre Chinese reports in this series. The 19.5–20.7% event rate is consistent with the published literature range for this specific population. |

**Domain 3 overall RoB: H**

Rationale: D3.2 is rated PY — the outcome definition is the most complete among all single-centre Chinese reports in this series, which is a notable relative strength. However, D3.4 cannot be confirmed (same researcher collected predictors and ascertained outcomes without blinding), D3.5 is rated PN due to concurrent measurement, and the telephone follow-up approach for post-discharge CAUTI ascertainment introduces diagnostic uncertainty. The overall domain is rated High primarily due to the temporal leakage implications of D3.5 and the unblinded retrospective data collection.

**Domain 3 Applicability: L**

The outcome definition — based on the 2010 Chinese Ministry of Health guideline with organism-specific bacterial count thresholds and symptom requirement — is broadly comparable to internationally recognised CAUTI standards and is the most complete national-standard definition among all included Chinese reports. Applicability concern is low for the outcome domain.

**Domain 4 — Analysis**

|  | **Signalling Question** | **Response** | **Justification** |
| --- | --- | --- | --- |
| D4.1 | Were there an appropriate number of participants with the outcome? | PY | With 124 CAUTI events in the derivation set and 6 variables entering logistic regression, EPV = 20.7 — marginally meeting the conventional threshold of EPV ≥ 20. This is the most adequate EPV among all single-centre Chinese reports in this series. However, the two-stage pre-screening approach (univariable then multivariable) means the effective candidate pool is larger than 6, and EPV calculated against the full candidate variable pool is lower. |
| D4.2 | Were continuous and categorical predictors handled appropriately? | PN | Age was dichotomised at 60 years. Indwelling catheter duration was dichotomised at 7 days. According to the multivariate regression model, independent risk factors were obtained. With the minimum B value set to 1, each risk factor was assigned a value based on the ratio of its B value to the minimum B value, rounded to the nearest integer. Age ≥60 years: 1 point; intraoperative urinary tract injury: 2 points; postoperative bladder irrigation: 2 points; indwelling catheter duration >7 days: 2 points. Dichotomisation of continuous variables loses information and introduces threshold instability. The scoring system assignment (rounding B value ratios to integers) further compounds information loss. No assessment of linearity on the log-odds scale is reported. |
| D4.3 | Were all enrolled participants included in the analysis? | PY | The study divided 800 patients into derivation samples (600 cases) and validation samples (200 cases) by random number table method. Demographic characteristics and disease conditions of derivation and validation groups were not statistically significantly different (P>0.05), indicating comparability. The flow of participants is described and the derivation/validation split is explicit. However, the number of patients eliminated under the post-enrolment elimination criteria (recurrence, second surgery) is not reported, as noted in D1.2. |
| D4.4 | Were missing data handled appropriately? | U | No information is provided on missing predictor data within the 800 included patients. The survey instrument was completed retrospectively from medical records, and no description of which variables had missing values or how missing values were handled is provided. Complete data availability is implied by the full-sample analysis but not explicitly confirmed. |
| D4.5 | Was selection of predictors based on univariable analysis avoided? | N | Variables with statistically significant differences in univariate analysis were entered into binomial Logistic regression analysis to construct the risk prediction model. This two-stage univariable-then-multivariable selection approach is a well-recognised source of optimism and selection bias, identical to the approach used in Liu Huiying 2025, Li Mengchao 2025, and Li Yuping 2023. Only variables reaching statistical significance (P<0.05) in univariable analysis were eligible for multivariable consideration, which is a more restrictive threshold than the P<0.25 used in Li Yuping 2023 and further increases the risk of excluding true predictors from the model. |
| D4.6 | Were complexities in the data accounted for appropriately? | U | No information is provided on whether clustering effects (e.g., operating surgeon, surgical approach — laparoscopic vs. open), temporal trends within the six-month study period, or potential heterogeneity across tumour types (cervical, endometrial, ovarian, vaginal cancer) were considered. The study covers multiple gynecologic cancer types with different surgical extents and baseline UTI risks, and potential performance heterogeneity across subgroups is not addressed. |
| D4.7 | Were relevant model performance measures evaluated appropriately? | PN | In the validation group of 200 cases, the receiver operating characteristic curve was used to evaluate the prediction performance of the model. The area under the receiver operating characteristic curve of the validation group was 0.79 with a statistical difference [95%CI (0.72, 0.87), P<0.01], and the sensitivity, specificity and Youden index were 71.8%, 78.3% and 50.1% respectively. AUC with 95% CI is reported for the validation set. However, no calibration curve, Hosmer-Lemeshow test, Brier score, calibration slope, calibration intercept, or decision curve analysis is reported. Performance evaluation is restricted to discrimination and threshold-based metrics only, without any calibration or clinical utility assessment. This is the most limited performance evaluation framework among all included reports evaluated in this series. |
| D4.8 | Were model overfitting and optimism accounted for? | PN | The patients were divided into derivation samples and validation samples according to the ratio of 3:1 by random number table method. In the derivation group of 600 cases, the predictive factors were analyzed by binomial Logistic regression analysis and the risk prediction model was developed. In the validation group of 200 cases, the receiver operating characteristic curve was used to evaluate the prediction performance of the model. Random split-sample internal validation is performed, which provides a basic level of overfitting assessment. The derivation group AUC was 0.78, sensitivity 71%, specificity 75.6%, Youden index 46.6%. The validation group AUC was 0.79. The marginal increase in AUC from derivation (0.78) to validation set (0.79) is unusual — validation set performance typically decreases relative to derivation — and may reflect random variation from the 3:1 split rather than genuine stability. No bootstrap correction, cross-validation, or shrinkage correction of regression coefficients is reported. No external validation is performed. |
| D4.9 | Do predictors and their assigned weights correspond to the multivariable analysis results? | PY | Table 2 presents the binomial logistic regression analysis results including regression coefficients, standard errors, Wald χ² values, P-values, and OR values for all four final predictors. The scoring system assigns values based on the ratio of each factor's B value to the minimum B value, rounded to the nearest integer. The scoring system is internally consistent with the reported regression coefficients. |
| D4.10 | Was model performance evaluated in different subgroups of participants? | N | No subgroup performance analyses are reported. Given the inclusion of four tumour types (cervical cancer 46.5%, ovarian cancer 35%, endometrial cancer 17.2%, vaginal cancer 1.3%) with different surgical extents and CAUTI risk profiles, the absence of subgroup analyses is a notable omission. |
| D4.11 | Was model performance evaluated using calibration? | N | No calibration assessment of any kind is reported — no calibration curve, no Hosmer-Lemeshow test, no Brier score, no calibration slope or intercept. This is the only report among all evaluated studies that reports no calibration metric whatsoever, including not even a visual calibration curve. Performance evaluation is entirely restricted to AUC and threshold-based metrics. |
| D4.12 | Was model performance evaluated in an external validation dataset? | N | Only cases from one tertiary hospital were collected. This study only performed internal validation of the model; external validation awaits subsequent research. All reported performance is from an internal random split-sample validation. No external validation has been performed. |

**Domain 4 overall RoB: H**

Rationale: Multiple analytical limitations: two-stage univariable pre-screening with P<0.05 threshold (D4.5 = N); complete absence of calibration assessment of any kind (D4.11 = N); no bootstrap correction or shrinkage; internal random split validation only without external validation (D4.12 = N); and dichotomisation of continuous variables without linearity assessment (D4.2 = PN). The EPV of 20.7 relative to the multivariable-entered variables (D4.1 = PY) and the explicit split-sample internal validation (D4.8 = PN) are relative strengths compared to other single-centre Chinese reports in this series, but do not offset the other analytical deficiencies, particularly the complete absence of calibration.

**Overall Judgment Summary**

| Domain | Risk of Bias | Applicability |
| --- | --- | --- |
| D1 Participants | H | H |
| D2 Predictors | H | L |
| D3 Outcome | H | L |
| D4 Analysis | H | — |
| Overall | H | H |

**PROBAST Assessment: Lyu Yangyang 2022**

**Report:** Lyu Y, Huang H, Liang C, Jin Q. Risk factors for emergency room patients with catheter-associated urinary tract infections and the establishment of prediction model. *J Wenzhou Med Univ.* 2022; 52(2): 126–131, 138.

**Domain 1 — Participants**

|  | **Signalling Question** | **Response** | **Justification** |
| --- | --- | --- | --- |
| D1.1 | Were appropriate data sources used? | Y | The retrospective method was used to review case data, and the clinical data of patients during their stay in the emergency department were collected, including age, sex, HbA1c, serum albumin, BMI, catheter dwell time, number of catheter replacements, whether prophylactic antibiotics were used, and whether bladder irrigation was performed. An institutional medical record system at a large tertiary public hospital is an appropriate data source. |
| D1.2 | Were all inclusions and exclusions of participants appropriate? | PY | Patients with malignant tumors, blood diseases, autoimmune diseases, pre-existing infection before the procedure, combined mental/intellectual disabilities, and incomplete case data were excluded. These exclusion criteria are broadly appropriate. However, no explicit inclusion criteria (beyond receipt of an indwelling catheter in the emergency room) are described, and the number excluded under each criterion is not reported. The exclusion of patients with incomplete case data without reporting the number so excluded introduces uncertainty about potential selection bias. |
| D1.3 | Were participants enrolled at a similar and relevant point in their disease or condition? | PY | A routine urinalysis was performed at the time of catheter insertion, and this study used a negative urinalysis result as one of the baseline and inclusion criteria. The use of a negative baseline urinalysis as an inclusion criterion ensures that participants enter the risk period at a comparable starting point. However, the study is described as a cross-sectional study in the Limitations section, which is inconsistent with a cohort design; this definitional ambiguity introduces uncertainty about the uniformity of the enrolment point. |
| D1.4 | Was the sample size appropriate? | PY | The incidence of CAUTI was 7.98% during the period of this study. Among 1,780 patients with indwelling catheters, 142 were confirmed to have developed urinary tract infection, an infection rate of 7.98%. Seven variables — age, sex, HbA1c, BMI, catheter dwell time, number of catheter replacements, and serum albumin — were entered as independent variables into binary logistic regression analysis (univariable screening followed by backward likelihood method). With 142 CAUTI events and 7 candidate variables entering the multivariable step, EPV = 20.3 — the highest among all single-centre Chinese reports evaluated in this series, and meeting the conventional EPV ≥ 20 threshold. However, as with Xia Liyao 2022, the two-stage pre-screening approach means that the full candidate variable pool is larger than 7, making the effective candidate EPV lower. No formal sample size calculation is reported. |

**Domain 1 overall RoB: H**

Rationale: D1.4 is rated PY — the EPV of 20.3 relative to the multivariable-entered variables is the strongest in this series. D1.2 is rated PY due to unquantified missing-data exclusions. D1.3 is rated PY due to the cross-sectional/cohort definitional inconsistency noted in the Limitations. Overall, Domain 1 is the most methodologically adequate among all single-centre Chinese reports in this series for the participant domain, though it falls short of a Low RoB rating due to the incomplete reporting of exclusion numbers.

**Domain 1 Applicability: H**

This unit, as one of the largest public tertiary hospitals in southern Zhejiang, has a patient population with good representativeness in the emergency room. However, the study population is exclusively emergency room patients at a single tertiary hospital in Wenzhou. The emergency setting is the most unusual clinical context among all included reports — distinct from NICU, gynecologic oncology, and general surgical populations — and the model's applicability is restricted to emergency department catheterised patients, limiting generalisability to other inpatient settings.

**Domain 2 — Predictors**

|  | **Signalling Question** | **Response** | **Justification** |
| --- | --- | --- | --- |
| D2.1 | Were predictors defined and assessed in a similar way for all participants? | PY | The above demographic data were collected during the patient's emergency admission period, and the laboratory indicators were all from the first examination results upon emergency admission. A routine urinalysis was performed at the time of catheter insertion. The consistent use of admission-time laboratory values (first examination results) is a positive feature supporting measurement uniformity. However, no formal data dictionary, inter-rater reliability assessment, or explicit description of who collected the data is provided. |
| D2.2 | Were predictor assessments made without knowledge of outcome data? | PY | Most predictors (age, sex, HbA1c, BMI, albumin) are fixed or admission-measured characteristics recorded independently of subsequent CAUTI development. HbA1c and albumin are explicitly stated to be from the first admission examination, which reduces the risk of outcome-informed measurement for these variables. However, catheter dwell time and number of catheter replacements are time-varying variables that accumulate during the same admission in which CAUTI is ascertained. |
| D2.3 | Are all predictors available at the time the model is intended to be used? | PN | Age ≥60 years, BMI ≥30 kg/m², indwelling time of catheter ≥3 days, and HbA1c were independent risk factors for CAUTI in emergency room patients, while serum albumin level was a protective factor for CAUTI. Three of the five final model predictors (age, HbA1c, BMI) are fixed characteristics available at catheter insertion. However, catheter dwell time ≥3 days is a time-varying variable that cannot be known at the time of catheter insertion. The American Epidemiological Society points out that catheter dwell time is the most important risk factor for the occurrence of infection. While this variable is clinically important, it is temporally concurrent with the outcome ascertainment period, constituting the same temporal leakage problem identified across all other single-centre Chinese reports in this series. |

**Domain 2 overall RoB: H**

Rationale: D2.3 is rated PN. Catheter dwell time ≥3 days carries OR=3.135 (β=1.143) — the largest regression coefficient among all five final predictors — and is fundamentally unknowable at the time of catheter insertion. This constitutes the same systematic temporal leakage problem identified in all other single-centre Chinese CAUTI prediction reports in this series.

**Domain 2 Applicability: L**

The final model predictors (age, HbA1c, BMI, catheter dwell time, serum albumin) are standard clinical variables universally available in emergency settings. The HbA1c and serum albumin selected are all routine admission laboratory indicators in clinical practice, so the combination of these two indicators can make more accurate and scientific clinical decision-making for outcome prediction without adding additional test items or increasing patient burden. The use of HbA1c rather than a diabetes diagnosis as a predictor is a methodologically thoughtful choice, and the admission-available predictors are clinically practical.

**Domain 3 — Outcome**

|  | **Signalling Question** | **Response** | **Justification** |
| --- | --- | --- | --- |
| D3.1 | Was the outcome determined appropriately? | PY | The CAUTI diagnostic criteria referred to the "Guidelines for Prevention and Control of Catheter-associated Urinary Tract Infections (Trial)" issued by the former Ministry of Health of China in 2010, combined with pathogen culture results and clinical symptoms. The 2010 Chinese Ministry of Health guideline requires both clinical symptoms and microbiological confirmation, which is appropriate. However, the specific symptom criteria and bacterial count thresholds applied are not enumerated in the text, making full verification of adherence to the standard impossible. |
| D3.2 | Was a pre-specified or standard outcome definition used? | PY | The 2010 Chinese Ministry of Health guideline is the same nationally recognised standard used in Xia Liyao 2022. However, unlike Xia Liyao 2022 — which explicitly enumerated the symptom criteria (frequency, urgency, dysuria, lower abdominal tenderness, renal percussion pain) and organism-specific bacterial count thresholds (Gram-positive/fungi >10⁴ CFU/mL; Gram-negative >10⁵ CFU/mL) — this report states only that the guideline was referenced "combined with pathogen culture results and clinical symptoms" without specifying which symptom criteria or bacterial count thresholds were applied. The definition is therefore less completely reported than Xia Liyao 2022, though the underlying standard is the same. |
| D3.3 | Were predictors excluded from the outcome definition? | Y | The CAUTI outcome is defined by clinical symptoms and pathogen culture results. None of the five final model predictors (age, HbA1c, BMI, catheter dwell time, albumin) are components of the outcome definition. |
| D3.4 | Was the outcome assessed without knowledge of predictor values? | U | For all included patients, if symptoms of urinary tract infection appeared, a sample was taken for urine culture to further confirm the diagnosis. No information is provided on whether clinicians who ascertained CAUTI status were blinded to predictor values. In a retrospective study, formal blinding is not performed and cannot be confirmed. |
| D3.5 | Was the time interval between predictor assessment and outcome determination appropriate? | PN | As noted in D2.3, catheter dwell time ≥3 days is measured over the same period during which CAUTI is being ascertained. The temporal overlap between the key predictor and the outcome ascertainment window is the same concurrent measurement problem identified across all other reports in this series. |
| D3.6 | Was there a sufficient proportion of participants with the outcome? | Y | Among 1,780 patients with indwelling catheters, 142 were confirmed to have developed urinary tract infection, an infection rate of 7.98%. With 142 CAUTI events in the total dataset, the absolute event count is the second largest among all single-centre Chinese reports in this series (after Xia Liyao 2022 with 124 derivation-set events), and is adequate for logistic regression modelling. |

**Domain 3 overall RoB: H**

Rationale: D3.2 is rated PY — the outcome definition references an appropriate national standard but does not enumerate the specific symptom and microbiological criteria applied. D3.5 is rated PN due to concurrent measurement of the key predictor and outcome. D3.4 cannot be confirmed. Despite the adequate event count (D3.6 = Y), the overall domain is rated High due to the temporal leakage and incomplete outcome definition reporting.

**Domain 3 Applicability: L**

The outcome definition references the 2010 Chinese Ministry of Health guideline, the same standard used in Xia Liyao 2022. The emergency room setting means that some CAUTI cases may have been missed after patient discharge, as the high rate of loss to follow-up in the emergency room makes CAUTI prevention and control more challenging. This introduces a potential underascertainment bias for post-discharge CAUTI, but is an inherent feature of the emergency setting rather than a modifiable methodological deficiency.

**Domain 4 — Analysis**

|  | **Signalling Question** | **Response** | **Justification** |
| --- | --- | --- | --- |
| D4.1 | Were there an appropriate number of participants with the outcome? | PY | With 142 CAUTI events and 7 candidate variables entering logistic regression, EPV = 20.3. This marginally meets the conventional EPV ≥ 20 threshold and is the highest EPV among all single-centre Chinese reports in this series. As noted, the full candidate pool assessed in univariable analysis is larger than 7, making the effective candidate EPV lower. |
| D4.2 | Were continuous and categorical predictors handled appropriately? | PN | Age was dichotomised at 60 years, BMI at 30 kg/m², and catheter dwell time at 3 days. HbA1c and serum albumin were treated as continuous variables in the regression model. The treatment of HbA1c and albumin as continuous variables is methodologically superior to dichotomisation and preserves information — this is a relative strength compared to Xia Liyao 2022 and other included reports that categorised all variables. However, no assessment of linearity on the log-odds scale for these continuous predictors is reported. In this study, a ROC analysis was performed on the continuous BMI raw data in advance, and the optimal cut-off value obtained with the maximum Youden index was 30.18 kg/m², so the cut-off value for BMI dichotomisation was set at 30 kg/m². The data-driven derivation of the BMI cut-point using an ROC-based Youden index is a positive feature for that variable, though this approach still introduces optimistic bias by selecting the cut-point that maximises discrimination in the derivation set. |
| D4.3 | Were all enrolled participants included in the analysis? | PY | Patients with incomplete case data were excluded. The total sample of 1,780 patients is stated as the analysis population, and all 142 CAUTI cases and 1,638 non-infected patients appear in Table 1, confirming complete-case analysis of the included cohort. However, the number of patients excluded for incomplete data is not reported, as noted in D1.2. |
| D4.4 | Were missing data handled appropriately? | U | Patients with incomplete case data were excluded as an eligibility criterion. The number excluded and the nature of missing variables are not reported. As with other included reports, the scale of the missing data problem cannot be determined. This is rated U (No Information) rather than N, as the impact cannot be assessed from the published text. |
| D4.5 | Was selection of predictors based on univariable analysis avoided? | N | According to professional judgment, possible risk factors were first subjected to univariable analysis using χ² test or t-test to screen for meaningful variables, and then binary logistic regression analysis was performed to find out the independent risk factors for CAUTI. This two-stage univariable-then-multivariable selection approach is a well-recognised source of optimism and selection bias, identical to the approach used in Liu Huiying 2025, Li Mengchao 2025, Li Yuping 2023, and Xia Liyao 2022. |
| D4.6 | Were complexities in the data accounted for appropriately? | U | No information is provided on clustering effects (e.g., treating physician, triage category, admission diagnosis) or temporal trends within the 30-month study period (June 2018 to December 2020), which overlaps with the COVID-19 pandemic period (from January 2020 onwards). Pandemic-related changes in emergency volume, patient mix, infection control practices, and catheter management protocols could introduce temporal heterogeneity that is not addressed. |
| D4.7 | Were relevant model performance measures evaluated appropriately? | PN | The fitting degree of the model was acceptable by Hosmer-Lemeshow test (χ²=13.383, P=0.099). ROC analysis showed that the AUC of the regression model was 0.870, with the corresponding sensitivity being 83.10% and specificity 78.45%. The reporting of both AUC and Hosmer-Lemeshow test is a relative strength — this is one of only two reports in this series (alongside Liu 2023 from the same Wang Fang series) to report the Hosmer-Lemeshow test. However, no calibration curve, Brier score, calibration slope, calibration intercept, or decision curve analysis is reported. The Hosmer-Lemeshow test P=0.099 marginally exceeds the 0.05 threshold (acceptable calibration), but the test is known to have low power with small event counts and high power to detect trivial miscalibration with large samples; its interpretation requires caution. |
| D4.8 | Were model overfitting and optimism accounted for? | N | No bootstrap resampling, cross-validation, internal or external validation, or optimism correction of any kind is reported. The current domestic related research usually lists the odds ratios of influencing factors, and rarely quantifies the prediction of risk factors for outcomes, which to a certain extent affects the clinical practical value. All performance metrics (AUC=0.870, sensitivity=83.10%, specificity=78.45%) are apparent derivation performance estimates with no correction for optimism. Despite the relatively adequate EPV (20.3), the absence of any validation procedure means the reported performance may be optimistically biased. |
| D4.9 | Do predictors and their assigned weights correspond to the multivariable analysis results? | Y | Table 2 presents the logistic regression analysis for CAUTI in emergency room patients, reporting β coefficients, standard errors, Wald statistics, P-values, ORs, and 95% CIs for all five final predictors. The risk prediction model for CAUTI in emergency room catheterised patients is: Logit(P) = −4.571 + 0.493×age + 0.446×HbA1c + 1.116×BMI + 1.143×catheter dwell time − 0.110×albumin. The explicit regression equation is internally consistent with the reported coefficients in Table 2. The reporting of the full regression equation is a notable strength, as it allows model recalibration and independent application — a feature absent from most other included reports. |
| D4.10 | Was model performance evaluated in different subgroups of participants? | N | No subgroup performance analyses are reported. Given the emergency room setting encompasses a highly heterogeneous patient population (diverse admission diagnoses, age range 25–91 years), the absence of subgroup analyses is a notable omission. |
| D4.11 | Was model performance evaluated using calibration? | PN | The fitting degree was acceptable by Hosmer-Lemeshow test (χ²=13.383, P=0.099). The Hosmer-Lemeshow test is reported, which is a relative strength. However, no calibration curve, Brier score, calibration slope, or calibration intercept is presented. The Hosmer-Lemeshow P=0.099 indicates marginal acceptable calibration, and the absence of a calibration curve prevents visual assessment of calibration across the probability range. |
| D4.12 | Was model performance evaluated in an external validation dataset? | N | This study is a cross-sectional study, making it difficult to evaluate the causal relationship between variables and outcomes. At the same time, it is retrospective data, and some key parameters cannot be further analyzed. No external validation in an independent cohort is performed. All reported performance is from the derivation dataset only, with no correction for optimism. |

**Domain 4 overall RoB: H**

Rationale: Two-stage univariable pre-screening (D4.5 = N); no overfitting correction or validation of any kind (D4.8 = N, D4.12 = N); no calibration curve or DCA (D4.11 = PN); and no subgroup analyses (D4.10 = N). The relative strengths — EPV of 20.3 (D4.1 = PY), treatment of HbA1c and albumin as continuous variables (D4.2 = PN), reporting of the Hosmer-Lemeshow test (D4.7 = PN), and publication of the full regression equation (D4.9 = Y) — are the most collectively favourable analytical features among all single-centre Chinese reports in this series, but do not offset the fundamental absence of any validation procedure.

**Overall Judgment Summary**

| Domain | Risk of Bias | Applicability |
| --- | --- | --- |
| D1 Participants | H | H |
| D2 Predictors | H | L |
| D3 Outcome | H | L |
| D4 Analysis | H | — |
| Overall | H | H |

**PROBAST Assessment: Liao Liping 2022**

**Report:** Liao LP, Chen P, Wang K, et al. Construction and validation of a nomogram model for risk prediction of catheter-associated urinary tract infection in traumatic brain injury patients with indwelling catheter. *Chin J Nosocomiol.* 2022; 32(12): 1798–1802.

**Domain 1 — Participants**

|  | **Signalling Question** | **Response** | **Justification** |
| --- | --- | --- | --- |
| D1.1 | Were appropriate data sources used? | Y | Retrospective clinical and epidemiological data were collected from patients with traumatic brain injury and indwelling urinary catheter treated in the neurosurgery department of Chongqing Emergency Medical Center. An institutional medical record system at a large tertiary emergency medical centre is an appropriate data source for this population. |
| D1.2 | Were all inclusions and exclusions of participants appropriate? | PY | Inclusion criteria: (1) age 18–85 years; (2) meeting the diagnostic criteria of the American TBI Management Guidelines (2017) confirmed by CT or MRI; (3) negative urine culture on admission; (4) indwelling catheter ≥48 hours. Exclusion criteria: (1) severe damage to vital organs (heart, lung, kidney); (2) abnormal coagulation function; (3) severe autoimmune or endocrine diseases; (4) urinary tract infection on admission; (5) incomplete clinical data. These criteria are clinically appropriate. However, the number excluded under each criterion — particularly those with incomplete clinical data — is not reported, introducing uncertainty about potential selection bias. |
| D1.3 | Were participants enrolled at a similar and relevant point in their disease or condition? | Y | Inclusion required a negative urine culture on admission and an indwelling catheter for ≥48 hours, ensuring all participants entered the risk period at a comparable baseline. No prevalent cohort bias is evident. |
| D1.4 | Was the sample size appropriate? | PN | A total of 815 patients were included. The derivation group comprised 570 patients, of whom 80 developed CAUTI (infection rate 14.04%); the validation group comprised 245 patients, of whom 41 developed CAUTI (infection rate 16.73%). Variables with statistical significance in univariate analysis were entered into multivariate logistic regression analysis. The independent risk factors identified were age ≥60 years, GCS score ≤8, indwelling catheter ≥30 days, ICU stay ≥14 days, and stress hyperglycaemia. With 80 CAUTI events in the derivation set and 5 final predictors, EPV = 16.0 — below the conventional threshold of EPV ≥ 20. However, univariate analysis identified 7 statistically significant variables (age, GCS, catheter duration, ICU stay, comorbidity history, stress hyperglycaemia, antibiotic duration ≥20 days) that were entered into multivariable consideration, giving an effective candidate EPV = 80/7 = 11.4. No formal sample size calculation is reported. |

**Domain 1 overall RoB: H**

Rationale: D1.4 is rated PN — the derivation-set EPV of 11.4 against the full candidate pool is below the conventional threshold, though better than most other single-centre Chinese reports in this series. D1.2 is rated PY due to unquantified missing-data exclusions. The overall sample size (815 patients, 121 total CAUTI events) is among the larger single-centre Chinese reports evaluated, but the EPV remains inadequate.

**Domain 1 Applicability: H**

This study is a retrospective case-control study with a single data source; multicentre, large-sample studies and validation are still needed to further revise and improve the model so that it is truly applicable to the screening of high-risk populations for CAUTI in clinical traumatic brain injury patients with indwelling catheters. The study population is exclusively traumatic brain injury patients at a single tertiary emergency medical centre in Chongqing, limiting generalisability to general neurosurgical, non-TBI, or non-emergency populations.

**Domain 2 — Predictors**

|  | **Signalling Question** | **Response** | **Justification** |
| --- | --- | --- | --- |
| D2.1 | Were predictors defined and assessed in a similar way for all participants? | PY | Retrospective data were collected on age, sex, Glasgow Coma Scale (GCS), indwelling catheter duration, catheter size, ICU length of stay, past medical history, stress hyperglycaemia, whether craniotomy was performed, and antibiotic use duration. Data were collected retrospectively from medical records. No formal data dictionary, inter-rater reliability assessment, or explicit description of data collection procedures is provided. |
| D2.2 | Were predictor assessments made without knowledge of outcome data? | PY | Most predictors (age, sex, GCS, comorbidity history, stress hyperglycaemia, craniotomy) are fixed or early-measured characteristics recorded at or near admission. However, indwelling catheter duration ≥30 days, ICU stay ≥14 days, and antibiotic use duration ≥20 days are all time-varying variables that accumulate during the same admission in which CAUTI is ascertained, raising the possibility that predictor values were recorded with knowledge of the outcome in a retrospective setting. |
| D2.3 | Are all predictors available at the time the model is intended to be used? | N | Age ≥60 years, stress hyperglycaemia, GCS ≤8, ICU stay ≥14 days, and indwelling catheter ≥30 days were independent risk factors for CAUTI in traumatic brain injury patients. Three of the five final predictors (age, stress hyperglycaemia, GCS) are available at or near admission. However, indwelling catheter duration ≥30 days and ICU stay ≥14 days are both time-varying variables that are fundamentally unknowable at the time of catheter insertion or ICU admission. The risk of CAUTI in patients with indwelling catheter duration ≥30 days was 6.1 times that of patients with catheter duration <30 days. The multivariate logistic regression analysis showed that ICU stay ≥14 days had OR = 5.255 (95% CI: 2.178–12.679). These two predictors — both carrying large effect estimates in the nomogram — constitute temporal leakage of the same fundamental nature identified across all other single-centre Chinese CAUTI prediction reports in this series. |

**Domain 2 overall RoB: H**

Rationale: D2.3 is rated N. Two of the five final nomogram predictors (indwelling catheter ≥30 days, OR=6.170; ICU stay ≥14 days, OR=5.255) are time-varying variables unknowable at catheter insertion. This is the same systematic temporal leakage problem identified in all other single-centre Chinese CAUTI prediction reports in this series, and in this report affects 2 of 5 final predictors simultaneously — a broader temporal contamination than in most other included reports.

**Domain 2 Applicability: L**

Three of the five final predictors (age ≥60 years, stress hyperglycaemia, GCS ≤8) are standard clinical variables available at or near NICU/neurosurgery admission. No proprietary instruments or specialised measurements are required.

**Domain 3 — Outcome**

|  | **Signalling Question** | **Response** | **Justification** |
| --- | --- | --- | --- |
| D3.1 | Was the outcome determined appropriately? | PY | CAUTI diagnosis was made according to the "Guidelines for Prevention and Control of Catheter-associated Urinary Tract Infections (Trial)" issued by the Ministry of Health of China in 2010. The 2010 Chinese Ministry of Health guideline requires both clinical symptoms and microbiological confirmation, which is an appropriate standard. However, the specific symptom criteria and bacterial count thresholds applied are not enumerated in the text, preventing full verification of adherence. |
| D3.2 | Was a pre-specified or standard outcome definition used? | PY | The 2010 Chinese Ministry of Health guideline is the same nationally recognised standard used in Xia Liyao 2022 and Lyu Yangyang 2022. As with Lyu Yangyang 2022, however, this report states only that the guideline was applied without specifying which symptom criteria or bacterial count thresholds were used. The definition is less completely reported than Xia Liyao 2022, which explicitly enumerated the full criteria. |
| D3.3 | Were predictors excluded from the outcome definition? | Y | The CAUTI outcome is defined by clinical symptoms and microbiological criteria. None of the five final nomogram predictors (age, stress hyperglycaemia, GCS, indwelling catheter duration, ICU stay) are components of the outcome definition. |
| D3.4 | Was the outcome assessed without knowledge of predictor values? | U | No information is provided on whether clinicians or infection control staff who ascertained CAUTI status were blinded to predictor values. In a retrospective study, formal blinding cannot be confirmed. |
| D3.5 | Was the time interval between predictor assessment and outcome determination appropriate? | N | Both indwelling catheter duration ≥30 days and ICU stay ≥14 days are measured over the same period during which CAUTI is being ascertained. Long-term indwelling urinary catheterisation disrupts the normal physiological environment of the urinary tract, reducing the physiological flushing of pathogens by urine, causing pathogens to ascend and leading to CAUTI. A patient with catheter duration ≥30 days has been continuously exposed to CAUTI risk throughout this period; the predictor value thus accumulates over the same window as outcome ascertainment. The concurrent measurement of two predictor variables with the outcome ascertainment window — affecting 2 of 5 final predictors — constitutes the broadest temporal confounding among all single-centre Chinese reports evaluated in this series. |
| D3.6 | Was there a sufficient proportion of participants with the outcome? | PY | A total of 815 patients with traumatic brain injury and indwelling urinary catheter were included, with 121 cases of CAUTI, giving an incidence rate of 14.85%. With 80 CAUTI events in the derivation set and 41 in the validation set, the absolute event counts are among the larger single-centre Chinese reports in this series, though EPV remains below optimal as noted in D1.4. |

**Domain 3 overall RoB: H**

Rationale: D3.5 is rated N — two of the five final predictors (catheter duration ≥30 days and ICU stay ≥14 days) are both measured concurrently with the outcome ascertainment period. This dual temporal confounding affecting 40% of the final model's predictors is the most extensive in this series. D3.2 is rated PY due to incomplete reporting of the applied CAUTI criteria. D3.4 cannot be confirmed.

**Domain 3 Applicability: L**

The outcome definition references the 2010 Chinese Ministry of Health guideline, the same standard used in Xia Liyao 2022 and Lyu Yangyang 2022. The CAUTI incidence rate in this study was 14.85%, which is higher than reported rates of 2.69% in ICU patients and 5.95% in critically ill patients from other studies, possibly related to the presence of consciousness disorders and long-term catheterisation in TBI patients. The higher observed event rate is clinically plausible for this specific TBI population and does not indicate a definitional problem.

**Domain 4 — Analysis**

|  | **Signalling Question** | **Response** | **Justification** |
| --- | --- | --- | --- |
| D4.1 | Were there an appropriate number of participants with the outcome? | PN | With 80 CAUTI events in the derivation set and 7 candidate variables from univariable screening, EPV = 11.4. This is below the conventional threshold of EPV ≥ 20. Against the 5 final predictors only, EPV = 16.0, still below the threshold. EPV is inadequate relative to any relevant candidate pool, though it is among the higher values in this series. |
| D4.2 | Were continuous and categorical predictors handled appropriately? | PN | Age was dichotomised at 60 years; GCS score at 8; indwelling catheter duration at 30 days; ICU stay at 14 days; antibiotic use duration at 20 days. All continuous predictors are dichotomised, losing information and introducing threshold instability. No assessment of linearity on the log-odds scale is reported, and no justification for the chosen cut-points beyond clinical convention is provided. |
| D4.3 | Were all enrolled participants included in the analysis? | PY | The derivation group comprised 570 patients and the validation group 245 patients, totalling 815. The flow of participants is described and the 70:30 derivation/validation split is explicit. However, the number excluded for incomplete clinical data under the eligibility criteria is not reported. |
| D4.4 | Were missing data handled appropriately? | U | Patients with incomplete case data were excluded as an eligibility criterion. The number excluded and the nature of missing variables are not reported. As with other included reports, the scale of the missing data problem cannot be determined from the published text. |
| D4.5 | Was selection of predictors based on univariable analysis avoided? | N | Variables with statistical significance in univariate analysis were entered into multivariate logistic regression analysis. This two-stage univariable-then-multivariable selection approach is a well-recognised source of optimism and selection bias, identical to the approach used in Liu Huiying 2025, Li Mengchao 2025, Li Yuping 2023, Xia Liyao 2022, and Lyu Yangyang 2022. |
| D4.6 | Were complexities in the data accounted for appropriately? | U | No information is provided on clustering effects (e.g., treating neurosurgeon, injury mechanism, injury severity score) or temporal trends within the three-year study period (January 2019 to December 2021), which overlaps with the COVID-19 pandemic period. Pandemic-related changes in patient mix, infection control practices, and catheter management protocols could introduce temporal heterogeneity that is not addressed. |
| D4.7 | Were relevant model performance measures evaluated appropriately? | PY | The ROC curve and calibration curve were used for internal and external validation in both groups to evaluate the discrimination and calibration of the prediction model. The AUC of the derivation group was 0.944 (95% CI: 0.922–0.965), and the validation group AUC was 0.930 (95% CI: 0.898–0.963). The Hosmer-Lemeshow goodness-of-fit test showed P=0.708 for the derivation group and P=0.831 for the validation group. AUC with 95% CI and Hosmer-Lemeshow test are reported for both derivation and validation groups — a relative strength compared to several other included reports. A calibration curve is also mentioned. However, Brier score, calibration slope, calibration intercept, and decision curve analysis are not reported. |
| D4.8 | Were model overfitting and optimism accounted for? | PN | Seventy percent of subjects were randomly selected as the derivation group and 30% as the verification group. Random split-sample internal validation is performed. The model was verified in the validation group. The AUC decreases marginally from 0.944 (derivation) to 0.930 (validation), which is a plausible and modest reduction consistent with internal validation. However, no bootstrap resampling, cross-validation, or shrinkage correction of regression coefficients is reported. The split-sample AUC attenuation (0.944→0.930) is modest and consistent with limited overfitting, but split-sample validation alone is insufficient to quantify or correct optimism; bootstrap or cross-validation with shrinkage correction would be required for a formal assessment. No external validation is performed. |
| D4.9 | Do predictors and their assigned weights correspond to the multivariable analysis results? | Y | Table 2 presents the multivariate logistic regression analysis results including β coefficients, standard errors, Wald statistics, ORs, and 95% CIs for all five final predictors. The nomogram (Figure 1) is constructed from these results and is internally consistent with the reported coefficients. The predictor with the largest β (age ≥60: β=3.481, OR=32.507) and the smallest β (GCS ≤8: β=0.896, OR=2.450) are clearly distinguished in the nomogram scale. |
| D4.10 | Was model performance evaluated in different subgroups of participants? | N | No subgroup performance analyses are reported. Given the broad range of TBI severity (GCS ranging from mild to severe) and the diversity of comorbidities and injury mechanisms, the absence of subgroup analyses is a notable omission. |
| D4.11 | Was model performance evaluated using calibration? | PN | The calibration curve showed good consistency between predicted and actual CAUTI risk in the derivation group (Hosmer-Lemeshow P=0.708), and the validation group calibration showed P=0.831. The Hosmer-Lemeshow test is reported for both derivation and validation groups — one of only two reports in this series to report this test (alongside Lyu Yangyang 2022). A calibration curve is presented. However, no Brier score, calibration slope, calibration intercept, or mean absolute error is provided. |
| D4.12 | Was model performance evaluated in an external validation dataset? | N | This study is a retrospective case-control study with a single data source; multicentre, large-sample studies and validation are still needed. All reported performance — including the “internal validation” in the validation group — is from an internal random split-sample of the same single-centre dataset. No external validation in an independent cohort has been performed. |

**Domain 4 overall RoB: H**

Rationale: Two-stage univariable pre-screening (D4.5 = N); no bootstrap or shrinkage correction (D4.8 = PN); no external validation (D4.12 = N); no DCA, Brier score, or calibration slope (D4.7 = PY only); EPV of 11.4 below threshold (D4.1 = PN); dichotomisation of all continuous predictors without linearity assessment (D4.2 = PN). The reporting of AUC with 95% CI and Hosmer-Lemeshow test in both derivation and validation groups (D4.7 = PY, D4.11 = PN) and the explicit split-sample validation with plausible AUC attenuation (D4.8 = PN) are relative strengths, but do not offset the fundamental analytical deficiencies.

**Overall Judgment Summary**

| Domain | Risk of Bias | Applicability |
| --- | --- | --- |
| D1 Participants | H | H |
| D2 Predictors | H | L |
| D3 Outcome | H | L |
| D4 Analysis | H | — |
| Overall | H | H |

**PROBAST Assessment: Li Zhe 2021**

**Report**: Li Z. Establishment of early warning model of catheter-related urinary tract infection in neurosurgical patients [D]. Shanxi University of Chinese Medicine, 2021.

**Domain 1 — Participants**

|  | **Signalling Question** | **Response** | **Justification** |
| --- | --- | --- | --- |
| D1.1 | Were appropriate data sources used? | Y | Retrospective clinical data were collected from neurosurgical patients with indwelling urinary catheters in a tertiary hospital in Shanxi Province, sourced from the institutional medical record system. Institutional medical records from a tertiary neurosurgical centre are an appropriate source for a CAUTI prediction model in this population. |
| D1.2 | Were all inclusions and exclusions of participants appropriate? | PY | Inclusion criteria: neurosurgical patients with indwelling catheter, age ≥18 years, complete clinical data. Exclusion criteria: pre-admission urinary tract infection or other infections, pregnancy or lactation. The criteria are clinically reasonable. However, the number of patients excluded due to incomplete data is not reported, and the study adopted a 1:3 random sampling instead of continuous enrollment of all eligible cases, introducing uncertainty of potential selection bias. |
| D1.3 | Were participants enrolled at a similar and relevant point in their disease or condition? | Y | Patients with pre-admission urinary tract infection were excluded. All participants entered the risk observation period from the start of catheterization with consistent baseline infection status, and no prevalent cohort bias was observed. |
| D1.4 | Was the sample size appropriate? | PN | A total of 300 patients were included, with 74 CAUTI events. For the 8 final independent predictors, EPV = 74/8 = 9.25; for the 14 candidate variables from Meta-analysis, EPV ≈ 5.29. Both fall below the conventional threshold of EPV ≥ 20. No formal sample size calculation is reported. |

**Domain 1 overall RoB: H**

**Rationale**: D1.4 is rated PN — the EPV is seriously insufficient relative to the number of modeling variables, with high risk of overfitting. D1.2 is rated PY due to unquantified missing-data exclusions and sampling bias from 1:3 random sampling. The overall risk of bias is high.

**Domain 1 Applicability: H**

This is a single-center retrospective study only involving neurosurgical patients from a tertiary hospital in Shanxi Province. The 1:3 sampling design further limits the representativeness of the sample. The generalisability to other regions, different levels of hospitals and broader neurosurgical populations is limited.

**Domain 2 — Predictors**

|  | **Signalling Question** | **Response** | **Justification** |
| --- | --- | --- | --- |
| D2.1 | Were predictors defined and assessed in a similar way for all participants? | PY | All predictors were retrospectively extracted from medical records with a unified data collection approach. However, no standardized data dictionary, detailed variable definition rules or inter-rater reliability test were provided, so the measurement consistency cannot be fully confirmed. |
| D2.2 | Were predictor assessments made without knowledge of outcome data? | PY | Fixed or admission-based variables (sex, age, baseline diabetes, emergency admission) are less affected by knowledge of outcome. However, ICU admission, prophylactic antibiotic use and D-dimer are time-varying variables accumulated/measured during hospitalization, overlapping with the outcome occurrence window. No blinding of data extraction to outcome status was stated in this retrospective study. |
| D2.3 | Are all predictors available at the time the model is intended to be used? | N | The study claims the model is for early warning of CAUTI. Among the 8 final predictors, female sex, age ≥60 years, diabetes, emergency admission and consciousness disorder on admission can be obtained at catheter insertion. However, ICU admission and prophylactic antibiotic use are time-varying variables determined during hospitalization, which are fundamentally unknowable at the initial early warning point of catheterization. There is a typical temporal leakage problem that undermines the early warning value of the model. |

**Domain 2 overall RoB: H**

**Rationale**: D2.3 is rated N. Core predictors such as ICU admission and prophylactic antibiotic use cannot be obtained at the intended early warning time point, resulting in temporal leakage. D2.1 and D2.2 are rated PY with unclear measurement consistency and blinding. The overall risk of bias is high.

**Domain 2 Applicability: L**

All 8 final predictors are routine demographic, medical history, treatment and laboratory indicators in neurosurgical clinical practice. No proprietary instruments or special tests are required, and the clinical accessibility is high.

**Domain 3 — Outcome**

|  | **Signalling Question** | **Response** | **Justification** |
| --- | --- | --- | --- |
| D3.1 | Was the outcome determined appropriately? | PY | CAUTI was diagnosed according to the 2001 *Diagnostic Criteria for Nosocomial Infection (Trial)* and the 2010 *Technical Guidelines for Prevention and Control of Catheter-associated Urinary Tract Infection (Trial)* issued by the Ministry of Health of China, which are authoritative domestic standards. However, the specific symptom criteria and bacterial count thresholds were not enumerated in the text, so full compliance cannot be verified. |
| D3.2 | Was a pre-specified or standard outcome definition used? | PY | The official domestic CAUTI diagnostic standard was adopted as a standardized outcome definition, but the specific implementation details of the diagnostic criteria were not fully reported. |
| D3.3 | Were predictors excluded from the outcome definition? | Y | The CAUTI outcome was defined by clinical symptoms and microbiological evidence. None of the 8 final predictors are components of the outcome diagnostic criteria, and there is no definitional overlap bias. |
| D3.4 | Was the outcome assessed without knowledge of predictor values? | U | No information was provided on whether the staff who determined CAUTI status were blinded to predictor values. Blinding cannot be confirmed in this retrospective study. |
| D3.5 | Was the time interval between predictor assessment and outcome determination appropriate? | N | Core time-varying predictors such as ICU admission and prophylactic antibiotic use share the same observation window as the CAUTI outcome. The study did not explicitly limit predictor assessment to before the outcome occurs, and reverse causality is possible: patients who develop CAUTI may be transferred to the ICU as a consequence of the infection, meaning ICU admission may be a result rather than a cause of CAUTI. |
| D3.6 | Was there a sufficient proportion of participants with the outcome? | PY | There were 74 CAUTI cases with an infection rate of 24.67%. The absolute number of events is moderate among domestic single-center neurosurgical CAUTI studies. However, the 1:3 sampling artificially increased the outcome proportion, and the event count is still insufficient relative to the number of modeling variables. |

**Domain 3 overall RoB: H**

**Rationale**: D3.5 is rated N — core time-varying predictors overlap with the outcome window, with unclear causal sequence and temporal confounding. D3.1 and D3.2 are rated PY due to incomplete reporting of outcome definition details. D3.4 cannot be confirmed. The overall risk of bias is high.

**Domain 3 Applicability: L**

The outcome adopts the official CAUTI diagnostic standard commonly used in domestic clinical practice, consistent with real-world clinical practice. The 24.67% infection rate is in line with the characteristics of severe neurosurgical patients with long-term catheterization.

**Domain 4 — Analysis**

|  | **Signalling Question** | **Response** | **Justification** |
| --- | --- | --- | --- |
| D4.1 | Were there an appropriate number of participants with the outcome? | PN | With 74 CAUTI events in the derivation set and 8 final predictors, EPV = 9.25; with 14 candidate variables, EPV ≈ 5.29. Both are significantly below the conventional threshold of EPV ≥ 20. The number of outcome events is insufficient relative to modeling variables. |
| D4.2 | Were continuous and categorical predictors handled appropriately? | PN | Most continuous variables (age, catheter duration, hospital stay, etc.) were dichotomized, losing original information and introducing threshold instability. No linearity assessment on the log-odds scale was reported, and most cut-points were only based on clinical convention without methodological justification. Only D-dimer was included as a continuous variable. |
| D4.3 | Were all enrolled participants included in the analysis? | PY | All 300 enrolled patients were included in the analysis with clear sample size. However, the total number of initially eligible patients and the number excluded for incomplete data were not reported, so the integrity of enrollment cannot be confirmed. |
| D4.4 | Were missing data handled appropriately? | U | Patients with incomplete data were directly excluded as an eligibility criterion. The number of excluded cases, distribution of missing variables and missing pattern were not reported, and the impact of missing data on results cannot be evaluated. |
| D4.5 | Was selection of predictors based on univariable analysis avoided? | N | Variables with P<0.2 in univariate analysis were entered into multivariate logistic regression. This two-stage univariable-then-multivariable selection approach is a well-recognised source of optimism and selection bias. |
| D4.6 | Were complexities in the data accounted for appropriately? | U | No clustering effects (e.g., surgical team, ward unit) or temporal trends during the study period (March 2018 to April 2020, covering the early stage of the COVID-19 pandemic) were addressed. Potential changes in infection control protocols and patient mix were not considered as confounders. |
| D4.7 | Were relevant model performance measures evaluated appropriately? | PN | Only discrimination was evaluated via ROC curve, AUC, sensitivity and specificity. No calibration metrics (e.g., Hosmer-Lemeshow test, calibration curve) or clinical utility measures (e.g., decision curve analysis) were reported. The performance evaluation dimension is incomplete. |
| D4.8 | Were model overfitting and optimism accounted for? | PN | The model was built and tested on a single dataset without internal validation (bootstrap, cross-validation) or shrinkage correction of regression coefficients. No external validation was performed. The risk of overfitting and optimism cannot be evaluated or controlled. |
| D4.9 | Do predictors and their assigned weights correspond to the multivariable analysis results? | Y | The β coefficients, standard errors, ORs and 95% CIs of all predictors were clearly reported, and the prediction equation was constructed based on the regression coefficients. The predictor weights are fully consistent with the multivariate analysis results with internal logical consistency. |
| D4.10 | Was model performance evaluated in different subgroups of participants? | N | No subgroup performance analyses by disease type, age stratification or consciousness disorder severity were reported. The stability of model performance across different population subgroups cannot be assessed. |
| D4.11 | Was model performance evaluated using calibration? | N | No calibration evaluation was performed. Neither the Hosmer-Lemeshow goodness-of-fit test nor calibration curve was provided. The consistency between predicted probability and actual occurrence probability cannot be confirmed. |
| D4.12 | Was model performance evaluated in an external validation dataset? | N | The study was modeled on a single-center single sample. Neither internal validation set nor independent external cohort validation was conducted. The generalisation performance of the model has not been verified. |

**Domain 4 overall RoB: H**

**Rationale**: Two-stage univariable pre-screening (D4.5 = N); severely insufficient EPV (D4.1 = PN); no calibration evaluation (D4.11 = N); no internal/external validation and no overfitting correction (D4.8 = PN, D4.12 = N); improper dichotomization of continuous variables (D4.2 = PN). There are multiple methodological deficiencies, and the overall risk of bias is high. The internal consistency between model weights and regression results (D4.9 = Y) is a relative strength but cannot offset the fundamental defects.

**Overall Judgment Summary**

| Domain | Risk of Bias | Applicability |
| --- | --- | --- |
| D1 Participants | H | H |
| D2 Predictors | H | L |
| D3 Outcome | H | L |
| D4 Analysis | H | — |
| Overall | H | H |

**PROBAST Assessment: Hur Eun Young 2019**

**Report:** Hur EY, Jin Y, Jin T, Lee SM. Development and Evaluation of the Automated Risk Assessment System for Catheter-Associated Urinary Tract Infection. *CIN: Computers, Informatics, Nursing*. 2019;37(9):463–472.

**Domain 1 — Participants**

|  | **Signalling Question** | **Response** | **Justification** |
| --- | --- | --- | --- |
| D1.1 | Were appropriate data sources used? | Y | Data were retrospectively extracted from the electronic health record (EHR) system of a 1356-bed tertiary teaching hospital in Seoul, South Korea. The institutional EHR captures comprehensive demographic, laboratory and clinical data, and represents an appropriate data source for hospitalised patient cohorts. |
| D1.2 | Were all inclusions and exclusions of participants appropriate? | PN | Cases were defined as hospitalised patients with indwelling urinary catheters and positive urine culture (≤2 species, ≥10⁵ CFU/mL) occurring on or after hospital day 3. However, controls were randomly selected from hospitalised patients who *never had indwelling urinary catheters* and had negative urine cultures (<10³ CFU/mL), rather than from catheterised patients without CAUTI. This control selection fundamentally misaligns with the target population for a CAUTI prediction model — all catheterised inpatients — as the presence of a catheter itself is the strongest known risk factor for UTI. The number of patients excluded during eligibility screening is not reported, and no participant flow diagram is provided. |
| D1.3 | Were participants enrolled at a similar and relevant point in their disease or condition? | PN | Cases were enrolled at the time of CAUTI diagnosis, while controls were sampled at random points during their hospital stay. Because the control group had no indwelling catheter, the two groups entered the analysis at fundamentally different baseline risk levels. There is no uniform time zero (e.g., catheter insertion) for risk prediction, introducing prevalent case bias. |
| D1.4 | Was the sample size appropriate? | PN | The development dataset included 2150 patients (297 CAUTI cases, 1208 controls). With 8 final predictors in the logistic model, the events per variable (EPV) ratio is 37.1, which exceeds the conventional minimum threshold of 20. Additional temporal validation sets (n = 1265 in 2011–2012, n = 1000 in 2014) provide further outcome numbers. However, no *a priori* sample size calculation is reported. Furthermore, the 1:4 case-control sampling artificially inflates the event proportion and does not reflect the true baseline risk of CAUTI among catheterised patients. |

**Domain 1 overall RoB: H**

**Rationale:** D1.2 is rated PN due to the critical flaw of selecting non-catheterised patients as controls, which means the model discriminates catheterised from non-catheterised patients rather than predicting infection risk within the catheterised population. D1.3 and D1.4 are also rated PN due to non-uniform baseline timing and lack of sample size justification. Together, these issues place the domain at high risk of bias.

**Domain 1 Applicability: H**

The study uses a case-control design with non-catheterised controls, which does not reflect the clinical target population of patients with indwelling urinary catheters for whom CAUTI risk stratification would be performed. All data come from a single Korean tertiary teaching hospital, limiting generalisability to other settings. The model may be applicable to broad inpatient UTI surveillance but not to targeted CAUTI prediction in catheterised cohorts.

**Domain 2 — Predictors**

|  | **Signalling Question** | **Response** | **Justification** |
| --- | --- | --- | --- |
| D2.1 | Were predictors defined and assessed in a similar way for all participants? | PY | Eight final predictors were extracted from the EHR system: length of stay, ICU admission, dependent physical activity, highest neutrophil percentage, lowest serum sodium <136 mEq/L, lowest serum albumin <3.5 g/dL, highest blood urea nitrogen >20 mg/dL, and total indwelling catheter duration. All variables are objectively documented in routine clinical records. However, variable selection was performed using weight-of-evidence (WOE) and information value (IV) methods from an initial pool of 4200 variables, with no explicit data dictionary or inter-rater reliability assessment reported. Dichotomisation thresholds for laboratory variables are based on clinical reference ranges rather than data-driven optimal cut-points. |
| D2.2 | Were predictor assessments made without knowledge of outcome data? | PY | Most laboratory and demographic variables were recorded prospectively in the EHR during routine care, prior to CAUTI diagnosis. However, total length of stay and total catheter duration are cumulative end-of-stay variables that are finalised after the outcome is known. In this retrospective analysis, these values were abstracted with full knowledge of case/control status. Additionally, the AutoRAS-UTI system incorporates default rules based on urine microscopy and culture results, which are directly related to the outcome. |
| D2.3 | Are all predictors available at the time the model is intended to be used? | N | Two of the eight core predictors — total length of stay and total indwelling urinary catheter duration — represent the *entire hospital stay* cumulative values used to fit the model, which cannot be known at the time of catheter insertion or during early hospitalisation. Although the AutoRAS-UTI system updates risk scores daily using accumulated values up to each day, the regression coefficients were trained on final total durations, meaning the model is not calibrated for partial-duration inputs. Furthermore, catheter duration and length of stay are both on the causal pathway with CAUTI: infection itself prolongs hospitalisation and catheterisation, creating reverse causality. The default high-risk rules (urine WBC ≥3, bacteria on microscopy, urine culture ≥10³ CFU/mL) represent early markers of the outcome itself, not pre-infection predictors. |

**Domain 2 overall RoB: H**

**Rationale:** D2.3 is rated N. Two core continuous predictors (total hospital stay, total catheter duration) are end-of-stay variables unknowable at clinically relevant early time points, and reverse causality inflates their apparent predictive strength. Default system rules further incorporate outcome-adjacent laboratory markers. D2.1 and D2.2 are rated PY due to high-throughput data-driven variable selection and retrospective ascertainment of cumulative variables.

**Domain 2 Applicability: L**

All eight core predictors are routinely captured in standard EHR systems and require no specialised or proprietary assays. The automated system integrates directly with existing clinical workflows, making the implementation approach highly transportable to other hospitals with mature EHR infrastructure.

**Domain 3 — Outcome**

|  | **Signalling Question** | **Response** | **Justification** |
| --- | --- | --- | --- |
| D3.1 | Was the outcome determined appropriately? | PY | The study adopted the CDC/NHSN surveillance definition of CAUTI as the reference framework and required urine culture with ≤2 species at ≥10⁵ CFU/mL on or after hospital day 3. However, clinical symptoms (fever, suprapubic pain, costovertebral tenderness) were not required for case ascertainment, meaning cases may include asymptomatic bacteriuria rather than true symptomatic CAUTI. This broadens the case definition beyond standard clinical and surveillance criteria. |
| D3.2 | Was a pre-specified or standard outcome definition used? | PY | The study references the standard CDC/NHSN CAUTI definition but applies only the microbiological component, omitting the clinical symptom criterion. The definition is therefore partially standardised but not fully aligned with international surveillance standards. |
| D3.3 | Were predictors excluded from the outcome definition? | Y | The core eight model predictors (demographic, admission, laboratory and duration variables) are not components of the CAUTI diagnostic definition. Although the system’s default rules include urine microscopy and culture thresholds, these are add-on decision rules and not variables in the regression model itself. |
| D3.4 | Was the outcome assessed without knowledge of predictor values? | U | Urine cultures were processed by the clinical microbiology laboratory using standard protocols, which are typically performed blinded to other clinical variables. However, no explicit statement of blinding is provided, and retrospective chart abstraction cannot guarantee outcome assessors were masked to predictor values. |
| D3.5 | Was the time interval between predictor assessment and outcome determination appropriate? | N | The two strongest cumulative predictors — total catheter duration and total length of stay — are measured across the entire admission, including the period after CAUTI onset. By definition, a patient diagnosed with CAUTI will have accumulated catheter days up to and beyond the infection event, and will typically have a longer total hospital stay. This concurrent measurement creates severe temporal confounding and reverse causality, where the outcome itself drives higher predictor values rather than the reverse. |
| D3.6 | Was there a sufficient proportion of participants with the outcome? | Y | The development set included 297 CAUTI cases, with 253 and 200 cases in the two validation sets respectively. The absolute number of outcome events is large enough for stable statistical modelling, despite the artificially elevated event proportion from 1:4 case-control sampling. |

**Domain 3 overall RoB: H**

**Rationale:** D3.5 is rated N due to severe temporal confounding and reverse causality between cumulative duration predictors and the infection outcome. D3.1 and D3.2 are rated PY because the outcome definition omits required clinical symptoms and may misclassify asymptomatic bacteriuria as CAUTI. D3.4 is unclear. Collectively, these issues result in high risk of bias in the outcome domain.

**Domain 3 Applicability: L**

The microbiological component of the outcome aligns with standard hospital infection surveillance practice. Although the omission of clinical symptoms deviates from strict CAUTI criteria, urine-culture–based ascertainment is consistent with real-world automated surveillance workflows that rely on laboratory data triggers.

**Domain 4 — Analysis**

|  | **Signalling Question** | **Response** | **Justification** |
| --- | --- | --- | --- |
| D4.1 | Were there an appropriate number of participants with the outcome? | PN | With 297 outcome events in the training set and 8 final predictors, the EPV of 37.1 exceeds the conventional 20-event threshold. However, the initial variable pool contained 4200 candidates, which were filtered via WOE-IV and stepwise selection. This high-dimensional screening process introduces substantial selection bias and optimism, even with adequate EPV for the final model. No formal sample size calculation is presented. |
| D4.2 | Were continuous and categorical predictors handled appropriately? | PN | Some continuous variables (length of stay, catheter duration, neutrophil percentage) entered the model as continuous terms, while laboratory variables (sodium, albumin, BUN) were dichotomised at clinically conventional cut-points. No assessment of linearity on the log-odds scale is reported for continuous predictors. The use of WOE binning for initial variable screening is a credit-scoring approach not standard in clinical prediction modelling, and may overfit to idiosyncrasies in the development data. |
| D4.3 | Were all enrolled participants included in the analysis? | PY | All 2150 patients in the development cohort, 1265 in the algorithm validation cohort, and 1000 in the system validation cohort are included in the reported analyses. However, no participant flow diagram is provided, and the total number of eligible patients approached for inclusion is not stated. The control group was sampled rather than including all eligible non-cases, which is a design choice rather than a reporting gap. |
| D4.4 | Were missing data handled appropriately? | U | The study does not describe the extent of missing data, particularly for laboratory variables such as albumin, sodium and BUN, which are not measured for every inpatient. No imputation strategy or complete-case assumption is explicitly stated. The impact of missing values on model performance cannot be determined. |
| D4.5 | Was selection of predictors based on univariable analysis avoided? | N | Predictor selection followed a multi-step data-driven procedure: initial filtering of 4200 variables by information value, further elimination based on prevalence and direction, then forward, backward and stepwise logistic regression to select the final 8 predictors. This purely data-driven variable selection from an extremely large initial candidate pool is a well-recognised source of severe overfitting, model optimism and selection bias. |
| D4.6 | Were complexities in the data accounted for appropriately? | U | The analysis does not explicitly account for clustering by clinical department, nursing unit or treating clinician. Temporal trends across the 6-year study period (2008–2014) are not formally modelled, though separate validation cohorts at different time points provide some reassurance of temporal stability. No adjustment for secular changes in infection control practice or catheter management protocols is reported. |
| D4.7 | Were relevant model performance measures evaluated appropriately? | PY | The study reports standard discrimination metrics including sensitivity, specificity, positive and negative predictive values, accuracy, Youden index and AUC. Discrimination is evaluated across internal split-sample and two temporal validation sets. However, calibration performance (calibration curve, Hosmer-Lemeshow test, calibration slope or Brier score) is not assessed at all, and no decision curve analysis is presented to evaluate clinical net benefit. |
| D4.8 | Were model overfitting and optimism accounted for? | PN | The study uses a 70:30 random split-sample internal validation and two independent temporal validation cohorts (2011–2012 and 2014), which is a relative strength compared with single-sample internal-only studies. AUC only decreases marginally from 0.97 (training) to 0.95 (test) and remains 0.97 in the first temporal validation set. However, no bootstrap resampling, cross-validation or regression coefficient shrinkage is applied. The high-throughput variable selection was likely performed using the full development dataset before splitting, which introduces data leakage and inflates apparent performance. |
| D4.9 | Do predictors and their assigned weights correspond to the multivariable analysis results? | Y | Full logistic regression coefficients, odds ratios and 95% confidence intervals are tabulated for all eight final predictors. The explicit prediction equation is provided in the text, and the risk-scoring algorithm maps directly to the regression results. Internal consistency between model coefficients and the scoring system is confirmed. |
| D4.10 | Was model performance evaluated in different subgroups of participants? | N | No subgroup analyses of discrimination or calibration are reported by age, sex, ICU status, department or comorbidity burden, despite the heterogeneous inpatient population. |
| D4.11 | Was model performance evaluated using calibration? | N | No assessment of calibration is reported. There is no calibration plot, Hosmer-Lemeshow test statistic, calibration slope, intercept or Brier score provided for any of the development or validation datasets. |
| D4.12 | Was model performance evaluated in an external validation dataset? | Y | Two separate temporal external validation datasets are used: 1265 patients from July 2011 to December 2012 for algorithm validation, and 1000 patients from January to December 2014 for system-level validation. Both represent independent time periods from the development cohort (October 2008 to June 2011) and are drawn from the same hospital. No multi-centre external validation is performed. |

**Domain 4 overall RoB: H**

**Rationale:** Data-driven variable selection from 4200 candidate predictors (D4.5 = N), complete absence of calibration assessment (D4.11 = N), no formal shrinkage or bootstrap correction for optimism (D4.8 = PN), and lack of subgroup analysis (D4.10 = N) all contribute to high risk of bias. The presence of temporal external validation and full reporting of discrimination metrics are relative strengths but do not offset fundamental methodological limitations.

**Overall Judgment Summary**

| Domain | Risk of Bias | Applicability |
| --- | --- | --- |
| D1 Participants | H | H |
| D2 Predictors | H | L |
| D3 Outcome | H | L |
| D4 Analysis | H | — |
| Overall | H | H |
