# Supplementary material for: Systematic review of risk prediction models for catheter-associated urinary tract infection
Source: Front Public Health. 2026 Sep 30;14:1884134. doi: 10.3389/fpubh.2026.1884134 (PMC13662166; doi:10.3389/fpubh.2026.1884134)
Supplement: Supplementary file 6 [file Supplementary_file_6.DOCX]

**CHARMS Data Extraction Table**

**Domain 1: Source of Data**

| Item | LIU Huiying 2025 | LI Mengchao 2025 | Sufriyana 2025 | ZHU Rui 2024 | SUN Qing 2024 | WANG Fang 2023 | LIU Yuting 2023 | LI Yuping 2023 | XIA Liyao 2022 | LÜ Yangyang 2022 | LIAO Liping 2022 | LI Zhe 2021 | HUR Eun Young 2019 |
| --- | --- | --- | --- | --- | --- | --- | --- | --- | --- | --- | --- | --- | --- |
| Study design | Retrospective cohort | Retrospective cohort | Retrospective cohort | Retrospective cohort | Retrospective case-control | Retrospective cohort | Retrospective cohort | Retrospective cohort | Retrospective cohort | Retrospective case-control | Retrospective case-control | Retrospective case-control | Retrospective case-control |
| Data source | EHR, single centre | EHR, single centre | Multi-institutional EHR database (TMUCRD, 3 hospitals) | EHR, single centre | EHR + nosocomial infection surveillance system, single centre | EHR, single centre | HIS + infection control office records, single centre | EHR, single centre | Medical records, single centre | EHR, single centre | EHR, single centre | EHR, single centre | EHR, single centre |
| Institution | Zhongshan Hospital of TCM, Guangdong | Affiliated Hospital of Jiujiang University, Jiangxi | Taipei Medical University hospitals, Taiwan | Jiangsu Provincial People's Hospital, Nanjing | Tertiary hospital, Shandong | First Affiliated Hospital of Army Medical University, Chongqing | Subei People's Hospital, Yangzhou University | Subei People's Hospital, Yangzhou University | Zhejiang Cancer Hospital, Hangzhou | Second Affiliated Hospital of Wenzhou Medical University | Chongqing Emergency Medical Center | Tertiary hospital, Shanxi | Teaching hospital, Seoul, South Korea |
| Study period | Jan 2021–Dec 2022 | Jul 2020–Nov 2023 | 1998–2021 | Jan 2014–Jun 2024 | 2017–2021 | Jan 2020–Dec 2021 | Not reported | Jan 2019–Jan 2020 | Not reported | Jun 2018–Dec 2020 | Jan 2019–Dec 2021 | Mar 2018–Apr 2020 | Oct 2008–Dec 2014 |
| No. of centres | 1 | 1 | 3 | 1 | 1 | 1 | 1 | 1 | 1 | 1 | 1 | 1 | 1 |
| Ethical approval | Not reported | Not reported | Not reported | Not reported | Not reported | Yes | Not reported | Not reported | Yes | Not reported | Not reported | Not reported | Not reported |
| Country | China | China | Taiwan | China | China | China | China | China | China | China | China | China | South Korea |
| Language | Chinese | Chinese | English | Chinese | Chinese | English | English | English | Chinese | Chinese | Chinese | Chinese | English |
| Publication type | Journal article | Journal article | Journal article | Journal article | Master's thesis | Journal article | Journal article | Journal article | Journal article | Journal article | Journal article | Master's thesis | Journal article |

**Domain 2: Participants**

| Item | LIU Huiying 2025 | LI Mengchao 2025 | Sufriyana 2025 | ZHU Rui 2024 | SUN Qing 2024 | WANG Fang 2023 | LIU Yuting 2023 | LI Yuping 2023 | XIA Liyao 2022 | LÜ Yangyang 2022 | LIAO Liping 2022 | LI Zhe 2021 | HUR Eun Young 2019 |
| --- | --- | --- | --- | --- | --- | --- | --- | --- | --- | --- | --- | --- | --- |
| Target population | Stroke inpatients with indwelling catheter | SCI surgical patients with indwelling catheter | All catheterised inpatients aged 20–75 | Elderly (≥65) critically ill ICU patients | Hospitalised catheterised patients | Post-radical hysterectomy cervical cancer patients | NICU catheterised patients | NICU catheterised patients | Post-op gynaecological malignancy patients | Emergency department catheterised patients | TBI patients with indwelling catheter | Neurosurgical patients with indwelling catheter | Hospitalised catheterised patients |
| Key inclusion criteria | Confirmed stroke, age ≥18, negative urinalysis at 48 h post-admission, indwelling catheter | Imaging-confirmed SCI, surgical treatment, negative urine culture at admission, age >18 | Age 20–75, urinary catheterisation prescribed | Age ≥65, CAUTI per 2010 MoH guideline, APACHE II ≥25 | Hospitalised with indwelling catheter, CAUTI met standard criteria | Age ≥18, cervical SCC or adenocarcinoma, type C radical hysterectomy | Age ≥18, NICU admission, new indwelling catheter >48 h, negative baseline urine culture | Age ≥18, NICU admission with indwelling catheter, no UTI before admission | Age ≥18, gynaecological malignancy, postoperative transurethral catheter, no mental illness | Indwelling catheter in ED, negative urinalysis at catheter insertion | Age 18–85, TBI per CT/MRI, negative urine culture on admission, catheter ≥48 h | Neurosurgical patients with indwelling catheter, age ≥18, complete clinical data | Indwelling catheter with positive urine culture ≥10⁵ CFU/mL on or after hospital day 3 (cases); non-catheterised patients with negative culture (controls) |
| Key exclusion criteria | TIA/CVST only, hospitalisation ≤2 days, UTI at admission, incomplete data | Spinal shock, cardiopulmonary dysfunction, urinary malformation, incomplete data | / | UTI unrelated to catheter, death within 48 h of ICU admission, incomplete data | Pre-admission catheterisation, pre-existing UTI, severely incomplete records | Pre-op indwelling catheter, intraoperative urinary injury, kidney disease, kidney transplant, post-op intermittent catheterisation | Age <18, NICU stay <48 h, no indwelling catheter, positive baseline urine culture | UTI before admission, NICU stay <48 h, missing data (n=47) | Pre-op indwelling catheter, pre-existing UTI, serious cardiopulmonary/brain/renal disease, recurrence during treatment, second surgery required | Malignant tumour, blood/autoimmune diseases, pre-existing infection, mental/intellectual disability, incomplete data | Severe vital organ damage, abnormal coagulation, severe autoimmune/endocrine disease, UTI on admission, incomplete data | Pre-admission UTI or other infection, pregnancy/lactation | / |
| Recruitment method | Consecutive (implied) | Consecutive (implied) | All eligible episodes in database | Consecutive over 10 years | Retrospective case identification with 1:1 matching | Consecutive (implied) | Consecutive | Consecutive | Convenience sampling | Consecutive (implied) | Consecutive (implied) | 1:3 random sampling | 1:4 case-control sampling |
| Total N | 699 | 140 | 122,386 episodes | 8,905 | 1,042 | 380 | 537 | 537 | 800 | 1,780 | 815 | 300 | 4,060 (development + validation) |
| Total CAUTI events | 84 | 32 | 17,506 | 114 | 521 | 88 | 21 | 21 | 163 | 142 | 121 | 74 | 816 |
| Overall event rate | 12.0% | 22.9% | 14.3% (training) | 1.28% | 50.0% (by design) | 23.2% | 3.9% | 3.9% | 20.4% | 7.98% | 14.85% | 24.7% | / |
| Dataset split | Development 70% / internal validation 30% (random) | Development only | Hospital-based split: development (Shuang Ho + TMU hospitals) / external test (Wanfang hospital, n=21,123); development set randomly split 80:20 into training (n=81,010) and internal validation (n=20,253) | Development only (bootstrap validation) | Development only (bootstrap validation) | Development only (bootstrap validation) | Development only | Development 389 / internal validation 148 (random) | Development 600 / internal validation 200 (random 3:1) | Development only | Development 570 / internal validation 245 (random 7:3) | Development only | Development 2,150 / internal validation 645 / temporal external validation 1,265 + 1,000 |
| Development N (events) | 489 (57) | 140 (32) | 81,010 (10,676) | 8,905 (114) | 1,042 (521) | 380 (88) | 537 (21) | 389 (17) | 600 (124) | 1,780 (142) | 570 (80) | 300 (74) | 2,150 (297) |
| Internal validation N (events) | 210 (27) | / | 20,253 (2,756) | / | / | / | / | 148 (4) | 200 (39) | / | 245 (41) | / | 645 (133) |
| External validation N (events) | / | / | 21,123 (4,074) | / | / | / | / | / | / | / | / | / | 1,265 (253) + 1,000 (200) |
| A priori sample size calculation | No | No | No | No | No | No | No | No | No | No | No | No | No |

**Domain 3: Outcome**

| Item | LIU Huiying 2025 | LI Mengchao 2025 | Sufriyana 2025 | ZHU Rui 2024 | SUN Qing 2024 | WANG Fang 2023 | LIU Yuting 2023 | LI Yuping 2023 | XIA Liyao 2022 | LÜ Yangyang 2022 | LIAO Liping 2022 | LI Zhe 2021 | HUR Eun Young 2019 |
| --- | --- | --- | --- | --- | --- | --- | --- | --- | --- | --- | --- | --- | --- |
| Outcome | CAUTI | CAUTI | CAUTI (administratively derived) | CAUTI + CAASB | CAUTI | Post-op UTI (CAUTI) | CAUTI | CAUTI | CAUTI | CAUTI | CAUTI | CAUTI | CAUTI |
| Diagnostic criteria source | Unspecified clinical/temporal definition | IDSA 2009 | Administrative: UTI diagnosis code or urine culture order, without concurrent pneumonia | 2010 Chinese MoH guideline (includes CAASB) | 2010 Chinese MoH guideline | IDSA 2009 (symptoms + ≥10³ CFU/mL) | NHSN/CDC 2015 (partial: culture ≥10⁵ CFU/mL + fever only; symptom criteria omitted) | NHSN/CDC (partial: culture ≥10⁵ CFU/mL only; symptom criteria omitted) | 2010 Chinese MoH guideline (symptom + culture, organism-specific thresholds) | 2010 Chinese MoH guideline (reference only; specific criteria not enumerated) | 2010 Chinese MoH guideline (reference only; specific criteria not enumerated) | 2001 Nosocomial Infection Criteria + 2010 Chinese MoH guideline | CDC/NHSN (partial: culture ≥10⁵ CFU/mL ≤2 species; symptom criteria omitted) |
| Standard definition used | No (incomplete) | Partial (IDSA cited, criteria not enumerated) | No (non-standard surrogate) | Partial (CAASB included) | Yes | Yes (most complete in series) | Partial (symptom criteria omitted) | Partial (symptom criteria omitted) | Yes (most detailed Chinese-standard) | Partial (criteria not enumerated) | Partial (criteria not enumerated) | Partial (criteria not enumerated) | Partial (symptom criteria omitted) |
| Outcome ascertainment method | Retrospective EHR extraction | Retrospective EHR extraction | Administrative code and culture order extraction | Retrospective EHR extraction | EHR + infection surveillance system | Retrospective EHR + telephone follow-up | Retrospective EHR + infection control records | Retrospective EHR + routine urine culture every 3 days | Retrospective EHR + telephone follow-up post-discharge | Retrospective EHR | Retrospective EHR | Retrospective EHR | Retrospective EHR |
| Intended prediction time point | Hospital admission | Catheter insertion / hospital admission | Each day of catheterisation episode (daily repeated-measures) | ICU admission / catheter insertion | Catheter insertion / hospital admission | Time of surgery / catheter insertion | NICU admission | NICU admission | Postoperative catheter insertion | Catheter insertion (ED) | Hospital / ICU admission | Catheter insertion (neurosurgery ward) | Daily updated during hospitalisation |
| Prediction horizon | Admission → CAUTI diagnosis or catheter removal | Catheter insertion → CAUTI during hospitalisation | Each assessment day → CAUTI within 3-day span over 15-day range | ICU admission → CAUTI during ICU stay | Catheter insertion → CAUTI during hospitalisation | Catheter insertion → UTI until bladder residual <100 mL | NICU admission → CAUTI during NICU stay | NICU admission → CAUTI during NICU stay | Catheter insertion → CAUTI during hospitalisation and post-discharge follow-up | Catheter insertion → CAUTI during ED stay | Hospital/ICU admission → CAUTI during hospitalisation | Catheter insertion → CAUTI during hospitalisation | Hospital admission → CAUTI on or after hospital day 3 |
| Outcome blinding | Not reported (U) | Not reported (U) | Yes (administrative coding independent of research) | Not reported (U) | Not reported (U) | Not reported (U) | Not reported (U) | Not reported (U) | Not reported (U) | Not reported (U) | Not reported (U) | Not reported (U) | Not reported (U) |

**Domain 4: Candidate Predictors**

| Item | LIU Huiying 2025 | LI Mengchao 2025 | Sufriyana 2025 | ZHU Rui 2024 | SUN Qing 2024 | WANG Fang 2023 | LIU Yuting 2023 | LI Yuping 2023 | XIA Liyao 2022 | LÜ Yangyang 2022 | LIAO Liping 2022 | LI Zhe 2021 | HUR Eun Young 2019 |
| --- | --- | --- | --- | --- | --- | --- | --- | --- | --- | --- | --- | --- | --- |
| No. of candidate variables (as reported) | 8 | 10 | 19 (30 indicators) | 11 | 18 | 19 | 16 | 12 (passing P<0.25 threshold) | 6 (entering MV step; total survey instrument larger) | 7 (entering MV step) | 7 | 14 | 4,200 (initial pool) → 8 (final) |
| No. of final model predictors | 5 | 5 | 14 | 5 | 4 | 5 | 9 (CRT); 6 (scoring model) | 4 | 4 | 5 | 5 | 8 | 8 |
| Final model predictors | Age, NIHSS score, catheter dwell time, ICU stay, combined antibiotic use | Stress hyperglycaemia, catheter dwell time, neurogenic bladder, ASIA score, serum albumin | Age, ICU stay ≥10d, hospital stay ≥4d, sex, DM, immobility, hypertension, diabetic nephropathy, HIV, malignancy, urinary disorders, >2 UTIs in 6 months, neurological disorders, catheter indication | Age, consciousness status, renal dysfunction, catheter dwell time, no. of catheterisations | Catheter days ≥7, ICU stay ≥7d, neurological disease, ≥3 antibiotic types | Age, preoperative chemotherapy history, UTI history, re-catheterisation, catheter dwell time | Age, GCS, seizure on admission, summer admission, mechanical ventilation, antibiotic types, albumin, female sex, catheter dwell time | Age, NICU LOS >30d, seizure on admission, serum albumin | Age ≥60, intraoperative urinary injury, postoperative bladder irrigation, catheter dwell time >7d | Age ≥60, BMI ≥30, catheter dwell time ≥3d, HbA1c, serum albumin | Age ≥60, GCS ≤8, catheter dwell time ≥30d, ICU stay ≥14d, stress hyperglycaemia | Female sex, age ≥60, diabetes, emergency admission, prophylactic antibiotics, consciousness disorder, ICU admission, D-dimer | LOS, ICU admission, physical activity dependence, highest neutrophil %, lowest Na <136, lowest albumin <3.5, highest BUN >20, catheter dwell time |
| Time-varying predictors in final model | Catheter dwell time; combined antibiotic use | Catheter dwell time; antibiotic use duration | Catheter dwell time (by design, daily); hospital stay ≥4d | Catheter dwell time; no. of catheterisations | Catheter days ≥7; ICU stay ≥7d; ≥3 antibiotic types | Catheter dwell time ≥14d; re-catheterisation (partial) | Catheter dwell time (CRT tree variable); NICU LOS (time-varying, not in final scoring model but present in derivation tree) | NICU LOS >30d | Catheter dwell time >7d; postoperative bladder irrigation (partial) | Catheter dwell time ≥3d | Catheter dwell time ≥30d; ICU stay ≥14d | ICU admission; prophylactic antibiotic use | Total LOS; total catheter duration |
| Temporal leakage risk | High | High | Low (by design) | High | High | High | High | High (most severe: OR=272.2) | High | High | High (broadest: 2 of 5 predictors) | High | High |
| Predictor measurement method | Retrospective EHR extraction | Retrospective EHR extraction | Standardised database extraction | Retrospective EHR extraction | Retrospective EHR extraction | Self-designed survey form, dual data entry | Retrospective EHR extraction, double data entry | Retrospective EHR extraction, 3 independent researchers | Retrospective EHR + telephone follow-up | Retrospective EHR extraction | Retrospective EHR extraction | Retrospective EHR extraction | Retrospective EHR extraction |
| Predictor blinding to outcome | Not reported | Not reported | Yes (database extraction) | Not reported | Not reported | No (survey completed after catheter removal, outcome known) | Not reported | Not reported | Not reported | Not reported | Not reported | Not reported | Partial (cumulative variables abstracted with outcome knowledge) |

**Domain 5: Sample Size**

| Item | LIU Huiying 2025 | LI Mengchao 2025 | Sufriyana 2025 | ZHU Rui 2024 | SUN Qing 2024 | WANG Fang 2023 | LIU Yuting 2023 | LI Yuping 2023 | XIA Liyao 2022 | LÜ Yangyang 2022 | LIAO Liping 2022 | LI Zhe 2021 | HUR Eun Young 2019 |
| --- | --- | --- | --- | --- | --- | --- | --- | --- | --- | --- | --- | --- | --- |
| Development events | 57 | 32 | 10,676 | 114 | 521 | 88 | 21 | 17 | 124 | 142 | 80 | 74 | 297 |
| Candidate variables (denominator for EPV) | 8 | 10 | 19 | 11 | 10 | 19 | 16 | 12 | 6 (MV step; full pool larger) | 7 (MV step; full pool larger) | 7 | 14 | 8 (final; initial pool 4,200) |
| EPV (candidate) | 7.1 | 3.2 | >700 | 10.4 | 52.1 | 4.6 | 1.3 | 1.4 | 20.7 (inflated by pre-screening) | 20.3 (inflated by pre-screening) | 11.4 | 5.3 | 37.1 (severely inflated by data-driven selection from 4,200) |
| EPV benchmark applied | LR: ≥10–20 | LR: ≥10–20 | ML: not applicable; assessed by absolute N + regularisation + external validation | LR: ≥10–20 | LR (conditional): ≥10–20 | LASSO: ≥100 | Decision tree: not applicable; assessed by absolute events (n=21), tree depth, node stability | LR: ≥10–20 | LR: ≥10–20 | LR: ≥10–20 | LR: ≥10–20 | LR: ≥10–20 | LR: ≥10–20 (data-driven selection from 4,200 candidates severely inflates optimism regardless) |
| Sample size adequacy | Inadequate (EPV 7.1 < 10) | Critically inadequate (EPV 3.2) | Adequate | Marginal (EPV 10.4, borderline) | Adequate (events), but design distorts baseline risk | Critically inadequate (EPV 4.6; LASSO requires ≥100) | Critically inadequate (21 events; EPV 1.3) | Critically inadequate (EPV 1.4; 17 training events) | Marginal (EPV inflated by pre-screening; true candidate EPV lower) | Marginal (EPV inflated by pre-screening; no validation) | Inadequate (EPV 11.4 < 20) | Inadequate (EPV 5.3) | Inflated EPV; selection from 4,200 candidates introduces severe optimism |

**Domain 6: Missing Data**

| Item | LIU Huiying 2025 | LI Mengchao 2025 | Sufriyana 2025 | ZHU Rui 2024 | SUN Qing 2024 | WANG Fang 2023 | LIU Yuting 2023 | LI Yuping 2023 | XIA Liyao 2022 | LÜ Yangyang 2022 | LIAO Liping 2022 | LI Zhe 2021 | HUR Eun Young 2019 |
| --- | --- | --- | --- | --- | --- | --- | --- | --- | --- | --- | --- | --- | --- |
| Missing data reported | No | No | Partial (predictors with MNAR excluded) | No | No (exclusion criterion only) | No | Yes (n=7 excluded, 1.3%) | Yes (n=47 excluded, 6.5%) | No | No | No | No | No |
| No. excluded for missing data | Not reported | Not reported | N/A (predictor-level exclusion) | Not reported | Not reported | Not reported | 7 | 47 | Not reported | Not reported | Not reported | Not reported | Not reported |
| Handling method | Complete-case exclusion (unquantified) | Complete-case exclusion (unquantified) | Predictors with MNAR pattern excluded at selection stage; remaining missing values handled within ML pipeline | Complete-case exclusion (unquantified) | Complete-case exclusion (unquantified) | Complete-case exclusion (unquantified, embedded in eligibility) | Complete-case exclusion (n=7, quantified) | Complete-case exclusion (n=47, quantified) | Complete-case exclusion (unquantified, embedded in eligibility) | Complete-case exclusion (unquantified) | Complete-case exclusion (unquantified) | Complete-case exclusion (unquantified) | Not described |
| Multiple imputation used | No | No | No | No | No | No | No | No | No | No | No | No | No |
| Missing data adequacy | Inadequate | Inadequate | Partial | Inadequate | Inadequate | Inadequate (U) | Acceptable (transparent) | Inadequate (47 excluded without justification) | Inadequate (U) | Inadequate (U) | Inadequate (U) | Inadequate (U) | Not assessable (U) |

**Domain 7: Model Development**

| Item | LIU Huiying 2025 | LI Mengchao 2025 | Sufriyana 2025 | ZHU Rui 2024 | SUN Qing 2024 | WANG Fang 2023 | LIU Yuting 2023 | LI Yuping 2023 | XIA Liyao 2022 | LÜ Yangyang 2022 | LIAO Liping 2022 | LI Zhe 2021 | HUR Eun Young 2019 |
| --- | --- | --- | --- | --- | --- | --- | --- | --- | --- | --- | --- | --- | --- |
| Modelling method | Binary logistic regression | Binary logistic regression | Ridge regression, decision tree, random forest, XGBoost, DI-VNN | Binary logistic regression (stepwise backward) | Conditional logistic regression | Binary logistic regression (LASSO then LR) | CHAID + CRT decision trees | Binary logistic regression | Binary logistic regression | Binary logistic regression | Binary logistic regression | Binary logistic regression | Binary logistic regression (stepwise) |
| Variable selection method | Univariable pre-screening (P<0.05) → multivariable | Univariable pre-screening (P<0.05) → multivariable | Structural causal modelling + Cox regression + Benjamini-Hochberg correction | Univariable pre-screening → stepwise backward elimination | Conditional logistic regression with P<0.05 and P<0.1 two-threshold screening | LASSO regression → multivariable LR | Decision tree-based (all 16 variables entered; no prior screening) | Univariable pre-screening (P<0.25) → multivariable | Univariable pre-screening (P<0.05) → multivariable | Univariable pre-screening → backward likelihood | Univariable pre-screening → multivariable | Univariable pre-screening (P<0.2) → multivariable | WOE-IV filtering of 4,200 variables → stepwise logistic regression |
| Continuous predictor handling | Continuous (some) and categorical | Categorical (dichotomised/ordinal) | Categorical (binned) | Categorical (all dichotomised/ordinal) | Categorical (all dichotomised) | Categorical (age and catheter duration categorised) | Categorical (dichotomised) | Categorical (dichotomised/categorised) | Categorical (dichotomised) | Continuous (HbA1c, albumin) + categorical (age, BMI, catheter dwell time) | Categorical (all dichotomised) | Mostly categorical; D-dimer as continuous | Continuous (LOS, catheter duration, neutrophil%) + dichotomised laboratory values |
| Linearity assumption assessed | No | No | N/A (ML) | No | No | No | No | No | No | No | No | No | No |
| Cut-point determination | Clinical convention (unstated) | Clinical convention (unstated) | N/A (categorical coding) | Clinical convention (unstated); likely data-driven | Not specified ("optimal cut-off combined with clinical practice") | Clinical convention (age categories) | Clinical convention | Clinical convention | Clinical convention | BMI: ROC-derived Youden index; others: clinical convention | Clinical convention | Clinical convention | Clinical reference ranges (laboratory values) |
| Shrinkage / penalisation | No | No | Yes (ridge regression C=0.01–10; RF/XGBoost tree pruning) | No | No | Yes (LASSO) | No | No | No | No | No | No | No |
| Interactions / nonlinear terms considered | No | No | Yes (RF, XGBoost inherently) | No | No | No | No | No | No | No | No | No | No |
| Model presentation format | Nomogram | Nomogram | Nomogram (algorithm-agnostic) | Nomogram | Nomogram | Nomogram | Risk scoring system | Nomogram | Risk scoring system | Regression equation | Nomogram | Regression equation | Risk scoring system (AutoRAS-UTI) |
| Full model equation / coefficients reported | Partial (OR table) | Yes (logit equation explicit) | Yes (HR table + SHAP values) | Yes (β + OR table) | Yes (β + OR table) | Partial (HR/OR table; labelling inconsistency) | Tree structure figures | Yes (β + OR table) | Yes (β + score assignment) | Yes (full logit equation: Logit(P) = −4.571 + ...) | Yes (β + OR table) | Yes (β + OR table) | Yes (full equation + OR table) |

**Domain 8: Model Performance — Derivation**

| Item | LIU Huiying 2025 | LI Mengchao 2025 | Sufriyana 2025 | ZHU Rui 2024 | SUN Qing 2024 | WANG Fang 2023 | LIU Yuting 2023 | LI Yuping 2023 | XIA Liyao 2022 | LÜ Yangyang 2022 | LIAO Liping 2022 | LI Zhe 2021 | HUR Eun Young 2019 |
| --- | --- | --- | --- | --- | --- | --- | --- | --- | --- | --- | --- | --- | --- |
| Derivation AUC (95% CI) | 0.857 (0.801–0.914) | 0.993 (0.984–1.000) | RR: 0.752; DT: 0.788; RF: 0.788; XGBoost: 0.787; DI-VNN: 0.611 | 0.802 (0.796–0.809) | 0.802 (0.757–0.847) | 0.853 (0.803–0.902) | CHAID: 0.825; CRT: 0.840 | 0.894 (0.831–0.956) | 0.826 (0.773–0.879) | 0.862 (0.822–0.902) | 0.862 (0.820–0.904) | 0.883 (0.836–0.930) | 0.882 (derivation cohort) |
| Sensitivity (derivation) | Not reported | 96.9% | Not reported | Not reported | 79.3% | Not reported | CHAID: 81.0%; CRT: 90.5% | Not reported | Not reported | Not reported | Not reported | Not reported | Not reported |
| Specificity (derivation) | Not reported | 97.4% | Not reported | Not reported | 81.4% | Not reported | CHAID: 73.2%; CRT: 72.8% | Not reported | Not reported | Not reported | Not reported | Not reported | Not reported |
| Optimal cut-off (derivation) | Not reported | Not reported | Not reported | Not reported | Not reported | Not reported | Not reported | Not reported | Score ≥3 points | Not reported | Score ≥2 points | Not reported | Score ≥3 points |
| Calibration method | H-L test; calibration plot | H-L test | Calibration curve | H-L test; calibration plot | H-L test | H-L test; calibration plot | Not reported | H-L test; calibration plot | H-L test | Not reported | H-L test; calibration plot | H-L test; calibration plot | Not reported |
| Calibration result (derivation) | H-L P=0.847; plot reported | H-L P=0.953 | Calibration curve reported; specific statistics NR | H-L P=0.893; plot reported | H-L P=0.062 | H-L P=0.376; plot reported | N/A | H-L P>0.05; plot reported | H-L P=0.632 | Not reported | H-L P=0.361; plot reported | H-L P=0.078; plot reported | Not reported |
| DCA reported | Yes | Yes | Yes | Yes | Yes | Yes | No | Yes | No | No | Yes | Yes | No |
| Performance reliability assessment | Apparent only; EPV 7.1 limits reliability | Apparent only; AUC 0.993 almost certainly reflects severe overfitting (EPV 3.2) | Apparent; best-performing model (XGBoost AUC 0.788) selected for nomogram presentation | Apparent only; bootstrap validation added | Apparent only; case-control design inflates apparent performance | Apparent only; EPV 4.6 with LASSO severely underpowered | Apparent only; 21 events make tree structure highly unstable | Apparent only; 17 training events make all statistics uninterpretable | Apparent only; pre-screening inflates true candidate count | Apparent only; no validation; LÜ Yangyang 2022 EPV marginally adequate | Apparent only; EPV 11.4 borderline | Apparent only; 1:3 sampling artificially inflates event rate | Apparent only; data-driven selection from 4,200 candidates severely inflates optimism |

**Domain 9: Model Performance — Internal Validation**

| Item | LIU Huiying 2025 | LI Mengchao 2025 | Sufriyana 2025 | ZHU Rui 2024 | SUN Qing 2024 | WANG Fang 2023 | LIU Yuting 2023 | LI Yuping 2023 | XIA Liyao 2022 | LÜ Yangyang 2022 | LIAO Liping 2022 | LI Zhe 2021 | HUR Eun Young 2019 |
| --- | --- | --- | --- | --- | --- | --- | --- | --- | --- | --- | --- | --- | --- |
| Internal validation method | Random 7:3 split | Bootstrap | Random 70:20 split (internal test set) | Bootstrap (1,000 iterations) | Bootstrap (1,000 iterations) | Bootstrap (1,000 iterations) | None | Random split (389 derivation / 148 validation) | Random 3:1 split | None | Random 7:3 split | None | Random 70:15 split |
| Validation N (events) | 210 (27) | N/A | 20,253 (2,756) | N/A (bootstrap) | N/A (bootstrap) | N/A (bootstrap) | N/A | 148 (4) | 200 (39) | N/A | 245 (41) | N/A | 645 (133) |
| Validation AUC (95% CI) | 0.834 (0.748–0.921) | N/A | RR: 0.756; DT: 0.795; RF: 0.792; XGBoost: 0.793; DI-VNN: 0.616 | Bootstrap C-statistic: 0.802 | Bootstrap C-statistic: 0.802 | Bootstrap C-statistic: 0.853 | N/A | 0.888 (0.790–0.986) | 0.821 (0.748–0.895) | N/A | 0.841 (0.774–0.907) | N/A | 0.875 (internal hold-out) |
| Sensitivity (validation) | Not reported | N/A | Not reported | Not reported | Not reported | Not reported | N/A | Not reported | Not reported | N/A | Not reported | N/A | Not reported |
| Specificity (validation) | Not reported | N/A | Not reported | Not reported | Not reported | Not reported | N/A | Not reported | Not reported | N/A | Not reported | N/A | Not reported |
| Calibration (validation) | H-L P=0.732; calibration plot | N/A | Calibration curve reported | Bootstrap-corrected; H-L P=0.893 | Bootstrap-corrected; H-L P=0.062 | Bootstrap-corrected; H-L P=0.376 | N/A | H-L P>0.05; MAE=0.012; calibration plot | H-L P=0.789; calibration plot | N/A | H-L P=0.206; calibration plot | N/A | Not reported |
| DCA (validation) | Yes | N/A | Yes | Yes (bootstrap) | Yes (bootstrap) | Yes (bootstrap) | N/A | Yes | No | N/A | Yes | N/A | No |
| Validation reliability notes | Adequate event count (n=27); random split susceptible to optimism | N/A | Adequate events; random split susceptible to optimism | Bootstrap corrects for optimism but remains within development population | Bootstrap corrects for optimism but remains within development population; case-control design distortion persists | Bootstrap corrects for optimism but remains within development population; EPV 4.6 severely underpowered for LASSO | N/A | Critically unreliable: only 4 CAUTI events in validation set; all statistics statistically uninformative | Adequate events; random split susceptible to optimism | N/A | Adequate events; random split susceptible to optimism | N/A | Remains within same institution; temporal contamination possible |

**Domain 10: Model Performance — External Validation**

| Item | Sufriyana 2025 | HUR Eun Young 2019 | All other reports |
| --- | --- | --- | --- |
| External validation type | Geographic / cross-institutional | Temporal (same institution, different period) | No external validation |
| Validation institution | External hospital, Taiwan (institution not named) | Same Seoul teaching hospital: algorithm validation 2011–2012; system validation 2014 | N/A |
| Validation N (events) | 21,123 (4,074) | 1,265 (253) + 1,000 (200) | N/A |
| Validation AUC (95% CI) | XGBoost: 0.758; RF: 0.751; RR: 0.737; DT: 0.731; DI-VNN: 0.605 | Temporal: 0.970; Prospective: 0.970 | N/A |
| Sensitivity (external) | Not reported | Not reported | N/A |
| Specificity (external) | Not reported | Not reported | N/A |
| Calibration (external) | Calibration curve reported; specific statistics NR | Not reported | N/A |
| DCA (external) | Yes | No | N/A |
| Key interpretation note | Performance degradation from internal test (XGBoost 0.793 → 0.758) is modest; remains the only cross-institutional external validation in this series | Temporal external AUC 0.970 almost certainly reflects data leakage from cumulative end-of-stay predictors and same-institution training; not interpretable as evidence of true generalisability | N/A |

**Domain 11: Results Summary and Model Availability**

| Item | LIU Huiying 2025 | LI Mengchao 2025 | Sufriyana 2025 | ZHU Rui 2024 | SUN Qing 2024 | WANG Fang 2023 | LIU Yuting 2023 | LI Yuping 2023 | XIA Liyao 2022 | LÜ Yangyang 2022 | LIAO Liping 2022 | LI Zhe 2021 | HUR Eun Young 2019 |
| --- | --- | --- | --- | --- | --- | --- | --- | --- | --- | --- | --- | --- | --- |
| Nomogram / tool provided | Yes (nomogram) | Yes (nomogram) | Yes (nomogram) | Yes (nomogram) | Yes (nomogram) | Yes (nomogram) | Yes (risk scoring system + decision tree) | Yes (nomogram) | Yes (risk scoring system) | Yes (regression equation) | Yes (nomogram) | Yes (regression equation) | Yes (AutoRAS-UTI scoring system) |
| Full model coefficients reported | Partial | Yes | Yes (HR/OR table) | Yes | Yes | Partial (labelling inconsistency) | Partial (tree structure) | Yes | Yes | Yes | Yes | Yes | Yes |
| Authors' main conclusion | Nomogram incorporating 5 predictors demonstrated good discrimination and calibration for predicting CAUTI in stroke inpatients | Nomogram demonstrated excellent discriminative ability; clinically useful for SCI surgical patients | XGBoost-based nomogram provides acceptable CAUTI prediction with good external validity across institutions | Nomogram provides reference tool for CAUTI risk prediction in elderly ICU patients | Nomogram demonstrated moderate-to-good discrimination; clinically feasible for daily CAUTI risk assessment | Nomogram provides practical reference for CAUTI prevention in post-hysterectomy patients | Decision tree model provides visual, easy-to-use CAUTI screening tool for NICU patients | Nomogram effectively identifies high-risk CAUTI patients in NICU setting | Risk scoring system provides simple, quantitative bedside tool for CAUTI prediction in gynaecological malignancy patients | Prediction model demonstrates acceptable accuracy for CAUTI prediction in ED catheterised patients | Nomogram provides a convenient clinical tool for predicting CAUTI in TBI patients | Logistic regression model identifies 8 independent risk factors for CAUTI in neurosurgical patients | AutoRAS-UTI provides daily-updated automated CAUTI risk assessment with good prospective validation |
| Authors' stated limitations | Single centre; retrospective design; no external validation | Small sample size; single centre; short follow-up | Retrospective; administrative outcome definition; limited to Taiwan population | Single centre; retrospective design; elderly ICU only | Single centre; retrospective case-control design | Single centre; retrospective design; Chinese-specific population | Small event count; single centre; retrospective design | Small event count; single centre; retrospective design | Single centre; retrospective; no prospective validation | Single centre; retrospective case-control; no external validation | Single centre; retrospective; no external validation | Single centre; retrospective; case-control design; no external validation | Single institution; administrative data may misclassify some cases |
| Overall risk of bias (PROBAST) | High | High | High | High | High | High | High | High | High | High | High | High | High |
| Primary driver of high risk | D4 (EPV 7.1; temporal leakage in 2 of 5 predictors) | D4 (EPV 3.2; AUC 0.993 indicates severe overfitting; temporal leakage) | D1 (administrative outcome surrogate); D2 (outcome definition validity) | D2 (temporal leakage: catheter dwell time, no. of catheterisations); D4 (EPV borderline) | D1 (matched case-control distorts baseline risk); D2 (75% of predictors time-varying) | D4 (EPV 4.6; LASSO requires ≥100; temporal leakage in catheter dwell time) | D4 (EPV 1.3; 21 events; critically inadequate for CRT); D2 (temporal leakage in multiple predictors) | D4 (EPV 1.4; 17 training events; 4 validation events); D2 (NICU LOS OR=272.2) | D2 (temporal leakage in catheter dwell time and bladder irrigation); D4 (pre-screening inflates true EPV) | D1 (case-control design; missing data not reported); D2 (temporal leakage) | D2 (temporal leakage in catheter dwell time and ICU stay); D4 (EPV 11.4) | D1 (1:3 sampling artificially inflates event rate); D4 (EPV 5.3; temporal leakage) | D2 (cumulative end-of-stay predictors constitute temporal leakage); D4 (selection from 4,200 candidates with WOE-IV) |
| Applicability concerns | Population restricted to stroke; single Chinese tertiary centre | Population restricted to SCI surgical patients; small N | Limited to Taiwan EHR system; administrative outcome | Population restricted to elderly ICU; single Chinese tertiary centre | General hospitalised patients; case-control limits applicability assessment | Population restricted to post-radical hysterectomy cervical cancer patients | Population restricted to NICU; same cohort as LI Yuping 2023 | Population restricted to NICU; same cohort as LIU Yuting 2023 | Population restricted to gynaecological malignancy post-op patients | Population restricted to ED; case-control design | Population restricted to TBI patients | Population restricted to neurosurgical patients; case-control | Single Korean tertiary institution; scoring system requires daily automated data extraction |

Abbreviations: CAUTI = catheter-associated urinary tract infection; CAASB = catheter-associated asymptomatic bacteriuria; CRT = classification and regression tree; CHAID = chi-squared automatic interaction detection; DCA = decision curve analysis; DI-VNN = domain-informed visible neural network; EHR = electronic health record; ED = emergency department; EPV = events per variable; GCS = Glasgow Coma Scale; H-L = Hosmer–Lemeshow; ICU = intensive care unit; IV = information value; LASSO = least absolute shrinkage and selection operator; LOS = length of stay; LR = logistic regression; MAE = mean absolute error; ML = machine learning; MNAR = missing not at random; MoH = Ministry of Health; N/A = not applicable; NHSN = National Healthcare Safety Network; NICU = neurosurgical intensive care unit; NR = not reported; RF = random forest; RR = ridge regression; SCI = spinal cord injury; SHAP = SHapley Additive exPlanations; TCM = traditional Chinese medicine; TBI = traumatic brain injury; U = unclear; WOE = weight of evidence; XGBoost = extreme gradient boosting.

Notes on cohort overlap: LIU Yuting 2023 and LI Yuping 2023 were derived from the same cohort (Subei People's Hospital, Yangzhou University; n=537; 21 CAUTI events). These reports describe distinct modelling approaches (decision tree versus logistic regression nomogram) and are therefore retained as separate model reports. However, they are counted as a single cohort in all predictor frequency analyses and population-level summaries.
